# Early, medium and long-term mental health in cancer survivors compared with cancer-free comparators: matched cohort study using linked UK electronic health records

**DOI:** 10.1016/j.eclinm.2024.102826

**Published:** 2024-09-16

**Authors:** Harriet Forbes, Helena Carreira, Garth Funston, Kirsty Andresen, Urvita Bhatia, Helen Strongman, Esha Abrol, Liza Bowen, Ceinwen Giles, Krishnan Bhaskaran

**Affiliations:** a Department of Non-Communicable Diseases Epidemiology, London School of Hygiene and Tropical Medicine, London, UK; b Wolfson Institute of Population Health, Barts and The London School of Medicine and Dentistry, Queen Mary University of London, London, UK; c Department of Public Health and Policy, London School of Hygiene and Tropical Medicine, London, UK; d UK Addictions and Related-Research Group, Sangath, India; e Division of Psychiatry, University College London, UK; f Population Health Research Institute, St George’s, University of London, London, UK; g Shine Cancer Support, Poole, Dorset, UK

**Keywords:** Cancer, Mental health, Depression, Anxiety, Non-fatal self-harm, Completed suicide

## Abstract

**Background:**

We aimed to compare the risk of incident depression, anxiety, non-fatal self-harm and completed suicide in survivors from a wide range of cancers versus cancer-free individuals.

**Methods:**

We used electronic health records from the United Kingdom Clinical Practice Research Datalink linked to cancer registry data, hospital admissions data and death records between 1998 and 2021. Adult survivors of the 20 most common cancers were matched (age, sex, general practice) 1:10 to cancer-free individuals. Cox regression models, adjusted for shared risk factors, were used to estimate associations between cancer survivorship and mental health outcomes.

**Findings:**

853,177 adults with cancer diagnosed in 1998–2018 were matched to 8,106,643 cancer-free individuals. Survivors of all 20 cancer types under study had a higher risk of experiencing a new episode of anxiety and depression during follow-up compared with cancer-free individuals; there was also evidence of raised risks of non-fatal self-harm in 17/20 cancers and completed suicide in 8/20 cancers. Effect sizes were greatest in cancers with poorer 5-year survival: hazard ratios (HRs) for anxiety and depression of 1.1–1.2 were seen for malignant melanoma survivors, while HRs for both outcomes were >2.5 for lung and oesophageal cancer survivors. HRs were highest in the first year from cancer diagnosis, reducing over time since diagnosis. However, 5-year cancer survivors still experienced elevated risks of a subsequent new episode of anxiety or depression, in 18/20 cancers.

**Interpretation:**

Survivors of the 20 most common cancers were at increased risk of experiencing depression and anxiety, and these increased risks persisted in medium-to long-term cancer survivors. Substantially raised risks of non-fatal self-harm and completed suicide were also seen for several types of cancer. The risks of all mental health outcomes were generally higher in survivors of cancers with poorer prognosis. Our findings suggest a need for improved psychological support for all patients with cancer.

**Funding:**

10.13039/100010269Wellcome Trust.

**Research in context**

**Evidence before this study**

We searched Ovid MEDLINE(R) for epidemiological studies, reviews and guidelines published in English up to June 24, 2024, using search terms for each mental health outcome (depression, anxiety, non-fatal self-harm or suicide) and cancer, and searched reference lists of relevant articles. We identified articles that provided estimates comparing risks of mental health outcomes between adult survivors of one or more site-specific cancers and controls without a history of cancer. Several cohort and cross-sectional studies have shown that the prevalence of anxiety and depression is generally higher among people with a history of cancer than in the general population, but estimates vary widely due to a number of factors, such as the type and stage of cancer. Whilst studies tend to show the highest risk is in the year following diagnosis, the longer term impact of cancer on depression and anxiety has received less attention; few studies have examined the risk of depression and anxiety among cancer survivors >5 years post diagnosis and findings are mixed. Non-fatal self-harm following cancer has been poorly investigated; we found one cross-sectional survey from South Korea investigating the risk of non-fatal self-harm (defined as suicide attempts) after cancer, which reported no association. A comprehensive systematic review and meta-analysis of 28 studies on suicide mortality after cancer, published up to 2021 (among over 22 million patients), found significantly increased risk of suicide in cancer survivors compared with the general population, with risk strongly related to cancer type, cancer stage and time since diagnosis; three further studies published since the review concurred with these findings.

**Added value of this study**

Our study is one of the largest and most comprehensive to date to compare risks for several mental health outcomes between adult survivors of multiple site-specific cancers and cancer-free comparators, with a consistent methodological approach that allowed us to reveal detailed patterns of risk. We found that survivors of most site-specific cancers had increased risk of depression, anxiety and non-fatal self-harm, but patterns of risk varied by cancer type. There remained an increased risk of experiencing new episodes of anxiety and depression for many cancer types even >5 years after diagnosis. Survivors of some cancer types were at greater risk of suicide. The risks of adverse mental health outcomes were generally higher for people with poorer-prognosis cancer types, and later stage at diagnosis cancers.

**Implications of all the available evidence**

The available evidence to date suggests raised risks of mental health problems among survivors of a wide range of cancers, not only immediately after cancer diagnosis, but also among those surviving more than 5 years from cancer diagnosis. Impacts might be minimised through raising awareness among patients and clinicians, and through appropriate prevention and management strategies.

## Introduction

Around 1 in 2 individuals diagnosed with cancer in the United Kingdom live for over 10 years from their diagnosis,^1^ and survival rates continue to improve. A cancer diagnosis and its treatment can lead to severe psychological distress and/or mental health problems through multiple mechanisms. Cancer survivors may experience distress due to adverse physical consequences of cancer and its treatments, changes to work and family life after their diagnosis, fear of recurrence,^2^ or direct neuropsychiatric effect (e.g., cancers involving the brain). Cancer survivors have highlighted short and long-term psychological impacts of cancer as a research priority.^3^

Despite the potential impact of cancer on subsequent mental health, evidence on the epidemiology of mental health outcomes in cancer survivors is limited. Research to date has mostly focused on short-term risks in the period immediately after cancer diagnosis, with a recent review finding a lack of high-quality research on longer-term risks, a lack of data on all but the most common cancers, and little evidence on younger cancer survivors.^4^ Studies to date have also tended to focus on data from hospital or other treatment settings rather than community and general practice settings, which are likely to be the first point of contact for many mental health problems during survivorship.^5^ Although suicide risk has been shown to be elevated in cancer survivors, we have little understanding of how prior mental health history affects post-cancer suicide risk, due to a lack of data in previous studies.^6^

To address these limitations in the evidence base, we used real-world data from UK electronic health records (EHRs) to investigate early-, medium- and long-term risks of anxiety, depression, non-fatal self-harm (NFSH) and completed suicide in cancer survivors, compared to cancer-free comparators.

## Methods

### Study design and data source

We conducted a matched population-based cohort study, including survivors from the 20 most common cancers in the UK (representing 90% of all cancer diagnoses^7^) and cancer-free individuals from the same data source. We used the UK Clinical Practice Research Datalink (CPRD), which contains anonymised medical records from consenting general practices that use Vision (GOLD)^8^ and EMIS Web (Aurum)^9^ software systems. Over the last ten years, there has been a reduction in general practices (GPs) in England using Vision, in favour of EMIS; data quality is however similar across databases.^10^ They cover >20% of the UK population and are broadly representative of the UK population in respect of age, sex, and ethnicity.^11^^,^^12^ The data include information on demographics, lifestyle-factors, symptoms, diagnoses and prescriptions and CPRD carry out completeness and consistency checks to ensure data quality. We also used linked data on hospital admissions from Hospital Episode Statistics Admitted Patient Care (HES APC), cancer registrations from National Cancer Registration and Analysis Service (NCRAS), death registrations from the Office for National Statistics (ONS), and patient-linked postcode-based Index of Multiple Deprivation (IMD) data. HES data sources are collected for clinical purposes however by 2011 96% of primary diagnoses were shown to be accurate.^13^ Mortality and cancer data are considered to be of high quality, completeness and coding accuracy.^14^^,^^15^ These linkages were only available for practices in England, which restricted the geographical coverage of our study.

### Study population

We identified individuals with an incident diagnosis of one of the 20 most common cancers from NCRAS, namely: oral cavity, oesophageal, stomach, colorectal, liver, pancreatic, lung, malignant melanoma, breast (female), cervical, uterine, ovarian, prostate, kidney, bladder, central nervous system (CNS), thyroid, non-Hodgkin lymphoma (NHL), multiple myeloma and leukaemia. Eligible cancer survivors were diagnosed between 2nd January 1998–31st December 2018, aged ≥ 18, and had no history of other cancers (besides non-melanoma skin cancer (NMSC)). We also required the cancer diagnosis to be ≥ 1-year after the start of research-quality follow-up in GOLD (determined by CPRD on the basis of practice and patient-level data quality checks) and one year after registration in Aurum,^11^^,^^12^ to ensure there was sufficient follow-up for baseline characteristics to be recorded and to ensure that the observed cancer diagnosis code reflected incident diagnosis. Cancer survivors were eligible to be cancer-free comparators prior to the date of their cancer diagnosis (“index date”), at which point their follow-up within the control group was censored.

Matching was done without replacement and cancer survivors diagnosed earliest in calendar time were matched first (greedy matching approach), to avoid time-related biases. For each cancer survivor, we randomly selected up to 10 cancer-free comparators from the overall unexposed population, matched at index date on birth year ( ± 3 years, with closer matches given preference), sex and GP practice; comparators had to be under follow-up and have no cancer history (besides NMSC) on their index date (i.e., on the diagnosis date of the matched cancer case). The matching ratio of 1:10 was chosen as a compromise between maximising precision and minimising exclusions of some patients due to running out of available matches. Similar to cancer survivors, cancer-free comparators were required to have ≥1-year continuous registration prior to index date.

### Exclusion criteria

Practices contributing to both CPRD GOLD and CPRD Aurum were removed from the CPRD GOLD population. We excluded individuals with: history of severe mental illness at index date (schizophrenia, other psychotic disorders and bipolar disorders); missing smoking status (<5% of cancer cases had missing smoking status); or unknown IMD (<0.1%). For each mental health outcome, people experiencing that outcome in the year before index date were excluded to ensure individuals were not likely to be experiencing an ongoing outcome episode at the start of follow-up, and that outcomes observed during follow-up were new episodes. We did not exclude people with an earlier history of mental illness (i.e. >1 year before index date) as they were considered a key group of interest, with potential vulnerability to cancer-related stressors.

### Study measures

Outcomes: We identified depression, anxiety and NFSH (with or without suicidal intent) following cancer diagnosis using the clinical coding systems utilized in the various databases; specifically, Read codes in CPRD GOLD; SNOMED CT (UK edition), Read codes and local EMIS web codes in CPRD Aurum; and ICD-10 codes in HES APC. Completed suicides were identified using relevant ICD-9 and ICD-10 codes listed as causes of death in ONS death registration data. We identified antidepressant and anti-anxiety medication prescriptions in each year following index date (defined as having ≥1 day prescription in that year). Full details on these definitions are in Supplementary Methods S1.

Covariates: Age, sex, GP practice, IMD deprivation and ethnicity were defined at index date. The most recent records prior to index date were used to define smoking, problematic alcohol use, body mass index (BMI) categories, and history of depression, anxiety, or NFSH. Cancer stage at diagnosis (from NCRAS) was defined as early and late stage. Full definitions of covariates are in Supplementary Methods S2 and code lists are here [https://github.com/beyondcancer/BC_mental_health_after_cancer/tree/main/codelists].

### Statistical analysis

We described patient characteristics overall and by cancer type at index date. We described number of events observed, person-years at risk and outcome rates, for each cancer type and outcome. The association between cancer type and each outcome was quantified using Cox regression models stratified on matched set, with time since index date as the underlying time-scale. Follow-up began at index date and ended at the earliest of: an observed outcome, death, transfer out of the practice, end of data collection from practice, second primary cancer diagnosis (from NCRAS) and study end (29th March 2021, end of HES and ONS data coverage). Follow-up within matched sets continued as long as at least one cancer survivor and one cancer-free comparator remained under follow-up. “Crude” hazard ratios (HRs) (i.e. accounting for matching factors only) were first computed, then we adjusted for smoking, patient-level IMD (using twentiles of IMD) and problematic alcohol use, to explore the role of shared risk factors (see Supplementary Methods S3 for depiction of assumed causal relationships). BMI and ethnicity had substantial missingness (10.1% and 28.8% respectively in comparators), so were excluded from our main models; this complete case analysis approach relies on the assumption that missingness of these variables is independent of the outcome, given the covariates.^16^ By censoring the competing risk of death, our analysis focussed on the cause-specific hazard.^17^ All hypothesis tests were two-sided at significance level of 0.05.

To explore the hypothesis that cancers with poorer survival probabilities might have stronger associations with mental health outcomes, we displayed our fully adjusted HRs for mental health outcomes against 5-year predicted net survival for each cancer type, using published survival statistics from 2010 to 2011^18^ where available; for cancer types not described, specifically thyroid, liver and oral cavity cancers, we used survival statistics published elsewhere.^19^

We created adjusted cumulative incidence curves to reflect the observed cumulative incidence of mental health outcomes in cancer survivors and the expected cumulative incidence in the absence of cancer history, stratified by prior history of mental illness (as the absolute incidence of mental health outcomes was highest in those with a history of mental illness). This was done by first fitting a Royston-Parmar model among cancer survivors and controls (separately by cancer site) with the covariates from the fully adjusted Cox model, and with the baseline hazard modelled using a spline with four degrees of freedom.^20^ The survival function was predicted from this model for every cancer survivor and averaged to produce cumulative incidence curves among those with and without a history of mental illness.^21^ To produce the standardised comparison curves representing expected cumulative incidence in the absence of cancer, the survival functions were predicted and averaged again but with cancer survivorship status set to 0. The curves were therefore standardised to the covariate distribution of the cancer survivor group.

Interaction terms were added to our adjusted Cox models to explore effect modification by age (18–49; 50–59 and ≥60 years), calendar year of diagnosis (1998–2002 and 2003–2018, prior to and after the introduction of Cancer Care Reviews [a conversation between a patient recently diagnosed with cancer and primary care practitioner soon after a cancer diagnosis]^22^), ethnicity (White and non-White), sex, history of the outcome, patient-level deprivation (low [IMD 1, 2 or 3] and high [IMD 4 or 5]) and region (North, East, West, South or London). We investigated whether early or late-stage cancer at diagnosis was associated with a greater risk of mental health outcomes, restricting the analysis to 2012 onwards, when cancer stage data are deemed reliable enough for research.^23^^,^^24^ We examined whether associations between cancer survivorship and mental health outcome varied by length of follow-up (which implicitly checks the proportional hazards assumption) by re-estimating HRs in our adjusted Cox models with follow-up censored at 1, 2, 5, and 10 years. This method of starting follow-up from zero is preferable to calculating time-stratified hazard ratios.^25^

We then explored the association between cancer survivorship and risk of experiencing a new *episode* of anxiety, depression or NFSH, or a completed suicide, in medium to long-term cancer survivors. For this investigation, we re-ran the main analysis, starting follow-up at 1, 3, 5 and 10 years after index date among those still alive and under follow-up. In each of these analyses, only those who experienced that outcome in the year before the new start of follow-up were excluded; as in the main analysis, this was to ensure that individuals were not likely to be experiencing an ongoing outcome episode at the start of follow-up, and that outcomes observed during follow-up would reflect a new episode of the disease [see Supplementary Methods S4 for a diagram of the cohorts for each of these analyses].

Finally, we explored ongoing burden of adverse mental health by examining medication use over time. For each year following study entry we calculated the proportion of cancer survivors and cancer-free comparators using antidepressants and anti-anxiety medications in that year; this was done for each cancer type, standardised for age and sex (using the age and sex distribution of all cancers and years combined as the standard).

### Sensitivity analyses

We tested how robust our findings were in multiple sensitivity analyses: (i) additionally adjusting for ethnicity derived from the primary care record among those with complete data; (ii) additionally adjusting for ethnicity derived from the primary care record, with missingness included as a category (iii) additionally adjusting for ethnicity derived from the primary or secondary care record among those with complete data (iv) additionally adjusting for BMI category among those with complete data; (v) additionally adjusting for BMI category, with missingness included as a category (vi) excluding those with the outcome ever before index date; (vii) using a more specific outcome definition (depression and anxiety only) which excluded symptom, monitoring or checklist codes; (viii) limiting the comparator group to recent consulters (≥1 face-to-face consultation for any reason in the year before index date) to reduce the potential for ascertainment bias; (ix) additionally adjusting for history of mental illness (depression, anxiety or NFSH) before index date and (x) censoring follow-up on 31st January 2020 (onset of COVID-19 pandemic, when primary care contacts for mental health conditions reduced^26^).

We decided not to formally adjust for multiple comparisons due to difficulties in defining appropriately objective groupings of hypotheses for adjustment, and a risk that adjustments would be over-conservative and based on unrealistic assumptions.^27^

Statistical analyses were done in Stata SE, version 17.

### Ethics statement

The study protocol was approved by CPRD’s Research Data Governance group (Ref: 20_000268) and the London School of Hygiene and Tropical Medicine’s Research Ethics Committee (Ref: 28,518). Individual participant consent is not required.

### Role of the funding source

The funder of the study had no role in study design, data collection, data analysis, data interpretation, or writing of the report.

## Results

### Cohort description

853,177 adults with incident cancer diagnosed between 1998 and 2018 were matched to 8,106,643 cancer-free individuals (Supplementary Results S1).

Median follow-up of cancer survivors from index date was 4.4 years (IQR 0.6–6.7). Demographic and lifestyle-related factors were similar between people with and without cancer (Table 1) and between CPRD GOLD and Aurum databases (Supplementary Results S2). Cancer survivors had more missing data on ethnicity (38.6% versus 28.8%), but fewer missing data on BMI (6.6% versus 10.1%) compared to cancer-free comparators. See Supplementary Results S3 for cohort descriptions by cancer type.

**Table 1:** Baseline characteristics of individuals with cancer and their matched general population cancer-free comparators.

	Cancer survivors	Cancer-free comparators
Total	853,177 (100.0)	8,106,643 (100.0)
Total person-years included∗ (millions)	3.75	56.05
Age (years)		
18–39	26,981 (3.2)	256,853 (3.2)
40–59	179,630 (21.1)	1,725,342 (21.3)
60–79	463,867 (54.4)	4,474,536 (55.2)
≥80	182,699 (21.4)	1,649,911 (20.4)
Sex		
Male	424,868 (49.8)	3,984,352 (49.1)
Female	428,309 (50.2)	4,122,291 (50.9)
Index of multiple deprivation		
1 (least deprived)	192,650 (22.6)	1,872,635 (23.1)
2	186,785 (21.9)	1,808,131 (22.3)
3	169,203 (19.8)	1,620,870 (20.0)
4	158,890 (18.6)	1,481,680 (18.3)
5 (most deprived)	145,649 (17.1)	1,323,327 (16.3)
Ethnicity		
White	336,600 (39.5)	4,051,306 (50.0)
South Asian	11,087 (1.3)	171,560 (2.1)
Black	10,643 (1.2)	113,692 (1.4)
Other	2833 (0.3)	40,227 (0.5)
Mixed	1957 (0.2)	23,510 (0.3)
Missing	490,057 (57.4)	3,706,348 (45.7)
Calendar year		
1998–2000	53,550 (6.3)	481,149 (5.9)
2001–2005	179,972 (21.1)	1,686,055 (20.8)
2006–2010	229,041 (26.8)	2,197,890 (27.1)
2011–2015	244,345 (28.6)	2,343,749 (28.9)
2016–2018	146,269 (17.1)	1,397,800 (17.2)
Consultations in year prior to index date		
0	44,307 (5.2)	992,324 (12.2)
1–3	80,169 (9.4)	1,561,401 (19.3)
4–9	235,162 (27.6)	2,490,157 (30.7)
≥10	493,537 (57.8)	3,062,684 (37.8)
Smoking status		
Non-smoker	255,430 (29.9)	2,716,334 (33.5)
Current smoker	147,829 (17.3)	1,440,090 (17.8)
Ex-smoker	449,918 (52.7)	3,950,219 (48.7)
Problematic alcohol use		
No	826,130 (96.8)	7,888,084 (97.3)
Yes	27,047 (3.2)	218,559 (2.7)
BMI category		
Underweight	21,650 (2.5)	137,648 (1.7)
Normal weight	292,165 (34.2)	2,597,784 (32.0)
Overweight	297,474 (34.9)	2,817,968 (34.8)
Obese	185,845 (21.8)	1,738,127 (21.4)
Missing	56,043 (6.6)	815,116 (10.1)
History of mental illness ever		
Anxiety	201,726 (23.6)	1,804,264 (22.3)
Depression	242,339 (28.4)	2,167,794 (26.7)
Non-fatal self-harm	21,113 (2.5)	179,316 (2.2)

### Cancer survivorship and mental health outcomes

**Fig. 1:**
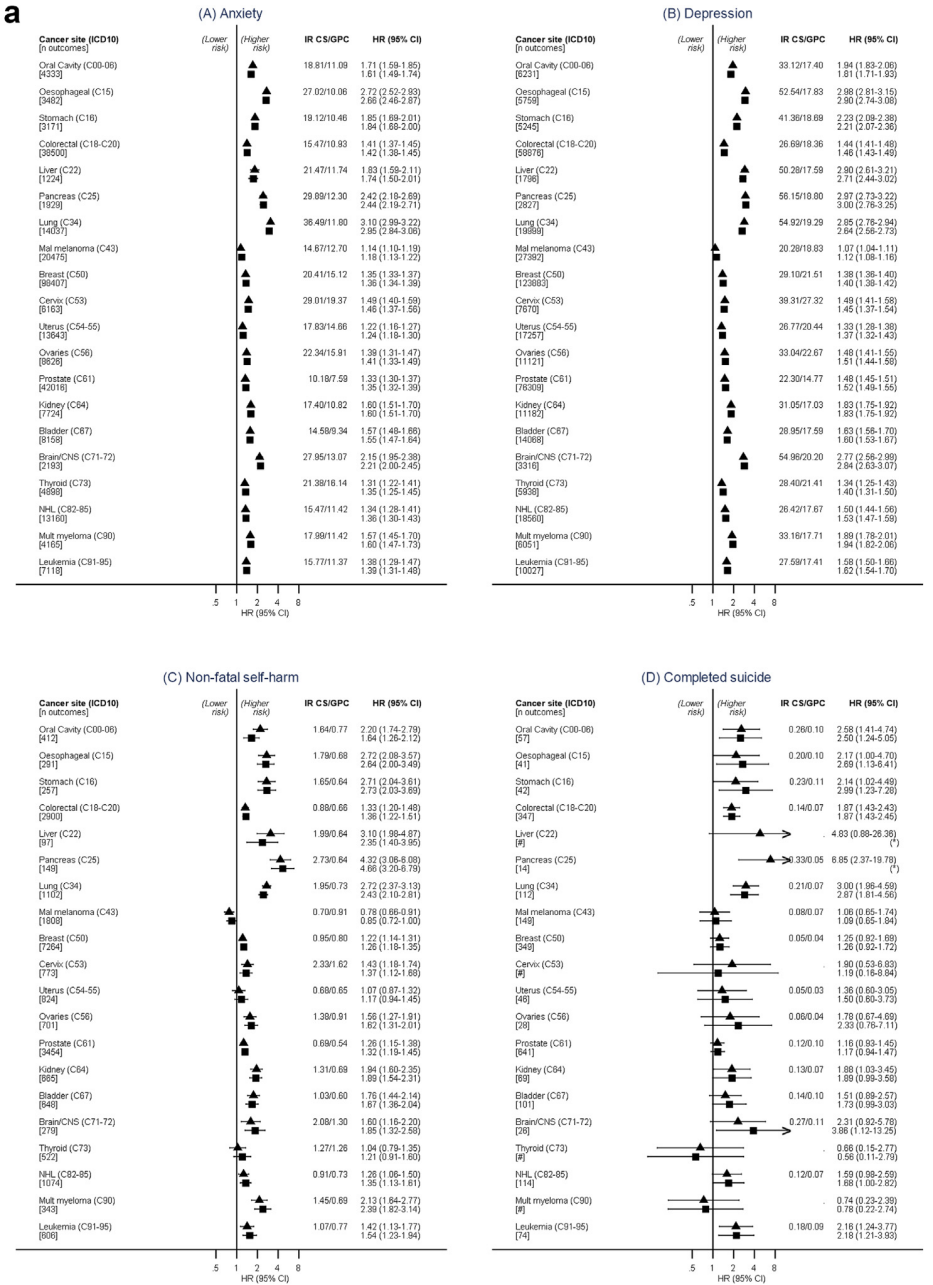

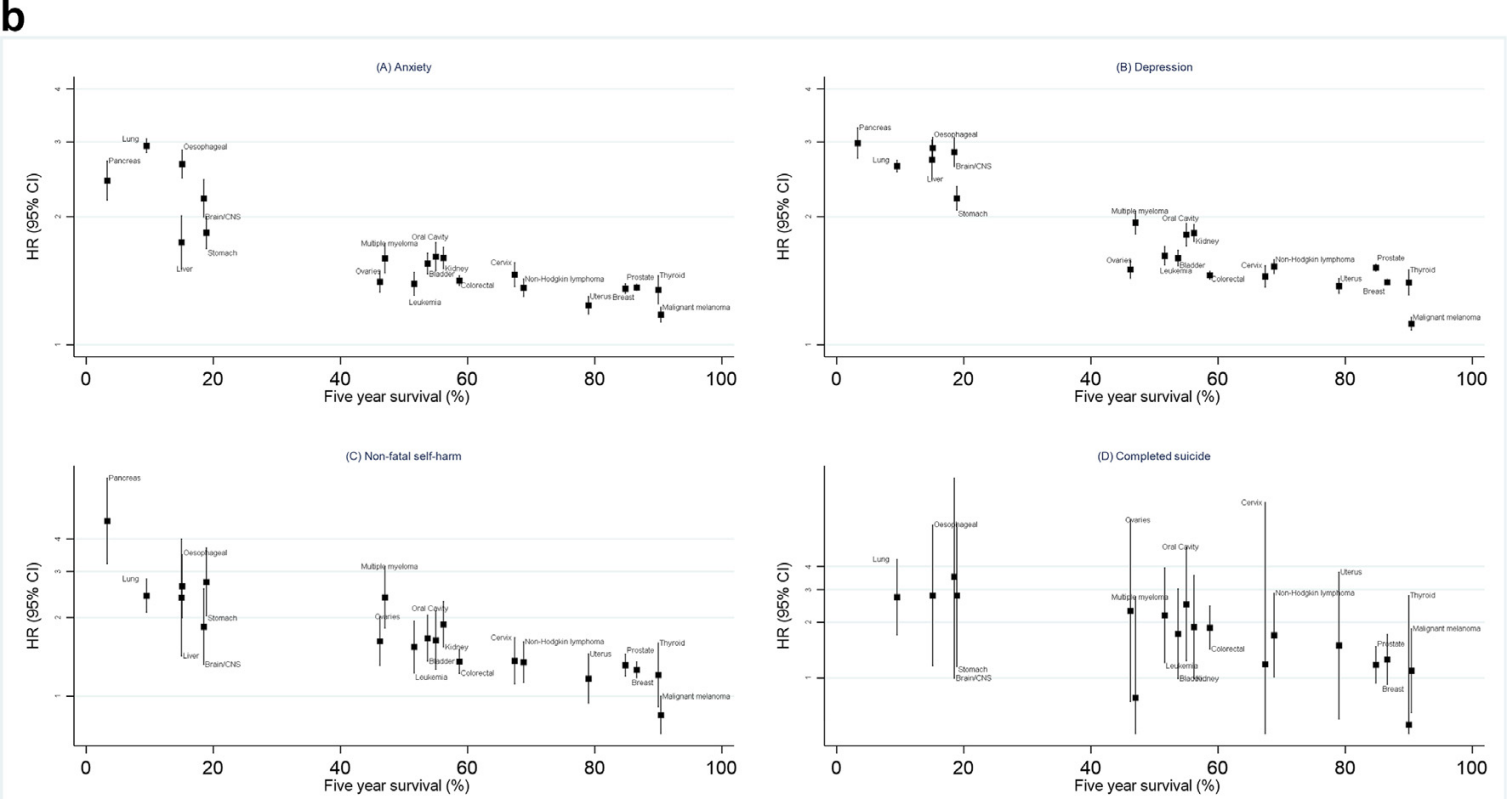
**a: Absolute and relative risks of new episodes of (A) Anxiety (B) Depression (C) Non-fatal self-harm and (D) Completed suicide in the years following cancer diagnosis compared with cancer-free comparators.** Black triangles = Stratified by age, gender and GP practice matched sets; Black squares = Additionally adjusted for shared risk factors. (∗) too few events for estimation. HR = Hazard ratio, CI = Confidence interval. IT Incidence rate per 1000 patient years. GPC = general population controls, CS = cancer survivors. Fully adjusted models also included IMD 20 deciles, smoking status and alcohol problems. #To protect confidentiality, number of outcomes and incidence rates are not presented where cell counts are less than 5. **b: Relative risks of new episodes of (A) Anxiety (B) Depression (C) Non-fatal self-harm and (D) Completed Suicide in the years following cancer diagnosis compared to cancer-free comparators, plotted against five-year survival.** Black squares = Stratified by age, gender and general practice matched sets and additionally adjusted for shared risk factors. HR = Hazard ratio, CI = Confidence interval. P-trends: Anxiety P < 0.001, Depression P < 0.001, Non-fatal self-harm P < 0.001, Completed suicide P < 0.329. ∗5-year survival statistics for each cancer type from Cancer Research UK Summary Survival statistics from 2010 to 2011 and for cancer categories not in these data (specifically thyroid, liver and oral), survival statistics published elsewhere by Cancer Research UK were used.

Crude incidence rates of depression, anxiety, NFSH and completed suicide in cancer survivors and cancer-free comparators are shown in Fig. 1a along with estimated HRs comparing the groups (for numbers included in each analysis see Supplementary Results S4, and for incidence rates stratified by baseline characteristics, see Supplementary Results S5). HRs comparing risk of each outcome in cancer survivors to cancer-free comparators in “crude” (implicitly adjusted for the matching factors) and fully adjusted models were largely similar. Compared to cancer-free comparators, after fully adjusting for confounders, we found evidence of increased risk of depression and anxiety in survivors from all 20 cancer types; adjusted HRs for anxiety ranged from 1.18 (1.13–1.22) in malignant melanoma survivors to 2.94 (2.83–3.05) in lung cancer survivors; adjusted HRs for depression had a similar range (from 1.12, 1.08–1.16 in malignant melanoma survivors to 2.98, 2.75–3.24 in pancreas cancer survivors). The risk of NFSH was increased in survivors of 17/20 cancers. Confidence intervals were wide in the analysis of completed suicide due to relatively small numbers of events, but we found evidence of an increased risk of completed suicide in 8/20 cancers.

The raised risks of all mental health outcomes were consistently highest for cancers with poorer 5-year survival, such as pancreas and lung cancer, and we observed a pattern of decreasing HRs as 5-year survival probability increased, with a significant linear trend for anxiety, depression and NFSH (Fig. 1b).

**Fig. 2:**
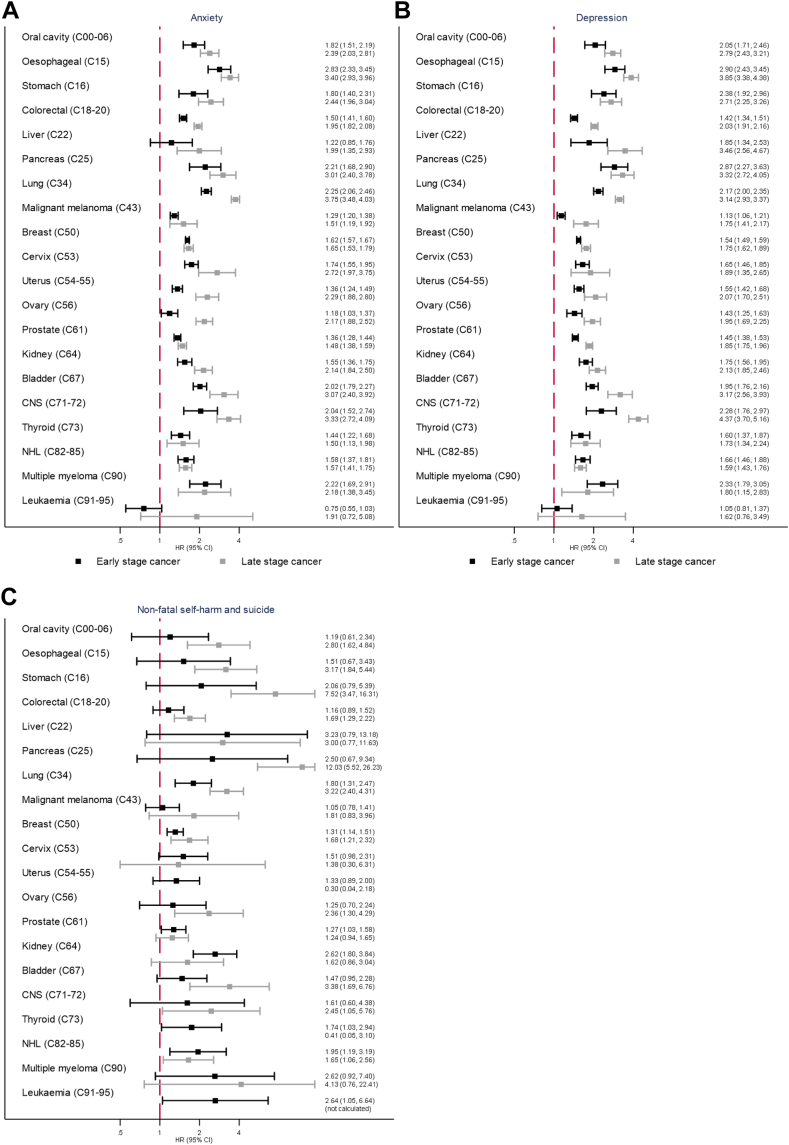
**Relative risks of (A) Anxiety (B) Depression (C) Non-fatal self-harm and completed suicide in the years following cancer diagnosis compared with cancer-free comparators, with cancer survivors categorised as early or late-stage cancer at diagnosis.** Data from 2012 onwards. Black squares = Early-stage cancer. Grey squares = Late-stage cancer. HR = hazard ratio, CI = confidence interval. All HRs stratified by age and gender matched sets and adjusted for IMD 20 deciles, smoking status and alcohol problems. Early stage includes Stage I and II (or for CNS cancers grade 1 and 2, or for leukaemia stage A and B) and late stage includes Stage III and IV (or grade 3 and 4 for CNS cancers, or Stage C for leukaemia, or stage III for myeloma [myeloma has no stage IV]).

The raised risks of anxiety, depression and NFSH/completed suicide (combined due to few events) were generally higher in those with late-stage than early-stage cancer at diagnosis (Fig. 2), however, confidence intervals were wide for NFSH/completed suicide. We also found larger increased risks for males compared to females in 6/20 cancers for anxiety and 10/20 cancers for depression, larger increased risk for non-White White ethnicity in 7/20 cancers for anxiety and 8/20 cancers for depression, larger increased risks for <50 years in 3/20 cancers for anxiety and 4/20 cancers for depression and larger increased risks after 2003 compared to before 2003 for some cancers (Supplementary Results S6). There was no clear evidence of effect modification for the outcomes NFSH and completed suicide; for suicide, too few events for many cancers meant only results for the four most common cancers are presented. For anxiety and depression, the estimated HRs were largest when only the first year of follow-up from index date was included, and weakened as increasing amounts of follow-up were added (Supplementary Results S7).

**Fig. 3:**
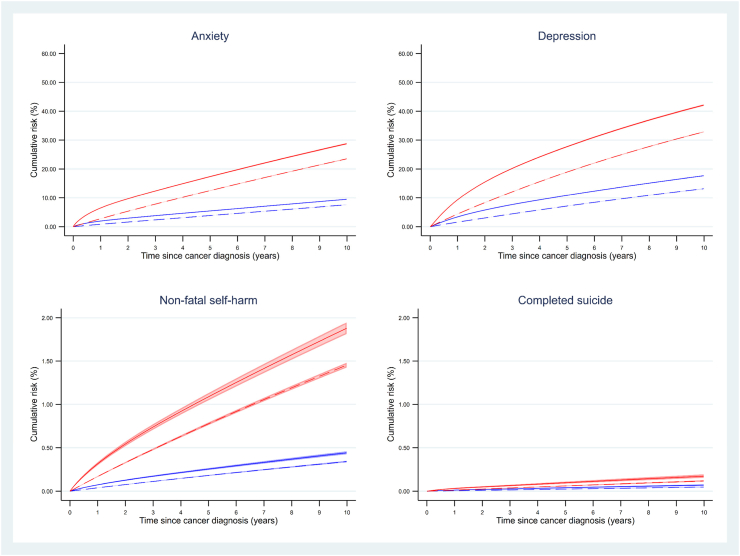
**Cumulative risk of experiencing a new mental health outcome episode in the years following diagnosis of one of the 20 cancers combined, compared with expected risk, by history of mental illness (depression, anxiety or non-fatal self-harm).** Solid red line = cancer survivors, history of mental illness. Solid blue line: Cancer suvivors, no history of mental illness. Dashed red line = Expected cumulative incidence, history of mental illness. Dashed blue line = Expected cumulative incidence, no history of mental illness. Note: the scale on the y-axes differ for each outcome. The survival function was predicted from a Royston-Parmar model among cancer survivors and controls (separately by cancer site) for every participant in the cancer survivor group and averaged to produce cumulative incidence curves among those with and without a history of mental illness. To produce the standardised comparison curves representing expected cumulative incidence in the absence of cancer, the survival functions were predicted and averaged again but with cancer survivorship status set to 0. The curves were therefore standardised to the covariate distribution of the cancer survivor group. The confidence intervals for depression and anxiety were very narrow, therefore are difficult to detect on this graph.

Fig. 3 shows the standardised cumulative risks of anxiety, depression, NFSH and completed suicide in cancer survivors versus expected cumulative risks in the absence of cancer history, stratified by history of mental illness. Consistent with the previous analysis, the cumulative incidence of new mental illness episodes in cancer survivors diverged from the expected risks in the first 1–2 years after index date. Differences remain stable in later years. The largest cumulative incidences were observed for depression: among those with no prior history of mental illness, 10.9% of cancer survivors were estimated to have experienced an episode of depression by 5 years, compared with an expected cumulative incidence of 7.2%; larger cumulative incidences were seen for those with a prior history (27.8% in cancer survivors compared with 19.0% expected, at 5 years). Cumulative incidences of anxiety were slightly lower but showed similar patterns; much lower cumulative incidences were observed for NFSH and suicide.

**Fig. 4:**
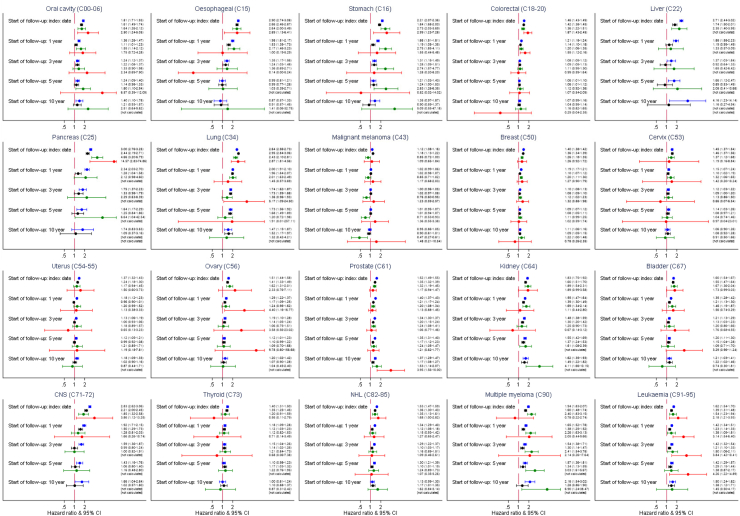
**Adjusted relative risk of mental health outcomes in medium to long-term cancer survivors compared with comparators, starting follow-up at 1, 3, 5 and 10 years following cancer diagnosis.** Blue line = Depression. Black line = Anxiety. Red line = Non-fatal self-harm. Green line = Completed suicide. Right hand column is HRs and 95%CIs. All HRs stratified by age and gender matched sets and adjusted for IMD 20 deciles, smoking status and alcohol problem.

We observed sustained increased risks of new mental health episodes when starting follow-up in medium to long-term cancer survivors, and excluding people who had experienced the outcome in the previous year (Fig. 4), though risks gradually decreased over survival time. Estimates varied by cancer type and outcome. For those surviving ≥5 years from cancer diagnosis, there remained an increased risk of a new episode of depression after this time point for 17/20 cancer types, a new episode of anxiety for 14/20 cancer types and a new episode of NFSH for 7/20 cancers. Five-year leukaemia survivors had evidence of a continuing increased risk of completed suicide but for most cancers, analysis of this outcome lacked precision.

**Fig. 5:**
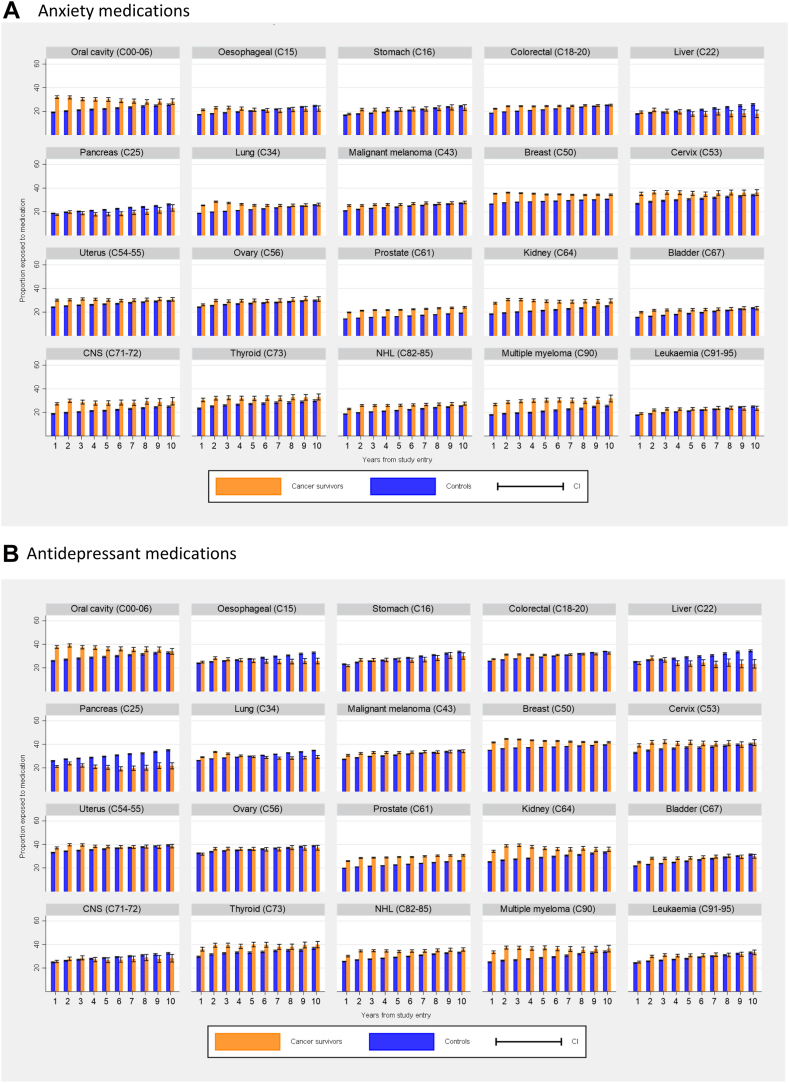
**Proportion of those with and without cancer using (A) anxiety medications and (B) antidepressants in the year since study entry, standardised for age and sex.** Orange bars: Cancer survivors. Red bars: Controls. Error bars: 95% CI.

Use of antianxiety (Fig. 5a) and antidepressants (Fig. 5b) medications were consistently higher in cancer survivors compared to cancer-free comparators throughout the ten years following cancer diagnosis for most cancers.

### Sensitivity analyses

Our sensitivity analyses resulted in no meaningful changes in the HRs. All analyses still showed an increased risk of depression and anxiety across all cancer types. The increased risk of non-fatal self-harm was attenuated and no longer statistically significant for cervical cancer after adjusting for ethnicity in CPRD (though the results was similar to the main analysis when adjusting for ethnicity in CPRD/HES and ethnicity in CPRD with missing data included as a missing category). The increased risk of suicide was no longer observed for oral cavity after adjusting for BMI (though was observed when adjusting for BMI where missing data were included as a missing category), for oesophageal after restricting to recent consulters and for stomach, colorectal, kidney and bladder cancer as well as leukaemia, after adjusting for ethnicity in CPRD (though the results was similar to the main analysis when adjusting for ethnicity in CPRD/HES) (Supplementary Results S8).

## Discussion

In this comprehensive assessment of the risk of mental health outcomes in cancer survivors in England, survivors from the 20 most common cancers in the UK with no evidence of active mental illness in the year prior to cancer diagnosis were at increased risk of experiencing a new episode of depression and anxiety during follow-up, compared with cancer-free individuals. Importantly, in those surviving to 5-years from cancer diagnosis, survivors of 17/20 cancer types remained at higher risk of experiencing a new depression episode after this time point, and survivors of 14/20 cancer types were at increased risk of a new anxiety episode, suggesting a sustained mental health burden. Correspondingly, survivors of most types of cancer had higher antidepressant and anti-anxiety medication use than cancer-free comparators throughout at least 10 years of follow-up after cancer diagnosis. Compared to cancer-free individuals, there was evidence of raised risks of NFSH in 17/20 cancer and completed suicide in 8/20 cancers; five-year survivors of several site-specific cancer types remained at increased risk of NFSH and for leukaemia survivors, suicide. Raised risks of mental health outcomes were more pronounced among people diagnosed with poorer-prognosis cancers.

Our study benefits from access to large numbers of individuals, enabling us to investigate associations in unprecedented detail. The use of consistent methodology across a wide range of cancers and outcomes gives confidence that the observed patterns of risk reflect real phenomena, rather than methodological artefacts. A major strength is that NCRAS data has very high validity (positive predictive value and sensitivity) for identifying cancer^23^ with accurate dating, and staging information. This study contains one of the first population-based analyses of mental health outcomes by cancer stage at diagnosis, a key prognostic factor. Finally, as primary care is the first point of contact for anyone experiencing mental health episodes, our study captures mental health outcomes in all those seeking support.

Our study also has some limitations. We estimated associations between multiple cancer types and mental health outcomes, increasing the risk of false positive associations; therefore, individual effect estimates with wide CIs and close to the null should be interpreted cautiously.

Some data were not available or not of sufficient quality to use in this study. For example, whilst we have some records of psychological therapy, some people may self-refer for these treatments in the UK, whilst others may seek private treatment due to the long wait times. As such, we chose not to assess use of psychotherapy over time, as we did with prescriptions of antidepressants and anti-anxiety medications. Finally, the electronic health records data used in this study rarely records social and socioeconomic factors that can be important determinants of both mental health and cancer. For example, there is little or no data on finance, employment, and education. Marital status and living arrangements are recorded for some individuals, however there is a great deal of missingness (∼70% in those over 65 years^28^) therefore the data were not of sufficient quality to include. As such there may be some residual confounding by social factors. However, we were able to adjust for socioeconomic status, and this made little difference to the results, suggesting any residual confounding is likely to be minimal.

One of our key findings is that the risk of mental health issues persists many years from diagnosis. However this analysis required us to start follow-up many years from diagnosis; as such it is be based on individuals diagnosed earlier in calendar time and will include a greater proportion of follow-up from later in calendar time (because follow-up cannot be included till after years of registration). This adds two important caveats to this part of our results: (i) if cancer experiences and treatments have changed over time, then generalisibility of our results for “long-term survivors” to more contemporary cancer survivor populations should be cautious; and (ii) the comparability of our results that started from different points in survivorship may be affected by changes over calendar time in the diagnosis/capture of mental health issues.

Cancer survivors may get diagnosed with mental health outcomes more frequently than cancer-free comparators due to increased contact with health services, creating a detection bias. Furthermore, it is a national priority to provide cancer survivors with wellbeing support^29^ and from 2003 GPs were incentivised to conduct wellbeing reviews within six months of diagnosis. However, these reviews have not been implemented systematically,^30^ observed associations persisted beyond six months after diagnosis, prescriptions for mental health were also higher in the cancer cohort and associations were seen prior to 2003, suggesting detection bias is not driving these findings.

Misclassification of mental health outcomes may have resulted from incomplete information being registered in the primary care record. Studies in breast cancer survivors using EHRs report lower prevalence of anxiety/depression than studies using patient-reported outcomes.^31^ Survivors may be accessing cancer-specific mental health support through the voluntary sector.^32^ A UK-based study, among 608 breast cancer survivors and cancer-free women found many self-reporting anxiety and depression in postal questionnaires had no corresponding evidence in EHRs.^33^ However, this study did not report any meaningful differences between breast cancer survivors and controls, so whilst we may underestimate the absolute risks of anxiety/depression after cancer, the relative effect estimates are likely to be unbiased.

Identifying distinct episodes of mental illness is also challenging using EHRs, as episodes will rarely be recorded as resolved. We addressed this by requiring no record of the outcome in the 365 days before start of follow-up, however, we cannot be certain a mental health outcome reflects a new episode. Further, whilst we attempted to capture any history of mental illness by using data prior to patients entering CPRD (e.g. transferred in from non-CPRD GPs) or before the data was deemed of sufficient quality for research, there may be variable capture of mental health prior to individuals start of follow-up. This may have led to misclassification of history of mental health, though it is unlikely to be differential according to cancer status.

We allowed cancer survivors to be in the eligible pool of cancer free individuals up to their cancer diagnosis. Whilst this ensures the control pool is fully representative of the cancer free population and avoids time-related biases that may arise from excluding individuals on the basis of a future cancer diagnosis, it may lead to slight overestimates in mental health issues in the cancer free population, if cancer survivors experience more mental health problems in the year prior to their diagnosis. This may have led to slightly underestimated association between cancer and subsequent mental health. However, less than 1% of the control pool went on to be diagnosed with cancer, therefore any impact is likely to be minimal.

Finally, there were some missing data for two potential confounders, BMI and ethnicity. However, when these variables were included in a complete case analysis, the results were broadly similar. Whilst we considered using multiple imputation to account for these missing data, the data fields are very likely to be missing not at random (for example people are more likely to have their BMI recorded in primary care if it is outside the healthy range) therefore the assumptions required for multiple imputation do not hold.

Our findings of increased risk of depression and anxiety after cancer are in line with previous work, though estimates to date vary considerably by type and stage of cancer.^4^ Our study confirms the risk of depression and anxiety is higher in poorer survival cancers and those with late-stage cancer at diagnosis. Few studies have investigated mental health in longer-term cancer survivors using EHRs. Qualitative work highlights the issue of post-treatment mental distress, when survivors have reduced interaction with healthcare providers, whilst adjusting to life after a significant diagnosis.^34^ A systematic review reported that symptoms of anxiety, but not depression, were higher in cancer survivors (all cancer types) compared to healthy controls at ≥2 years from cancer diagnosis.^35^ Our findings may differ as we analysed cancer types separately, rather than combining cancers which may obscure some real associations. Furthermore, we used cancer-free general population comparators, whereas some studies in the systematic review used controls with other illnesses.

The risk of NFSH among cancer survivors compared to healthy controls has not been widely investigated. Population-based studies among cancer survivors have found a high incidence of NFSH after cancer, with younger aged survivors having the highest risk of NFSH.^36^, ^37^, ^38^ Our study clearly demonstrated the risk of NFSH is higher in many cancer-types, compared to cancer-free individuals.

We showed the risk of completed suicide was highest in cancers with poorer survival and during the first year after diagnosis, in line with previous evidence.^6^ There was evidence of raised suicide risk in 8/20 cancers, however due to limited power we cannot exclude the possibility of associations with the other cancers. Our study suggested the risk of suicide in leukaemia survivors remained increased for those alive at 5 years post-cancer diagnosis; whilst the confidence intervals were wide, this may reflect leukaemia being harder to cure, and requiring multiple rounds of longer-term treatment.

This study highlights a need for psychological support for cancer survivors, both in the first year from diagnosis and in subsequent years. Targeted support could be considered for cancer survivors at greatest risk of adverse mental health outcomes, such as those with poorer survival or a history of mental illness. A better understanding of specific points in the cancer journey when patients are at the highest risk of developing mental health issues could help inform when intervention is most needed. Finally, further research to understand the drivers of mental health issues would help target prevention strategies. Cancer itself and its treatments can have varied side effects, including chronic pain, lymphedema, sexual dysfunction, poor mobility, financial instability or lack or employment, all of which could profoundly affect the lives of survivors, potentially leading to mental health issues.^39^

In conclusion, survivors of all types of cancer were at increased risk of depression and anxiety, and the risk of new episodes persisted among long-term cancer survivors (those surviving 5 years after cancer diagnosis) for many cancer types. Survivors of most cancer types are at increased risk of NFSH, and completed suicide risk is doubled in survivors of some cancers. Survivors of cancers with poorer prognosis generally had a greater risk of developing mental health outcomes.

## Contributors

KB had the idea and supervised all aspects. All authors were involved in the study design. HF and HC accessed and verified the data. HF did the data management, analysis and wrote the first draft. All authors reviewed and contributed to the final manuscript.

## Data sharing statement

This study is based in part on data from the Clinical Practice Research Datalink obtained under licence from the UK Medicines and Healthcare products Regulatory Agency. The terms of our licence to access the data preclude us from sharing individual patient data with third parties. The raw data may be requested directly from CPRD following their usual procedures.

## Declaration of interests

Helen Strongman is funded by the National Institute for Health Research (NIHR) though an Advanced Fellowship (NIHR301730). The views expressed in this publication are those of the author(s) and not necessarily those of the NIHR, NHS or the UK Department of Health and Social Care. All other authors declare no conflicts of interest.

## Appendix ASupplementary data

Supplementary Methods S1-S3 and Results S1-S8

## References

[1] Cancer Research UK Cancer statistics for the UK. http://www.cancerresearchuk.org/health-professional/cancer-statistics-for-the-uk.

[2] Pitman A., Suleman S., Hyde N., Hodgkiss A. (2018). Depression and anxiety in patients with cancer. BMJ.

[3] James Lind Alliance (2018). Priority setting partnerships: living with and beyond cancer. https://www.jla.nihr.ac.uk/priority-setting-partnerships/living-with-and-beyond-cancer/.

[4] Niedzwiedz C.L., Knifton L., Robb K.A., Katikireddi S.V., Smith D.J. (2019). Depression and anxiety among people living with and beyond cancer: a growing clinical and research priority. BMC Cancer.

[5] Mitchell A.J., Chan M., Bhatti H. (2011). Prevalence of depression, anxiety, and adjustment disorder in oncological, haematological, and palliative-care settings: a meta-analysis of 94 interview-based studies. Lancet Oncol.

[6] Heinrich M., Hofmann L., Baurecht H. (2022). Suicide risk and mortality among patients with cancer. Nat Med.

[7] Cancer Research UK (2023). Cancer incidence for common cancers. https://www.cancerresearchuk.org/health-professional/cancer-statistics/incidence/common-cancers-compared.

[8] Clinical Practice Research Datalink (2022). Clinical practice research Datalink.

[9] Clinical Practice Research Datalink (2022). Clinical practice research Datalink.

[10] Jick S., Vasilakis-Scaramozza C., Persson R., Neasham D., Kafatos G., Hagberg K.W. (2023). Use of the CPRD Aurum database: insights gained from new data quality assessments. Clin Epidemiol.

[11] Herrett E., Gallagher A.M., Bhaskaran K. (2015). Data resource profile: clinical practice research Datalink (CPRD). Int J Epidemiol.

[12] Wolf A., Dedman D., Campbell J. (2019). Data resource profile: clinical practice research Datalink (CPRD) Aurum. Int J Epidemiol.

[13] Boyd A.C.R., Johnson L., Simmonds S. (2017).

[14] Henson K.E., Elliss-Brookes L., Coupland V.H. (2020). Data resource profile: national cancer registration dataset in England. Int J Epidemiol.

[15] Mathers C.D., Fat D.M., Inoue M., Rao C., Lopez A.D. (2005). Counting the dead and what they died from: an assessment of the global status of cause of death data. Bull World Health Organ.

[16] White I.R., Carlin J.B. (2010). Bias and efficiency of multiple imputation compared with complete-case analysis for missing covariate values. Stat Med.

[17] Andersen P.K., Geskus R.B., de Witte T., Putter H. (2012). Competing risks in epidemiology: possibilities and pitfalls. Int J Epidemiol.

[18] Cancer Research UK (2014). https://www.cancerresearchuk.org/health-professional/cancer-statistics/survival#heading-Four.

[19] Cancer Research UK (2022). https://www.cancerresearchuk.org/about-cancer.

[20] Royston P., Parmar M.K. (2002). Flexible parametric proportional-hazards and proportional-odds models for censored survival data, with application to prognostic modelling and estimation of treatment effects. Stat Med.

[21] Lambert Paul C. (2017). https://pclambert.net/software/standsurv/standardized_survival/.

[22] Gopal D.P., Ahmad T., Efstathiou N., Guo P., Taylor S.J.C. (2023). What is the evidence behind cancer care reviews, a primary care cancer support tool? A scoping review. J Cancer Surviv.

[23] Strongman H., Williams R., Bhaskaran K. (2020). What are the implications of using individual and combined sources of routinely collected data to identify and characterise incident site-specific cancers? a concordance and validation study using linked English electronic health records data. BMJ Open.

[24] United Kingdom and Ireland Association of Cancer Registries (UKIACR) (2018). http://www.ukiacr.org/kpis.

[25] Hernán M.A. (2010). The hazards of hazard ratios. Epidemiology.

[26] Mansfield K.E., Mathur R., Tazare J. (2021). Indirect acute effects of the COVID-19 pandemic on physical and mental health in the UK: a population-based study. Lancet Digit Health.

[27] Althouse A.D. (2016). Adjust for multiple comparisons? It’s not that simple. Ann Thorac Surg.

[28] Jain A., van Hoek A.J., Walker J.L., Mathur R., Smeeth L., Thomas S.L. (2017). Identifying social factors amongst older individuals in linked electronic health records: an assessment in a population based study. PLoS One.

[29] National Health Service (2019).

[30] Watson E.K., Sugden E.M., Rose P.W. (2010). Views of primary care physicians and oncologists on cancer follow-up initiatives in primary care: an online survey. J Cancer Surviv.

[31] Carreira H., Williams R., Müller M., Harewood R., Stanway S., Bhaskaran K. (2018). Associations between breast cancer survivorship and adverse mental health outcomes: a systematic review. J Natl Cancer Institute.

[32] Macmillan Cancer Support (2024). https://www.macmillan.org.uk/cancer-information-and-support/get-help/emotional-help/bupa-counselling-and-emotional-well-being-support.

[33] Helena C., Rachael W., Harley D., Krishnan B. (2022). Recording of patients’ mental health and quality of life-related outcomes in primary care: a cross-sectional study in the UK. BMJ Open.

[34] Aitken L.A., Hossan S.Z. (2022). The psychological distress and quality of life of breast cancer survivors in sydney, Australia. Healthcare (Basel).

[35] Mitchell A.J., Ferguson D.W., Gill J., Paul J., Symonds P. (2013). Depression and anxiety in long-term cancer survivors compared with spouses and healthy controls: a systematic review and meta-analysis. Lancet Oncol.

[36] Noel C.W., Eskander A., Sutradhar R. (2021). Incidence of and factors associated with nonfatal self-injury after a cancer diagnosis in ontario, Canada. JAMA Netw Open.

[37] Men V.Y., Emery C.R., Lam T.C., Yip P.S.F. (2022). Suicidal/self-harm behaviors among cancer patients: a population-based competing risk analysis. Psychol Med.

[38] Chang W.H., Lai A.G. (2022). Cumulative burden of psychiatric disorders and self-harm across 26 adult cancers. Nat Med.

[39] Macmillan Cancer Support (2013).

