# Supplementary material for: Early, medium and long-term mental health in cancer survivors compared with cancer-free comparators: matched cohort study using linked UK electronic health records
Source: eClinicalMedicine. 2024 Sep 16;76:102826. doi: 10.1016/j.eclinm.2024.102826 (PMC11421364; doi:10.1016/j.eclinm.2024.102826)
Supplement: Supplementary Methods S1-S3 and Results S1-S8 [file mmc1.docx]

**Supplementary Methods and Results**

for paper titled, “Early, medium and long-term mental health in cancer survivors compared with cancer free comparators: matched cohort study using linked UK electronic health records.”

[SUPPLEMENTARY METHODS 2](#_Toc195352722)

[S1 Methods Definition of outcomes 3](#_Toc375417936)

[Search and selection of diagnostic codes and pharmacological drugs 3](#_Toc1568004626)

[Anxiety 3](#_Toc1550461412)

[Depression 5](#_Toc1294077267)

[Non-fatal self-harm 7](#_Toc481287621)

[Completed Suicide 8](#_Toc1148155601)

[S2 Methods Definition of covariates 8](#_Toc781167739)

[S3 Methods Direct Acyclic Diagram (DAG) depicting the causal relationships between cancer and mental health outcomes. 11](#_Toc261274410)

[sUPPLEMENTARY Results 13](#_Toc1128630549)

[S1 Results Flowchart of the selection of the cohorts used in analysis (from (A) CPRD Aurum and (B) CPRD GOLD separately) 14](#_Toc577353908)

[S2 Results Baseline characteristics of individuals with cancer and their matched general population comparators, stratified on individuals within CPRD GOLD and CPRD Aurum 16](#_Toc206764043)

[S3 Results Characteristics of cancer survivors and matched controls from the general population (site-specific cancer/control cohorts) 18](#_Toc461520495)

[Oral Cavity (C00-06) 18](#_Toc2022783265)

[Oesophagus (C15) 19](#_Toc774140244)

[Stomach (C16) 20](#_Toc1023972194)

[Colorectal (C18-20) 21](#_Toc893733852)

[Liver (C22) 22](#_Toc124528286)

[Pancreas (C25) 23](#_Toc1133219855)

[Lung (C34) 24](#_Toc1748475483)

[Malignant melanoma (C43) 25](#_Toc821704602)

[Breast (C50) 26](#_Toc1781721001)

[Cervix (C53) 27](#_Toc1070015196)

[Uterus (C54-55) 28](#_Toc473948183)

[Ovary (C56) 29](#_Toc1404669186)

[Prostate (C61) 30](#_Toc311570149)

[Kidney (C64) 31](#_Toc46373779)

[Bladder (C67) 32](#_Toc1486223223)

[Central nervous system (CNS, C71-72) 33](#_Toc2005391793)

[Thyroid (C73) 34](#_Toc1272307298)

[Non-Hodgkin Lymphoma (NHL, C82-85) 35](#_Toc1201449663)

[Multiple myeloma (C90) 36](#_Toc1998757470)

[Leukaemia (C91-C95) 37](#_Toc1370315884)

[S4 Results Numbers of cancer survivors and cancer-free controls included in each analysis 38](#_Toc1763311125)

[S5 Results Absolute incidence of mental health outcomes in cancer survivors and comparators, by baseline characteristics 39](#_Toc1967942713)

[S6 Results Relative risk of mental health outcomes in cancer survivors compared with general population comparators, with effect modification. 40](#_Toc926408405)

[S7 Results Role of time since cancer diagnosis in the associations between cancer survivorship and mental health outcomes in individuals with history of cancer compared to cancer-free individuals. 45](#_Toc1446767493)

[S8 Results Sensitivity analyses 47](#_Toc1870162475)

[Depression 48](#_Toc1675415075)

[Anxiety 50](#_Toc1827866036)

[Non-fatal self-harm 52](#_Toc1662018907)

[Completed suicide 54](#_Toc129383187)

[References 56](#_Toc743623422)

# **SUPPLEMENTARY METHODS**

# **S1 Methods** Definition of outcomes

## Search and selection of diagnostic codes and pharmacological drugs

We updated previously defined code lists,[1] by searching dictionaries of codes using keywords defined by a general practitioner experienced in using the codes in clinical practice (GF). Where available we compared to previously defined code lists. Three researchers (HF, GF and HC) independently assigned each code to a certainty group, compared assignments and agreed the final list of codes.[available here: https://github.com/beyondcancer/BC_mental_health_after_cancer/tree/main/codelists]

Drugs for anxiety and depression were identified in product dictionaries by searching formulations listed in the British National Formulary (BNF) as indicated to treat these conditions. The final list of products was checked for suitability by a GP (GF) and irrelevant products were excluded.

Codes indicating a history of the outcomes were retained for exclusion when identifying first ever disease.

### Anxiety

We looked for evidence of anxiety in primary care records and inpatient HES data. Anxiety was defined with diagnosis codes only, if the code was considered sufficiently specific (referred to as definite or probable codes). When the code was less specific (flagged as “possible” anxiety), for example it referred to typical symptoms of anxiety which are not necessarily pathological, we considered the patient to have anxiety only at the date of their possible code if they had been prescribed with a drug or referred to psychological therapy within 90 days of code for anxiety. Finally anyone with an anti-anxiety prescription and a previous definite code or less specific code for anxiety was considered to have anxiety at the prescription date (in order to capture those on long-term medication, but who may not have recently had a diagnostic code entered into their record).

Diagnostic codes for the following conditions were included/excluded from our definition:

| **Included** | **Excluded** |
| --- | --- |
| Generalised anxiety disorder | Specific phobias (e.g. heights) |
| Panic disorder | Somatic symptoms disorder |
| Mixed anxiety and depression |  |
| Obsessive compulsive disorders * |  |
| Trauma- and stress-related disorders* with anxiety, including PTSD, acute stress disorder, acute stress reaction and adjustment disorder with anxiety |  |
| Anxiety disorder, NOS |  |
| Agoraphobia |  |
| Social anxiety disorder |  |

N.B. ‘Included’ and ‘Excluded’ refer to *symptoms* and *diagnoses* of the conditions listed. PTSD = Post-traumatic stress disorder; NOS = not otherwise specified.

* In DSM-5, published in 2013, obsessive-compulsive and stress-related disorders are classified separately from anxiety disorders. This was a major change from previous editions of the DSM, in which these two categories were considered as anxiety disorders. The data for this study refer to patients under observation during 1998 and 2018 (or part of this period); it is unclear how, or if, the changes in nosology affected the use of codes by GPs at the point of patient care. In addition, the accuracy of the codes to identify each of the sub conditions is likely to be sub-optimal at any given point in time. For these reasons, we decided to include obsessive-compulsive and stress-related disorders in our definition of anxiety. See below, in the depression section, a note about adjustment disorders with anxiety.

Drugs indicated to treat anxiety according to the British National Formulary:

| **Substance name** |  |
| --- | --- |
| Alprazolam | Ketazolam |
| Amitriptyline hydrochloride/ Chlordiazepoxide | Lorazepam |
| Bromazepam | Meprobamate |
| Buspirone hydrochloride | Moclobemide |
| Chlordiazepoxide hydrochloride | Oxazepam |
| Citalopram | Paroxetine hydrochloride |
| Clobazam | Pericyazine |
| Diazepam | Perphenazine |
| Duloxetine hydrochloride | Pregabalin |
| Escitalopram oxalate | Sertraline |
| Fluoxetine | Trazodone hydrochloride |
| Fluvoxamine | Trifluoperazine hydrochloride |
|  | Venlafaxine hydrochloride |

We removed drugs which required rectal or intravenous administration, drugs used for short-term use in anxiety (eg Prochlorperazine).

International Classification of Diseases, 10^th^ revision, codes for anxiety:

| **ICD-10 codes** | **Description** |
| --- | --- |
| F40 | Phobic anxiety disorders |
| F41 | Other anxiety disorders |
| F42 | Obsessive-compulsive disorder |
| F43 | Reaction to severe stress, and adjustment disorders |
| F44 | Dissociative [conversion] disorders |
| F48 | Other neurotic disorders |

Definition used in sensitivity analysis to encompass definite diagnoses:

| **Included (codes for diagnoses only)** | **Excluded** |
| --- | --- |
| Generalized anxiety disorder | All symptom codes (e.g. ‘anxious’) |
| Panic disorder | All scores/scales/checklist codes |
| Mixed anxiety and depression |  |
| Obsessive-compulsive disorder |  |
| Trauma- and stress-related disorders with anxiety, including PTSD and acute stress disorder, |  |
| Anxiety disorder, NOS |  |
| Agoraphobia |  |

PTSD = Post-traumatic stress disorder; NOS = not otherwise specified.

### Depression

We looked for evidence of depression in primary care records and inpatient HES data. We used diagnosis codes alone to classify patients with depression, if the code was considered sufficiently specific (referred to as definite or probable codes). When the code referred to typical symptoms of depression that could not be sufficient to classify as a depressive episode (referred to as possible codes), we checked whether there was a prescription of a drug commonly used to depression, or psychological therapy, within 90 days, and considered patients to be depressed at the date of their possible code if yes. Finally anyone with an antidepressant prescription and a previous definite code or less specific code for depression was considered depressed at the prescription date (in order to capture those on long-term medication, but who may not have recently had a diagnostic code entered into their record).

Diagnosis codes for the following conditions were included/excluded from our definition:

| **Included** | **Excluded** |
| --- | --- |
| Major depressive disorder | Bipolar and related disorders (incl. bipolar I, II and cyclothymic disorder) |
| Dysthymia | Premenstrual dysphoric disorder |
| Recurrent depressive disorder | Suicide ^†^ |
| Seasonal affective disorder | Self-harm ^†^ |
| Mixed anxiety and depression | Maternal depression |
| Disruptive mood dysregulation disorder |  |
| Depression in dementia (or other condition) |  |
| Trauma- and stress-related disorders* with depressed mood, including adjustment disorders with depressed mood* |  |
| Mood affective disorder unspecified |  |

N.B. ‘Included’ and ‘Excluded’ refer to symptoms and diagnoses of the conditions listed.

* See note on anxiety table. Adjustment disorders are considered to be a short-term reaction to a stressor (i.e. diagnosed usually within 3 months of the onset of the stressor). The core symptoms of adjustment disorders overlap with those of the anxiety and depressive disorders, which would be diagnosed if the symptoms persist for longer than a 6-month period after the terminus of the stressor. The potential for misclassification between adjustment and depressive disorders is high, as they share the same symptomatology and treatment. To avoid misclassification of the outcome, we included adjustment disorders in our definitions of anxiety and depression.

† Self-harm and Completed Suicide most often occur in patients with a depressive disorder. We will examine these two outcomes separately.

Drugs indicated to treat depression according to the British National Formulary, with additional products no longer in use included:

| **Substance name** |  |  |
| --- | --- | --- |
| Agomelatine | Fluvoxamine maleate | Paroxetine hydrochloride |
| Amitriptyline | Imipramine hydrochloride | Phenelzine sulfate |
| Amoxapine | Iprindole | protriptyline hydrochloride |
| Butriptyline | Iproniazide | Reboxetine mesilate |
| Citalopram | Isocarboxazid | Sertraline |
| Clomipramine hydrochloride | Lofepramine hydrochloride | Sertraline hydrochloride |
| Desipramine | Maprotiline hydrochloride | Tranylcypromine sulfate |
| Dosulepin Hydrochloride | Mianserin hydrochloride | Trazodone Hydrochloride |
| Doxepin hydrochloride | Mirtazapine | Trimipramine maleate |
| Duloxetine hydrochloride | Moclobemide | Venlafaxine |
| Escitalopram oxalate | Nefazodone hydrochloride | Vortioxetine hydrobromide |
| Fluoxetine hydrochloride | Nortriptyline |  |
|  |  |  |
|  |  |  |

International Classification of Diseases, 10^th^ revision codes for depression:

| **ICD-10 codes** | **Description** |
| --- | --- |
| F32 | Depressive episode |
| F33 | Recurrent depressive disorder |
| F34 | Persistent mood [affective] disorders |
| F41.2 | Mixed anxiety and depressive disorder |
| F92.0 | Depressive conduct disorder |

Definition used in sensitivity analysis including specific diagnoses:

| **Included (codes for diagnoses only)** | **Excluded** |
| --- | --- |
| Depressive episode | All symptom codes (e.g. ‘depressed’) |
| Major depression | Monitoring codes |
| Seasonal affective disorder | Scales/Checklists |
| Dysthymia |  |
| Mixed anxiety and depression |  |

### Non-fatal self-harm

We looked for evidence of non-fatal self-harm in primary and secondary care data. We included codes for intentional self-harm, using an updated version of a previously validated list of codes. [2]

We excluded intentional alcohol poisoning/overdose, as these may not indicate self-harm (as done in a previous study)[3]. We included self-harm codes where intent was undetermined. Codes for overdose which specified drugs commonly implicated in Completed Suicide, such as antidepressants and analgesics, were also categorized as definite self-harm.

International Classification of Diseases, 10^th^ revision codes for self-harm:

| **ICD-10 codes** | **Description** |
| --- | --- |
| X60-X84 | Intentional self-harm |
| Y10-Y34 | Event of undetermined intent |
| Y87.0 | **Sequelae of intentional self-harm** |
| Y87.2 | **Sequelae of events of undetermined intent** |

### Completed Suicide

We looked for evidence of fatal self-harm (i.e completed suicide) in linked official death registration data. Completed Suicide was defined using the ICD-10 codes X60-X84 and Y10-34, excluding Y33.9 where the verdict was pending (for the years 2001-2006) and ICD-9 codes  E950–E959 and E980–E989, excluding E988.8 Codes selected according to methodology used by the ONS: https://www.ons.gov.uk/peoplepopulationandcommunity/birthsdeathsandmarriages/deaths/methodologies/suicideratesintheukqmi

# **S2 Methods** Definition of covariates

| **Variable** | **Definition** | **Derivation** |
| --- | --- | --- |
| Sex | Male, Female | Recorded in primary care record |
| Year of birth |  | Recorded in primary care record |
| Age continuous | Age in years | 01/07/birthyear (Exact date of birth not collected by CPRD to maintain the deidentified nature of the data) |
| Age categorical | Age groups: 18-39, 40-59, 60-79, >=80 | 01/07/birthyear (Exact date of birth not collected by CPRD to maintain the deidentified nature of the data) |
| Ethnicity from CPRD | 5 categories, as defined in the Census: White; Mixed or multiple ethnic groups; Asian or Asian British; Black, African, Caribbean or Black British; Other. | Derived from information in the patient clinical records in analyses of primary care data prior to index date. |
| Ethnicity from CPRD or HES | 5 categories, as defined in the Census: White; Mixed or multiple ethnic groups; Asian or Asian British; Black, African, Caribbean or Black British; Other. | Derived from information in the patient clinical records in analyses of primary care data prior to index date, or if missing in primary care data, derived from hospital data. (https://academic.oup.com/jpubhealth/article/36/4/684/1529704) |
| Patient area-based deprivation | Carstairs Index quintile/decile used as proxy for socioeconomic status (1 least deprived) | Carstairs Index quintile/decile identified by CPRD through third party linkage to patient postcode |
| Region | Value to indicate where in the UK the practice is based. The region denotes the former Strategic Health Authority for practices within England. | Provided by CPRD |
| Rural-urban | Rural or urban (from 2011) | Identified by CPRD through linkage of Rural Urban Classifications for England to practice postcode. |
| BMI | Categorised based on WHO categories for Caucasian populations | Derived from weight and height measures https://pubmed.ncbi.nlm.nih.gov/24038008/ |
| Smoking status | Non-smoker, smoker, exsmoker | Previous structured or coded record of smoking status. Nonsmokers recategorized as ex-smoker if there was a previous record of smoking |
| Problematic alcohol use | Current drinker with high alcohol consumption | Derived from codes in primary care indicating high alcohol use |
| Consultations | number of face-to-face consultations in year before index | Derived from face-face consults in primary care |
| Severe Mental Illness | SMI diagnosis prior to index date | Any previous coded diagnosis in primary care |
| Chemotherapy | Exposure to chemotherapy | Ascertained from SACT drugs only. |

Algorithm to classify cancer stage at diagnosis

Stage of cancer at diagnosis was derived from the UK Cancer Registry data for each cancer diagnosed from 2013 onwards. Stage data in the cancer registry is recorded with the following staging systems:

- **UICC TNM Classification of Malignant Tumours (5^th^, 6^th^, 7^th^, and 8^th^ eds).** Used for oral cavity, oesophageal, stomach, colorectal, liver, pancreas, lung, malignant melanoma, breast, cervix, uterus, ovary, prostate, kidney, bladder and thyroid cancers.
- **FIGO staging system for cervical and ovarian cancer.**
- **Duke’s staging system for colorectal cancer.**
- **Binet staging system** for of chronic lymphocytic leukaemia (CLL).
- **Ann Arbor staging** for lymphomas, both in Hodgkin's lymphoma and non-Hodgkin’s.
- **International Staging System (ISS)** and **Revised ISS (R-ISS)** for multiple myeloma.

For solid tumours, except for brain (see below), when TNM stage was not provided but information on T, N and M were provided, we created a TNM stage using the following logic:

1. Use the cancer stage recorded by the cancer registry (available in the *stage_best* variable) if available. For the gynaecological cancers, this included FIGO stage provided.
2. Assign stage IV to the patient if stage was unavailable from the cancer registry but M (variable *m_best*) is provided and equal to “1”, meaning evidence of distant metastases.
3. Derive TNM stage according to the UICC TNM Classification of Malignant Tumours, 7^th^ ed., if a T, N and M are provided (variables *t_best*, *n_best* and *m_best*), and stage was unavailable after steps 1 and 2. (The 7^th^ edition of TNM was in use during the period for which the bulk of our stage data is available, 2013-2017)
4. If stage is still missing, T and N are provided but M is missing or “X”, we derived TNM stage according to the UICC TNM Classification assuming that M is “0” (meaning no evidence of distant metastases).

CNS cancers: graded not staged

Tumours that begin in the brain are *not* staged, because while they may spread to other parts of the central nervous system, they rarely spread to distant organs or lymph nodes. CNS cancers are therefore given only a grade, not a stage. This was taken from the variable grade variable in cancer registry.

# **S3 Methods** Direct Acyclic Diagram (DAG) depicting the causal relationships between cancer and mental health outcomes.


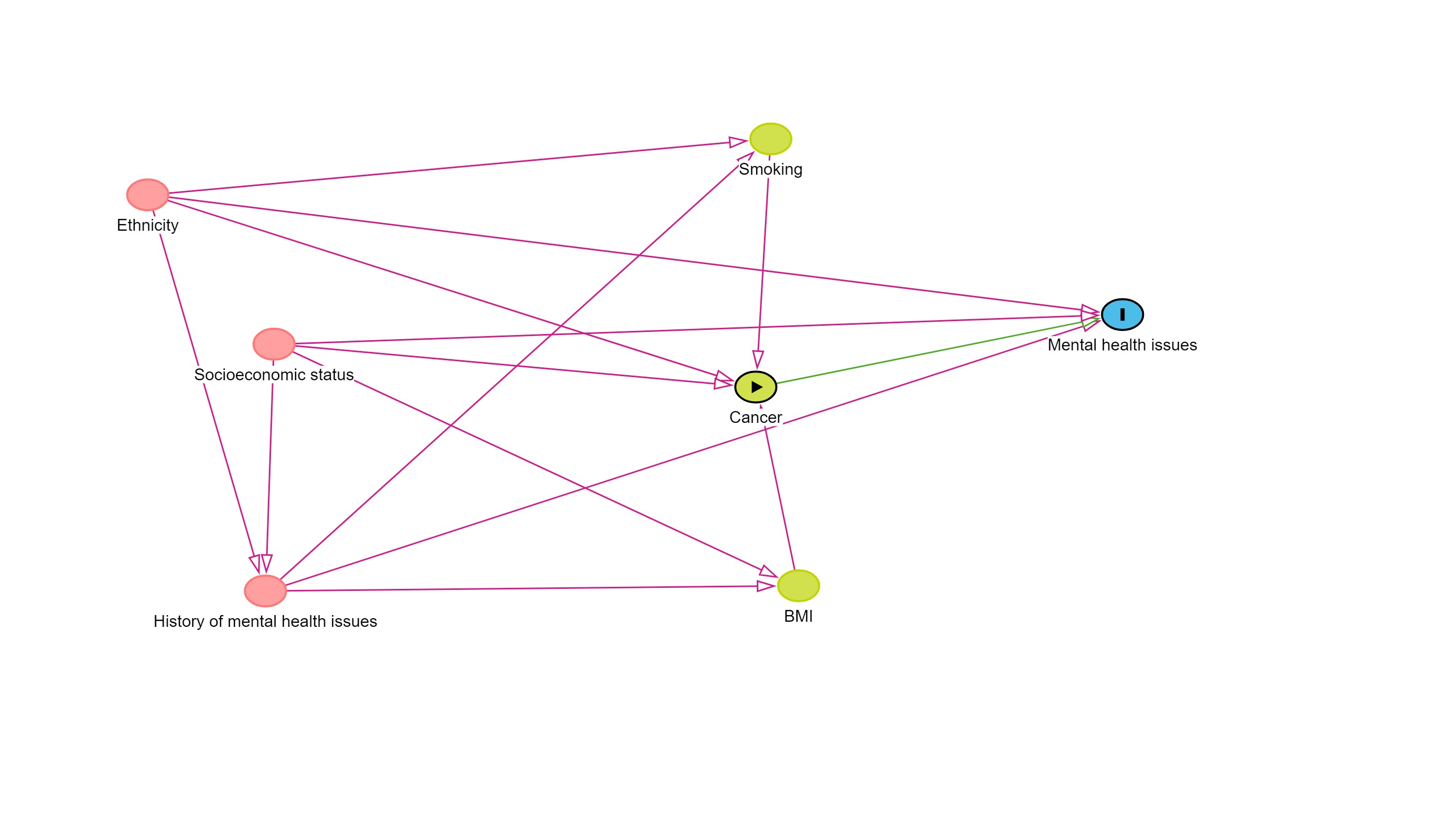

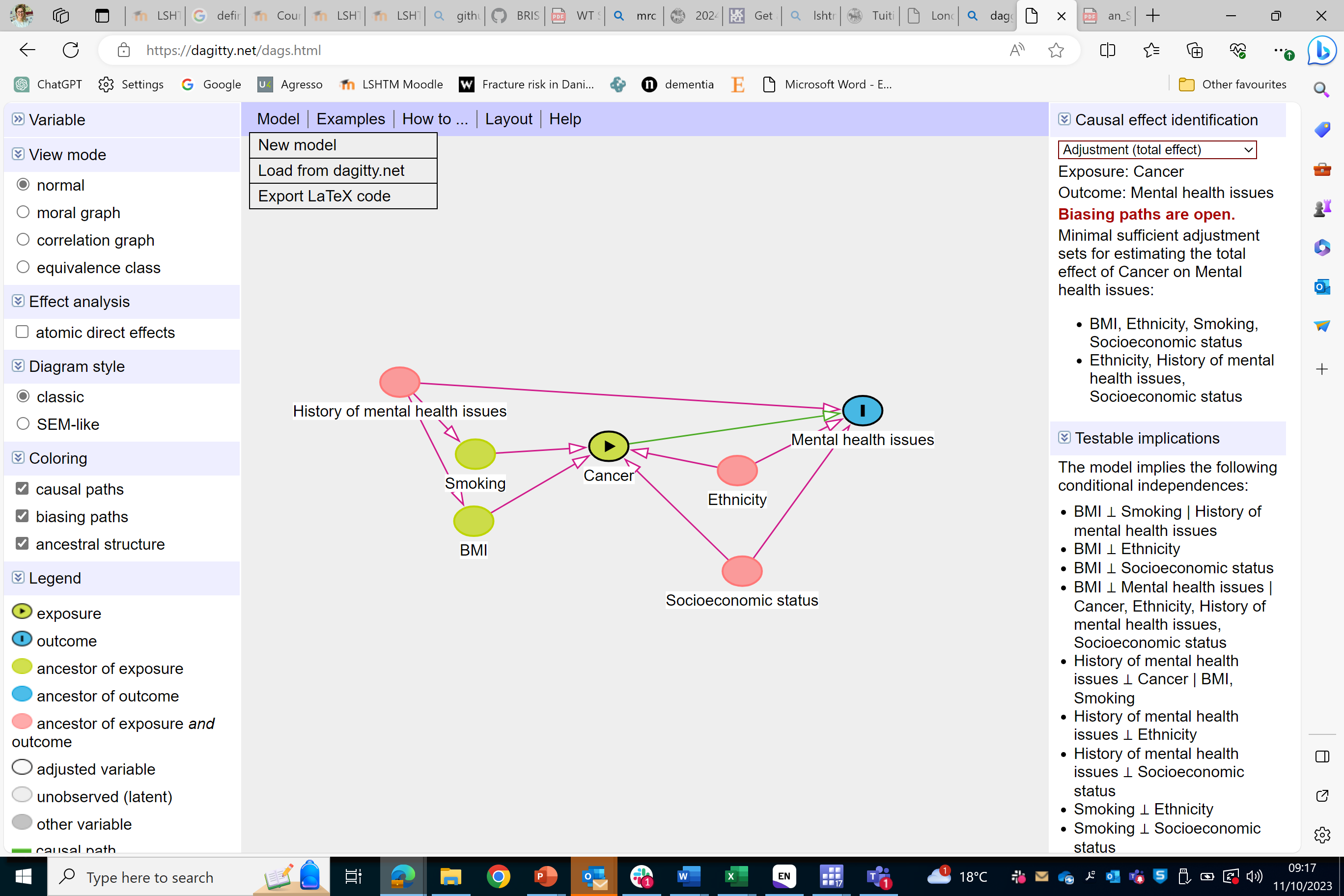


# **S4 Methods** Figure demonstrating the analysis among medium- to long-term cancer survivors (defined as those surviving 1, 3, 5 and 10 years from diagnosis), to examine their risk of experiencing a new episode of anxiety, depression, self-harm or completed suicide.

*To ensure outcomes recorded during follow-up are new episodes

Patient must not have a record of outcome of interest*

Index date

Years since index date

0 1 2 3 4 5 6 7 8 9 10 11 12

Main analysis among all cancer survivors

Index date

Years since index date

0 1 2 3 4 5 6 7 8 9 10 11 12

Analysis among 1-year cancer survivors

Years since index date

0 1 2 3 4 5 6 7 8 9 10 11 12

Analysis among 3-year cancer survivors

Period of follow-up

Index date

Years since index date

0 1 2 3 4 5 6 7 8 9 10 11 12

Analysis among 5-year cancer survivors

Index date

Years since index date

0 1 2 3 4 5 6 7 8 9 10 11 12

Analysis among 10-year cancer survivors

Index date

# **sUPPLEMENTARY Results**

# **S1 Results** Flowchart of the selection of the cohorts used in analysis (from (A) CPRD Aurum and (B) CPRD GOLD separately)

1. **Aurum**

1,864,206 people with at least one malignant tumour (all cancers)

1,657,109 people diagnosed with one of the 20 most common cancers in the UK

Exclusions: 207097 recorded with a cancer other than those eligible for this study

2,871,706 people with a tumour registered in the NCRAS (any behaviour)

Exclusions: 1,007,500 people with *in situ*, benign tumours or non-melanoma skin cancers

Exclusions:

13774 cancer diagnosed in people aged <18 years or >113 years

1908 mismatch of cancer site and sex

817829 cancers diagnosed prior to start of follow up, or after end of follow up, in CPRD Aurum May 2022

823,598 people diagnosed with a first primary cancer of the 20 most common cancers in the UK, 2007-2018

Exclusions:

12895 without matched comparators or in practices with duplicated data

245,842 missing smoking status

6,766 missing IMD

104,489 Severe mental illness

9,153 no eligible follow-up

507,153 No matching cancer cases

29,637 missing smoking status

377 missing IMD

10,887 Severe mental illness

17,669 no eligible follow-up

138 No matching comparators

8,017,459 matched comparators

7,144,056 matched comparators

751,995 cancers

810,703 with cancer

1. **GOLD**

301,202 people with at least one malignant tumour (all cancers)

268893 people diagnosed with one of the 20 most common cancers in the UK

Exclusions: 32309 recorded with a cancer other than those eligible for this study

462,266 people with a tumour registered in the NCRAS (any behaviour)

Exclusions: 161064 people with *in situ*, benign tumours or non-melanoma skin cancers

Exclusions:

1796 cancer diagnosed in people aged <18 years or >113 years

325 mismatch of cancer site and sex

156333 cancers diagnosed prior to start of follow up, or after end of follow up, in CPRD Aurum May 2022

110439 people diagnosed with a first primary cancer of the 20 most common cancers in the UK, 2007-2018

Exclusions:

291 without matched comparators

38,936 missing smoking status

349 missing IMD

13,556 Severe mental illness

404 no eligible follow-up

76,725 no matching cancer patients

5,031 missing smoking status

37 missing IMD

1,408 Severe mental illness

2,474 no eligible follow-up

16 No matching comparators

1,092,557 matched comparators

962,587 matched comparators

101,182 cancers

110,148 with cancer

# **S2 Results** Baseline characteristics of individuals with cancer and their matched general population comparators, stratified on individuals within CPRD GOLD and CPRD Aurum

|  |  | **GOLD** | | **Aurum** | |
| --- | --- | --- | --- | --- | --- |
|  |  | **Cancer survivors** | **Non-cancer participants** | **Cancer survivors** | **Non-cancer participants** |
| **Total** |  | 101182 (100.0) | 962587 (100.0) | 751995 (100.0) | 7144056 (100.0) |
| **Person-years from cancer diagnosis/baseline to end of follow-up** | |  |  |  |  |
|  | Mean (SD) | 3.5 (3.9) | 5.4 (4.2) | 4.5 (4.8) | 7.1 (5.0) |
|  | Median (IQR) | 3.5 (0.5-5.4) | 5.4 (2.1-7.9) | 4.5 (0.6-6.9) | 7.1 (3.2-10.2) |
|  | Range | 0.0-22.2 | 0.0-22.2 | 0.0-22.2 | 0.0-22.2 |
| **Total person-years included* (millions)** | | 3.75 |  |  |  |
|  |  |  |  |  |  |
| **Age (years)** | 18-39 | 2841 ( 2.8) | 26714 ( 2.8) | 24140 ( 3.2) | 230139 ( 3.2) |
|  | 40-59 | 20898 (20.7) | 199774 (20.8) | 158732 (21.1) | 1525568 (21.4) |
|  | 60-79 | 55197 (54.6) | 532371 (55.3) | 408670 (54.3) | 3942165 (55.2) |
|  | >=80 | 22246 (22.0) | 203728 (21.2) | 160453 (21.3) | 1446183 (20.2) |
|  |  |  |  |  |  |
| **Sex** | Male | 50530 (49.9) | 475165 (49.4) | 374338 (49.8) | 3509187 (49.1) |
|  | Female | 50652 (50.1) | 487422 (50.6) | 377657 (50.2) | 3634869 (50.9) |
|  |  |  |  |  |  |
| **Index of multiple deprivation** | 1 (least deprived) | 22120 (21.9) | 214089 (22.2) | 170530 (22.7) | 1658546 (23.2) |
|  | 2 | 21590 (21.3) | 210213 (21.8) | 165195 (22.0) | 1597918 (22.4) |
|  | 3 | 22745 (22.5) | 217915 (22.6) | 146458 (19.5) | 1402955 (19.6) |
|  | 4 | 19526 (19.3) | 181240 (18.8) | 139364 (18.5) | 1300440 (18.2) |
|  | 5 (most deprived) | 15201 (15.0) | 139130 (14.5) | 130448 (17.3) | 1184197 (16.6) |
|  |  |  |  |  |  |
| **Ethnicity** | White | 25313 (25.0) | 311785 (32.4) | 311287 (41.4) | 3739521 (52.3) |
|  | South Asian | 514 ( 0.5) | 8555 ( 0.9) | 10573 ( 1.4) | 163005 ( 2.3) |
|  | Black | 468 ( 0.5) | 5381 ( 0.6) | 10175 ( 1.4) | 108311 ( 1.5) |
|  | Other | 240 ( 0.2) | 3736 ( 0.4) | 2593 ( 0.3) | 36491 ( 0.5) |
|  | Mixed | 0 ( 0.0) | 0 ( 0.0) | 1957 ( 0.3) | 23510 ( 0.3) |
|  | Missing | 74647 (73.8) | 633130 (65.8) | 407508 (54.2) | 2978160 (41.7) |
|  |  |  |  |  |  |
| **Calendar year of diagnosis** | 1998-2000 | 5522 ( 5.5) | 49589 ( 5.2) | 48028 ( 6.4) | 431560 ( 6.0) |
|  | 2001-2005 | 25524 (25.2) | 239329 (24.9) | 154448 (20.5) | 1446726 (20.3) |
|  | 2006-2010 | 35065 (34.7) | 337180 (35.0) | 193976 (25.8) | 1860710 (26.0) |
|  | 2011-2015 | 27132 (26.8) | 260484 (27.1) | 217213 (28.9) | 2083265 (29.2) |
|  | 2016-2018 | 7939 ( 7.8) | 76005 ( 7.9) | 138330 (18.4) | 1321795 (18.5) |
|  |  |  |  |  |  |
| **Consultations in year prior to index date** | 0 | 504 ( 0.5) | 54948 ( 5.7) | 43803 ( 5.8) | 937376 (13.1) |
|  | 1 to 3 | 5121 ( 5.1) | 141844 (14.7) | 75048 (10.0) | 1419557 (19.9) |
|  | 4 to 9 | 21125 (20.9) | 258914 (26.9) | 214037 (28.5) | 2231243 (31.2) |
|  | 10+ | 74430 (73.6) | 506804 (52.7) | 419107 (55.7) | 2555880 (35.8) |
|  |  |  |  |  |  |
| **Smoking status** | Non-smoker | 40967 (40.5) | 445161 (46.2) | 214463 (28.5) | 2271173 (31.8) |
|  | Current smoker | 16816 (16.6) | 129273 (13.4) | 131013 (17.4) | 1310817 (18.3) |
|  | Ex-smoker | 43399 (42.9) | 388153 (40.3) | 406519 (54.1) | 3562066 (49.9) |
|  |  |  |  |  |  |
| **Problematic alcohol use** | No | 98663 (97.5) | 949778 (98.7) | 727467 (96.7) | 6938306 (97.1) |
|  | Yes | 2519 ( 2.5) | 12809 ( 1.3) | 24528 ( 3.3) | 205750 ( 2.9) |
|  |  |  |  |  |  |
| **BMI category** | Underweight | 2467 ( 2.4) | 9379 ( 1.0) | 19183 ( 2.6) | 128269 ( 1.8) |
|  | Normal weight | 35244 (34.8) | 195265 (20.3) | 256921 (34.2) | 2402519 (33.6) |
|  | Overweight | 35344 (34.9) | 201272 (20.9) | 262130 (34.9) | 2616696 (36.6) |
|  | Obese | 20833 (20.6) | 122118 (12.7) | 165012 (21.9) | 1616009 (22.6) |
|  | Missing | 7294 ( 7.2) | 434553 (45.1) | 48749 ( 6.5) | 380563 ( 5.3) |
|  |  |  |  |  |  |
| **History of mental illness** | Anxiety | 17504 (17.3) | 160006 (16.6) | 184222 (24.5) | 1644258 (23.0) |
|  | Depression | 27633 (27.3) | 247418 (25.7) | 214706 (28.6) | 1920376 (26.9) |
|  | Non-fatal self-harm | 2469 ( 2.4) | 21232 ( 2.2) | 18644 ( 2.5) | 158084 ( 2.2) |

# **S3 Results** Characteristics of cancer survivors and matched controls from the general population (site-specific cancer/control cohorts)

### Oral Cavity (C00-06)

|  |  | **Cancer survivors** | **Non-cancer comparators** |
| --- | --- | --- | --- |
| **Total** |  | 12001 (100.0) | 114423 (100.0) |
| **Person-years from cancer diagnosis/baseline to end of follow-up** | |  |  |
|  | Mean (SD) | 4.6 (4.5) | 7.0 (4.9) |
|  | Median (IQR) | 4.6 (1.0-6.9) | 7.0 (3.1-10.0) |
|  | Range | 0.0-22.2 | 0.0-22.2 |
| **Total person-years included* (millions)** | | 0.05 | 0.80 |
|  |  |  |  |
| **Cancer stage at diagnosis** | Early | 2600 (21.7) |  |
|  | Late | 4631 (38.6) |  |
|  | Missing | 4770 (39.7) |  |
|  |  |  |  |
| **Age (years)** | 18-39 | 339 ( 2.8) | 3166 ( 2.8) |
|  | 40-59 | 4040 (33.7) | 38433 (33.6) |
|  | 60-79 | 5914 (49.3) | 56945 (49.8) |
|  | >=80 | 1708 (14.2) | 15879 (13.9) |
|  |  |  |  |
| **Sex** | Male | 7495 (62.5) | 71126 (62.2) |
|  | Female | 4506 (37.5) | 43297 (37.8) |
|  |  |  |  |
| **Index of multiple deprivation** | 1 (least deprived) | 2278 (19.0) | 24647 (21.5) |
|  | 2 | 2392 (19.9) | 23959 (20.9) |
|  | 3 | 2384 (19.9) | 22838 (20.0) |
|  | 4 | 2381 (19.8) | 21932 (19.2) |
|  | 5 (most deprived) | 2566 (21.4) | 21047 (18.4) |
|  |  |  |  |
| **Ethnicity** | White | 4941 (41.2) | 58368 (51.0) |
|  | South Asian | 368 ( 3.1) | 3381 ( 3.0) |
|  | Black | 81 ( 0.7) | 1816 ( 1.6) |
|  | Other | 32 ( 0.3) | 668 ( 0.6) |
|  | Mixed | 15 ( 0.1) | 362 ( 0.3) |
|  | Missing | 6564 (54.7) | 49828 (43.5) |
|  |  |  |  |
| **Calendar year** | 1998-2000 | 595 ( 5.0) | 5306 ( 4.6) |
|  | 2001-2005 | 2278 (19.0) | 21122 (18.5) |
|  | 2006-2010 | 3183 (26.5) | 30469 (26.6) |
|  | 2011-2015 | 3697 (30.8) | 35742 (31.2) |
|  | 2016-2018 | 2248 (18.7) | 21784 (19.0) |
|  |  |  |  |
| **Consultations in year prior to index date** | 0 | 791 ( 6.6) | 17750 (15.5) |
|  | 1 to 3 | 1788 (14.9) | 24508 (21.4) |
|  | 4 to 9 | 3757 (31.3) | 34317 (30.0) |
|  | 10+ | 5665 (47.2) | 37847 (33.1) |
|  |  |  |  |
| **Smoking status** | Non-smoker | 2581 (21.5) | 37290 (32.6) |
|  | Current smoker | 3292 (27.4) | 22208 (19.4) |
|  | Ex-smoker | 6128 (51.1) | 54925 (48.0) |
|  |  |  |  |
| **Problematic alcohol use** | No | 10425 (86.9) | 110333 (96.4) |
|  | Yes | 1576 (13.1) | 4090 ( 3.6) |
|  |  |  |  |
| **BMI category** | Underweight | 546 ( 4.5) | 1618 ( 1.4) |
|  | Normal weight | 4498 (37.5) | 34854 (30.5) |
|  | Overweight | 3847 (32.1) | 41358 (36.1) |
|  | Obese | 2191 (18.3) | 25475 (22.3) |
|  | Missing | 919 ( 7.7) | 11118 ( 9.7) |
|  |  |  |  |
| **History of mental illness** | Anxiety | 3366 (28.0) | 25507 (22.3) |
|  | Depression | 3812 (31.8) | 29992 (26.2) |
|  | Non-fatal self-harm | 579 ( 4.8) | 2850 ( 2.5) |

### Oesophagus (C15)

|  |  | **Cancer survivors** | **Non-cancer comparators** |
| --- | --- | --- | --- |
| **Total** |  | 24430 (100.0) | 233412 (100.0) |
| **Person-years from cancer diagnosis/baseline to end of follow-up** | |  |  |
|  | Mean (SD) | 1.6 (2.7) | 6.6 (4.7) |
|  | Median (IQR) | 1.6 (0.2-1.6) | 6.6 (2.9-9.4) |
|  | Range | 0.0-22.2 | 0.0-22.2 |
| **Total person-years included* (millions)** | | 0.04 | 1.53 |
|  |  |  |  |
| **Cancer stage at diagnosis** | Early | 3343 (13.7) |  |
|  | Late | 8605 (35.2) |  |
|  | Missing | 12482 (51.1) |  |
|  |  |  |  |
| **Age (years)** | 18-39 | 113 ( 0.5) | 1061 ( 0.5) |
|  | 40-59 | 3876 (15.9) | 36788 (15.8) |
|  | 60-79 | 13907 (56.9) | 134233 (57.5) |
|  | >=80 | 6534 (26.7) | 61330 (26.3) |
|  |  |  |  |
| **Sex** | Male | 16337 (66.9) | 155889 (66.8) |
|  | Female | 8093 (33.1) | 77523 (33.2) |
|  |  |  |  |
| **Index of multiple deprivation** | 1 (least deprived) | 4937 (20.2) | 51933 (22.2) |
|  | 2 | 5166 (21.1) | 51786 (22.2) |
|  | 3 | 4875 (20.0) | 46410 (19.9) |
|  | 4 | 4821 (19.7) | 43385 (18.6) |
|  | 5 (most deprived) | 4631 (19.0) | 39898 (17.1) |
|  |  |  |  |
| **Ethnicity** | White | 9904 (40.5) | 120398 (51.6) |
|  | South Asian | 210 ( 0.9) | 3914 ( 1.7) |
|  | Black | 120 ( 0.5) | 2509 ( 1.1) |
|  | Other | 40 ( 0.2) | 868 ( 0.4) |
|  | Mixed | 18 ( 0.1) | 479 ( 0.2) |
|  | Missing | 14138 (57.9) | 105244 (45.1) |
|  |  |  |  |
| **Calendar year** | 1995-2000 | 1340 ( 5.5) | 11859 ( 5.1) |
|  | 2001-2005 | 5190 (21.2) | 48464 (20.8) |
|  | 2006-2010 | 6767 (27.7) | 65049 (27.9) |
|  | 2011-2015 | 7053 (28.9) | 68438 (29.3) |
|  | 2016-2018 | 4080 (16.7) | 39602 (17.0) |
|  |  |  |  |
| **Consultations in year prior to index date** | 0 | 1093 ( 4.5) | 27420 (11.7) |
|  | 1 to 3 | 2082 ( 8.5) | 42572 (18.2) |
|  | 4 to 9 | 6787 (27.8) | 71200 (30.5) |
|  | 10+ | 14468 (59.2) | 92218 (39.5) |
|  |  |  |  |
| **Smoking status** | Non-smoker | 5630 (23.0) | 72535 (31.1) |
|  | Current smoker | 5171 (21.2) | 41858 (17.9) |
|  | Ex-smoker | 13629 (55.8) | 119019 (51.0) |
|  |  |  |  |
| **Problematic alcohol use** | No | 23184 (94.9) | 226543 (97.1) |
|  | Yes | 1246 ( 5.1) | 6869 ( 2.9) |
|  |  |  |  |
| **BMI category** | Underweight | 1299 ( 5.3) | 3729 ( 1.6) |
|  | Normal weight | 8502 (34.8) | 69688 (29.9) |
|  | Overweight | 7862 (32.2) | 84995 (36.4) |
|  | Obese | 4673 (19.1) | 48306 (20.7) |
|  | Missing | 2094 ( 8.6) | 26694 (11.4) |
|  |  |  |  |
| **History of mental illness** | Anxiety | 5394 (22.1) | 47356 (20.3) |
|  | Depression | 6615 (27.1) | 57862 (24.8) |
|  | Non-fatal self-harm | 618 ( 2.5) | 4107 ( 1.8) |

### Stomach (C16)

|  |  | **Cancer survivors** | **Non-cancer comparators** |
| --- | --- | --- | --- |
| **Total** |  | 21364 (100.0) | 202674 (100.0) |
| **Person-years from cancer diagnosis/baseline to end of follow-up** | |  |  |
|  | Mean (SD) | 1.8 (3.1) | 6.8 (5.0) |
|  | Median (IQR) | 1.8 (0.2-1.8) | 6.8 (2.8-9.8) |
|  | Range | 0.0-22.1 | 0.0-22.2 |
| **Total person-years included* (millions)** | | 0.04 | 1.37 |
|  |  |  |  |
| **Cancer stage at diagnosis** | Early | 3491 (16.3) |  |
|  | Late | 5561 (26.0) |  |
|  | Missing | 12312 (57.6) |  |
|  |  |  |  |
| **Age (years)** | 18-39 | 276 ( 1.3) | 2569 ( 1.3) |
|  | 40-59 | 2794 (13.1) | 26528 (13.1) |
|  | 60-79 | 11563 (54.1) | 111154 (54.8) |
|  | >=80 | 6731 (31.5) | 62423 (30.8) |
|  |  |  |  |
| **Sex** | Male | 13967 (65.4) | 132215 (65.2) |
|  | Female | 7397 (34.6) | 70459 (34.8) |
|  |  |  |  |
| **Index of multiple deprivation** | 1 (least deprived) | 3809 (17.8) | 40439 (20.0) |
|  | 2 | 4268 (20.0) | 42198 (20.8) |
|  | 3 | 4066 (19.0) | 40095 (19.8) |
|  | 4 | 4511 (21.1) | 39613 (19.5) |
|  | 5 (most deprived) | 4710 (22.0) | 40329 (19.9) |
|  |  |  |  |
| **Ethnicity** | White | 7437 (34.8) | 98562 (48.6) |
|  | South Asian | 291 ( 1.4) | 4165 ( 2.1) |
|  | Black | 356 ( 1.7) | 2930 ( 1.4) |
|  | Other | 90 ( 0.4) | 838 ( 0.4) |
|  | Mixed | 64 ( 0.3) | 518 ( 0.3) |
|  | Missing | 13126 (61.4) | 95661 (47.2) |
|  |  |  |  |
| **Calendar year** | 1998-2000 | 1779 ( 8.3) | 15713 ( 7.8) |
|  | 2001-2005 | 5567 (26.1) | 51635 (25.5) |
|  | 2006-2010 | 6144 (28.8) | 58987 (29.1) |
|  | 2011-2015 | 5233 (24.5) | 50690 (25.0) |
|  | 2016-2018 | 2641 (12.4) | 25649 (12.7) |
|  |  |  |  |
| **Consultations in year prior to index date** | 0 | 1131 ( 5.3) | 24973 (12.3) |
|  | 1 to 3 | 1483 ( 6.9) | 36106 (17.8) |
|  | 4 to 9 | 5279 (24.7) | 60813 (30.0) |
|  | 10+ | 13471 (63.1) | 80781 (39.9) |
|  |  |  |  |
| **Smoking status** | Non-smoker | 5635 (26.4) | 63148 (31.2) |
|  | Current smoker | 4129 (19.3) | 36298 (17.9) |
|  | Ex-smoker | 11600 (54.3) | 103228 (50.9) |
|  |  |  |  |
| **Problematic alcohol use** | No | 20813 (97.4) | 197281 (97.3) |
|  | Yes | 551 ( 2.6) | 5393 ( 2.7) |
|  |  |  |  |
| **BMI category** | Underweight | 859 ( 4.0) | 3484 ( 1.7) |
|  | Normal weight | 7587 (35.5) | 62335 (30.8) |
|  | Overweight | 7179 (33.6) | 72600 (35.8) |
|  | Obese | 3805 (17.8) | 39565 (19.5) |
|  | Missing | 1934 ( 9.1) | 24690 (12.2) |
|  |  |  |  |
| **History of mental illness** | Anxiety | 4261 (19.9) | 38829 (19.2) |
|  | Depression | 5412 (25.3) | 47714 (23.5) |
|  | Non-fatal self-harm | 408 ( 1.9) | 3286 ( 1.6) |

### Colorectal (C18-20)

|  |  | **Cancer survivors** | **Non-cancer comparators** |
| --- | --- | --- | --- |
| **Total** |  | 117988 (100.0) | 1123544 (100.0) |
| **Person-years from cancer diagnosis/baseline to end of follow-up** | |  |  |
|  | Mean (SD) | 4.3 (4.6) | 6.7 (4.8) |
|  | Median (IQR) | 4.3 (0.6-6.4) | 6.7 (2.9-9.7) |
|  | Range | 0.0-22.2 | 0.0-22.2 |
| **Total person-years included* (millions)** | | 0.5 | 7.55 |
|  |  |  |  |
| **Cancer stage at diagnosis** | Early | 44809 (38.0) |  |
|  | Late | 48870 (41.4) |  |
|  | Missing | 24309 (20.6) |  |
|  |  |  |  |
| **Age (years)** | 18-39 | 1832 ( 1.6) | 17348 ( 1.5) |
|  | 40-59 | 18465 (15.6) | 176480 (15.7) |
|  | 60-79 | 65571 (55.6) | 633349 (56.4) |
|  | >=80 | 32120 (27.2) | 296367 (26.4) |
|  |  |  |  |
| **Sex** | Male | 64464 (54.6) | 610909 (54.4) |
|  | Female | 53524 (45.4) | 512635 (45.6) |
|  |  |  |  |
| **Index of multiple deprivation** | 1 (least deprived) | 26940 (22.8) | 261400 (23.3) |
|  | 2 | 26045 (22.1) | 253496 (22.6) |
|  | 3 | 23765 (20.1) | 224930 (20.0) |
|  | 4 | 21765 (18.4) | 203093 (18.1) |
|  | 5 (most deprived) | 19473 (16.5) | 180625 (16.1) |
|  |  |  |  |
| **Ethnicity** | White | 45469 (38.5) | 561092 (49.9) |
|  | South Asian | 1140 ( 1.0) | 20636 ( 1.8) |
|  | Black | 1068 ( 0.9) | 13467 ( 1.2) |
|  | Other | 326 ( 0.3) | 4782 ( 0.4) |
|  | Mixed | 216 ( 0.2) | 2789 ( 0.2) |
|  | Missing | 69769 (59.1) | 520778 (46.4) |
|  |  |  |  |
| **Calendar year** | 1998-2000 | 7960 ( 6.7) | 70709 ( 6.3) |
|  | 2001-2005 | 25685 (21.8) | 239850 (21.3) |
|  | 2006-2010 | 32250 (27.3) | 310144 (27.6) |
|  | 2011-2015 | 33022 (28.0) | 318805 (28.4) |
|  | 2016-2018 | 19071 (16.2) | 184036 (16.4) |
|  |  |  |  |
| **Consultations in year prior to index date** | 0 | 6063 ( 5.1) | 133615 (11.9) |
|  | 1 to 3 | 10383 ( 8.8) | 204264 (18.2) |
|  | 4 to 9 | 32394 (27.5) | 340382 (30.3) |
|  | 10+ | 69148 (58.6) | 445272 (39.6) |
|  |  |  |  |
| **Smoking status** | Non-smoker | 37587 (31.9) | 372394 (33.1) |
|  | Current smoker | 16755 (14.2) | 194980 (17.4) |
|  | Ex-smoker | 63646 (53.9) | 556170 (49.5) |
|  |  |  |  |
| **Problematic alcohol use** | No | 114453 (97.0) | 1094091 (97.4) |
|  | Yes | 3535 ( 3.0) | 29453 ( 2.6) |
|  |  |  |  |
| **BMI category** | Underweight | 2829 ( 2.4) | 19713 ( 1.8) |
|  | Normal weight | 39627 (33.6) | 351042 (31.2) |
|  | Overweight | 42340 (35.9) | 396508 (35.3) |
|  | Obese | 25102 (21.3) | 231624 (20.6) |
|  | Missing | 8090 ( 6.9) | 124657 (11.1) |
|  |  |  |  |
| **History of mental illness** | Anxiety | 24902 (21.1) | 237903 (21.2) |
|  | Depression | 30291 (25.7) | 289464 (25.8) |
|  | Non-fatal self-harm | 2214 ( 1.9) | 21325 ( 1.9) |

### Liver (C22)

|  |  | **Cancer survivors** | **Non-cancer comparators** |
| --- | --- | --- | --- |
| **Total** |  | 11671 (100.0) | 111740 (100.0) |
| **Person-years from cancer diagnosis/baseline to end of follow-up** | |  |  |
|  | Mean (SD) | 1.1 (2.0) | 6.0 (4.3) |
|  | Median (IQR) | 1.1 (0.1-1.2) | 6.0 (2.7-8.3) |
|  | Range | 0.0-21.9 | 0.0-22.2 |
| **Total person-years included* (millions)** | | 0.01 | 0.67 |
|  |  |  |  |
| **Cancer stage at diagnosis** | Early | 894 ( 7.7) |  |
|  | Late | 2373 (20.3) |  |
|  | Missing | 8404 (72.0) |  |
|  |  |  |  |
| **Age (years)** | 18-39 | 156 ( 1.3) | 1443 ( 1.3) |
|  | 40-59 | 1923 (16.5) | 18209 (16.3) |
|  | 60-79 | 6462 (55.4) | 62428 (55.9) |
|  | >=80 | 3130 (26.8) | 29660 (26.5) |
|  |  |  |  |
| **Sex** | Male | 7543 (64.6) | 71934 (64.4) |
|  | Female | 4128 (35.4) | 39806 (35.6) |
|  |  |  |  |
| **Index of multiple deprivation** | 1 (least deprived) | 2174 (18.6) | 23004 (20.6) |
|  | 2 | 2304 (19.7) | 23497 (21.0) |
|  | 3 | 2266 (19.4) | 22131 (19.8) |
|  | 4 | 2410 (20.6) | 22065 (19.7) |
|  | 5 (most deprived) | 2517 (21.6) | 21043 (18.8) |
|  |  |  |  |
| **Ethnicity** | White | 5504 (47.2) | 61768 (55.3) |
|  | South Asian | 362 ( 3.1) | 3521 ( 3.2) |
|  | Black | 254 ( 2.2) | 2155 ( 1.9) |
|  | Other | 103 ( 0.9) | 678 ( 0.6) |
|  | Mixed | 42 ( 0.4) | 376 ( 0.3) |
|  | Missing | 5406 (46.3) | 43242 (38.7) |
|  |  |  |  |
| **Calendar year** | 1998-2000 | 378 ( 3.2) | 3359 ( 3.0) |
|  | 2001-2005 | 1646 (14.1) | 15250 (13.6) |
|  | 2006-2010 | 2967 (25.4) | 28380 (25.4) |
|  | 2011-2015 | 4043 (34.6) | 39192 (35.1) |
|  | 2016-2018 | 2637 (22.6) | 25559 (22.9) |
|  |  |  |  |
| **Consultations in year prior to index date** | 0 | 428 ( 3.7) | 11979 (10.7) |
|  | 1 to 3 | 531 ( 4.5) | 19594 (17.5) |
|  | 4 to 9 | 2059 (17.6) | 34170 (30.6) |
|  | 10+ | 8653 (74.1) | 45997 (41.2) |
|  |  |  |  |
| **Smoking status** | Non-smoker | 2729 (23.4) | 34518 (30.9) |
|  | Current smoker | 2031 (17.4) | 20163 (18.0) |
|  | Ex-smoker | 6911 (59.2) | 57059 (51.1) |
|  |  |  |  |
| **Problematic alcohol use** | No | 10515 (90.1) | 108179 (96.8) |
|  | Yes | 1156 ( 9.9) | 3561 ( 3.2) |
|  |  |  |  |
| **BMI category** | Underweight | 266 ( 2.3) | 1849 ( 1.7) |
|  | Normal weight | 3434 (29.4) | 33667 (30.1) |
|  | Overweight | 4021 (34.5) | 40360 (36.1) |
|  | Obese | 3186 (27.3) | 24093 (21.6) |
|  | Missing | 764 ( 6.5) | 11771 (10.5) |
|  |  |  |  |
| **History of mental illness** | Anxiety | 3103 (26.6) | 24633 (22.0) |
|  | Depression | 3961 (33.9) | 30248 (27.1) |
|  | Non-fatal self-harm | 361 ( 3.1) | 2185 ( 2.0) |

### Pancreas (C25)

|  |  | **Cancer survivors** | **Non-cancer comparators** |
| --- | --- | --- | --- |
| **Total** |  | 24224 (100.0) | 231873 (100.0) |
| **Person-years from cancer diagnosis/baseline to end of follow-up** | |  |  |
|  | Mean (SD) | 0.7 (1.6) | 6.3 (4.6) |
|  | Median (IQR) | 0.7 (0.1-0.7) | 6.3 (2.8-9.0) |
|  | Range | 0.0-22.2 | 0.0-22.2 |
| **Total person-years included* (millions)** | | 0.02 | 1.47 |
|  |  |  |  |
| **Cancer stage at diagnosis** | Early | 2353 ( 9.7) |  |
|  | Late | 8542 (35.3) |  |
|  | Missing | 13329 (55.0) |  |
|  |  |  |  |
| **Age (years)** | 18-39 | 158 ( 0.7) | 1494 ( 0.6) |
|  | 40-59 | 3452 (14.3) | 33012 (14.2) |
|  | 60-79 | 13491 (55.7) | 130490 (56.3) |
|  | >=80 | 7123 (29.4) | 66877 (28.8) |
|  |  |  |  |
| **Sex** | Male | 12090 (49.9) | 115309 (49.7) |
|  | Female | 12134 (50.1) | 116564 (50.3) |
|  |  |  |  |
| **Index of multiple deprivation** | 1 (least deprived) | 5378 (22.2) | 54090 (23.3) |
|  | 2 | 5383 (22.2) | 51914 (22.4) |
|  | 3 | 4838 (20.0) | 47367 (20.4) |
|  | 4 | 4606 (19.0) | 42305 (18.2) |
|  | 5 (most deprived) | 4019 (16.6) | 36197 (15.6) |
|  |  |  |  |
| **Ethnicity** | White | 10157 (41.9) | 120256 (51.9) |
|  | South Asian | 335 ( 1.4) | 4567 ( 2.0) |
|  | Black | 352 ( 1.5) | 3281 ( 1.4) |
|  | Other | 89 ( 0.4) | 1013 ( 0.4) |
|  | Mixed | 63 ( 0.3) | 594 ( 0.3) |
|  | Missing | 13228 (54.6) | 102162 (44.1) |
|  |  |  |  |
| **Calendar year** | 1998-2000 | 1212 ( 5.0) | 10818 ( 4.7) |
|  | 2001-2005 | 4548 (18.8) | 42449 (18.3) |
|  | 2006-2010 | 6590 (27.2) | 63329 (27.3) |
|  | 2011-2015 | 7337 (30.3) | 71223 (30.7) |
|  | 2016-2018 | 4537 (18.7) | 44054 (19.0) |
|  |  |  |  |
| **Consultations in year prior to index date** | 0 | 909 ( 3.8) | 24193 (10.4) |
|  | 1 to 3 | 979 ( 4.0) | 40618 (17.5) |
|  | 4 to 9 | 4135 (17.1) | 70514 (30.4) |
|  | 10+ | 18201 (75.1) | 96543 (41.6) |
|  |  |  |  |
| **Smoking status** | Non-smoker | 6916 (28.6) | 78744 (34.0) |
|  | Current smoker | 4348 (17.9) | 38725 (16.7) |
|  | Ex-smoker | 12960 (53.5) | 114404 (49.3) |
|  |  |  |  |
| **Problematic alcohol use** | No | 23496 (97.0) | 226064 (97.5) |
|  | Yes | 728 ( 3.0) | 5809 ( 2.5) |
|  |  |  |  |
| **BMI category** | Underweight | 993 ( 4.1) | 4226 ( 1.8) |
|  | Normal weight | 9059 (37.4) | 72288 (31.2) |
|  | Overweight | 7911 (32.7) | 80357 (34.7) |
|  | Obese | 4331 (17.9) | 48394 (20.9) |
|  | Missing | 1930 ( 8.0) | 26608 (11.5) |
|  |  |  |  |
| **History of mental illness** | Anxiety | 5954 (24.6) | 51803 (22.3) |
|  | Depression | 7602 (31.4) | 63501 (27.4) |
|  | Non-fatal self-harm | 621 ( 2.6) | 4409 ( 1.9) |

### Lung (C34)

|  |  | **Cancer survivors** | **Non-cancer comparators** |
| --- | --- | --- | --- |
| **Total** |  | 113178 (100.0) | 1073350 (100.0) |
| **Person-years from cancer diagnosis/baseline to end of follow-up** | |  |  |
|  | Mean (SD) | 1.2 (2.2) | 6.6 (4.8) |
|  | Median (IQR) | 1.2 (0.1-1.3) | 6.6 (2.9-9.5) |
|  | Range | 0.0-22.2 | 0.0-22.2 |
| **Total person-years included* (millions)** | | 0.14 | 7.13 |
|  |  |  |  |
| **Cancer stage at diagnosis** | Early | 14592 (12.9) |  |
|  | Late | 52069 (46.0) |  |
|  | Missing | 46517 (41.1) |  |
|  |  |  |  |
| **Age (years)** | 18-39 | 436 ( 0.4) | 4099 ( 0.4) |
|  | 40-59 | 14039 (12.4) | 133830 (12.5) |
|  | 60-79 | 69679 (61.6) | 672663 (62.7) |
|  | >=80 | 29024 (25.6) | 262758 (24.5) |
|  |  |  |  |
| **Sex** | Male | 63070 (55.7) | 591830 (55.1) |
|  | Female | 50108 (44.3) | 481520 (44.9) |
|  |  |  |  |
| **Index of multiple deprivation** | 1 (least deprived) | 17785 (15.7) | 210158 (19.6) |
|  | 2 | 20439 (18.1) | 222086 (20.7) |
|  | 3 | 21447 (18.9) | 208741 (19.4) |
|  | 4 | 24381 (21.5) | 212285 (19.8) |
|  | 5 (most deprived) | 29126 (25.7) | 220080 (20.5) |
|  |  |  |  |
| **Ethnicity** | White | 48526 (42.9) | 563025 (52.5) |
|  | South Asian | 957 ( 0.8) | 21391 ( 2.0) |
|  | Black | 754 ( 0.7) | 15225 ( 1.4) |
|  | Other | 290 ( 0.3) | 4815 ( 0.4) |
|  | Mixed | 194 ( 0.2) | 2896 ( 0.3) |
|  | Missing | 62457 (55.2) | 465998 (43.4) |
|  |  |  |  |
| **Calendar year** | 1998-2000 | 6738 ( 6.0) | 60323 ( 5.6) |
|  | 2001-2005 | 23525 (20.8) | 220111 (20.5) |
|  | 2006-2010 | 31267 (27.6) | 299139 (27.9) |
|  | 2011-2015 | 32784 (29.0) | 313671 (29.2) |
|  | 2016-2018 | 18864 (16.7) | 180106 (16.8) |
|  |  |  |  |
| **Consultations in year prior to index date** | 0 | 5066 ( 4.5) | 116017 (10.8) |
|  | 1 to 3 | 5790 ( 5.1) | 186321 (17.4) |
|  | 4 to 9 | 23344 (20.6) | 326815 (30.4) |
|  | 10+ | 78978 (69.8) | 444186 (41.4) |
|  |  |  |  |
| **Smoking status** | Non-smoker | 8907 ( 7.9) | 331360 (30.9) |
|  | Current smoker | 35693 (31.5) | 194962 (18.2) |
|  | Ex-smoker | 68578 (60.6) | 547028 (51.0) |
|  |  |  |  |
| **Problematic alcohol use** | No | 108197 (95.6) | 1044165 (97.3) |
|  | Yes | 4981 ( 4.4) | 29185 ( 2.7) |
|  |  |  |  |
| **BMI category** | Underweight | 6414 ( 5.7) | 18639 ( 1.7) |
|  | Normal weight | 44043 (38.9) | 327441 (30.5) |
|  | Overweight | 34120 (30.1) | 382478 (35.6) |
|  | Obese | 18477 (16.3) | 232943 (21.7) |
|  | Missing | 10124 ( 8.9) | 111849 (10.4) |
|  |  |  |  |
| **History of mental illness** | Anxiety | 31086 (27.5) | 233816 (21.8) |
|  | Depression | 38533 (34.0) | 285429 (26.6) |
|  | Non-fatal self-harm | 4319 ( 3.8) | 21532 ( 2.0) |

### Malignant melanoma (C43)

|  |  | **Cancer survivors** | **Non-cancer comparators** |
| --- | --- | --- | --- |
| **Total** |  | 37587 (100.0) | 361204 (100.0) |
| **Person-years from cancer diagnosis/baseline to end of follow-up** | |  |  |
|  | Mean (SD) | 6.4 (5.0) | 7.0 (5.0) |
|  | Median (IQR) | 6.4 (2.6-9.2) | 7.0 (3.1-10.1) |
|  | Range | 0.0-22.2 | 0.0-22.2 |
| **Total person-years included* (millions)** | | 0.24 | 2.54 |
|  |  |  |  |
| **Cancer stage at diagnosis** | Early | 17480 (46.5) |  |
|  | Late | 2372 ( 6.3) |  |
|  | Missing | 17735 (47.2) |  |
|  |  |  |  |
| **Age (years)** | 18-39 | 4340 (11.5) | 41151 (11.4) |
|  | 40-59 | 12056 (32.1) | 115829 (32.1) |
|  | 60-79 | 15512 (41.3) | 150605 (41.7) |
|  | >=80 | 5679 (15.1) | 53619 (14.8) |
|  |  |  |  |
| **Sex** | Male | 17686 (47.1) | 168678 (46.7) |
|  | Female | 19901 (52.9) | 192526 (53.3) |
|  |  |  |  |
| **Index of multiple deprivation** | 1 (least deprived) | 10917 (29.0) | 95853 (26.5) |
|  | 2 | 9300 (24.7) | 86188 (23.9) |
|  | 3 | 7590 (20.2) | 72402 (20.0) |
|  | 4 | 5854 (15.6) | 60051 (16.6) |
|  | 5 (most deprived) | 3926 (10.4) | 46710 (12.9) |
|  |  |  |  |
| **Ethnicity** | White | 16047 (42.7) | 179231 (49.6) |
|  | South Asian | 27 ( 0.1) | 5591 ( 1.5) |
|  | Black | 30 ( 0.1) | 3197 ( 0.9) |
|  | Other | 37 ( 0.1) | 1740 ( 0.5) |
|  | Mixed | 14 ( 0.0) | 1032 ( 0.3) |
|  | Missing | 21432 (57.0) | 170413 (47.2) |
|  |  |  |  |
| **Calendar year** | 1998-2000 | 1846 ( 4.9) | 16721 ( 4.6) |
|  | 2001-2005 | 6768 (18.0) | 63647 (17.6) |
|  | 2006-2010 | 9945 (26.5) | 95897 (26.5) |
|  | 2011-2015 | 11572 (30.8) | 112403 (31.1) |
|  | 2016-2018 | 7456 (19.8) | 72536 (20.1) |
|  |  |  |  |
| **Consultations in year prior to index date** | 0 | 1900 ( 5.1) | 53999 (14.9) |
|  | 1 to 3 | 6849 (18.2) | 79880 (22.1) |
|  | 4 to 9 | 13305 (35.4) | 109158 (30.2) |
|  | 10+ | 15533 (41.3) | 118164 (32.7) |
|  |  |  |  |
| **Smoking status** | Non-smoker | 14950 (39.8) | 128649 (35.6) |
|  | Current smoker | 4582 (12.2) | 65340 (18.1) |
|  | Ex-smoker | 18055 (48.0) | 167215 (46.3) |
|  |  |  |  |
| **Problematic alcohol use** | No | 36591 (97.4) | 350662 (97.1) |
|  | Yes | 996 ( 2.6) | 10542 ( 2.9) |
|  |  |  |  |
| **BMI category** | Underweight | 498 ( 1.3) | 6100 ( 1.7) |
|  | Normal weight | 12949 (34.5) | 122261 (33.8) |
|  | Overweight | 13819 (36.8) | 121011 (33.5) |
|  | Obese | 8187 (21.8) | 76056 (21.1) |
|  |  |  |  |
| **History of mental illness** | Anxiety | 8306 (22.1) | 85036 (23.5) |
|  | Depression | 9667 (25.7) | 99660 (27.6) |
|  | Non-fatal self-harm | 810 ( 2.2) | 9774 ( 2.7) |

### Breast (C50)

|  |  | **Cancer survivors** | **Non-cancer comparators** |
| --- | --- | --- | --- |
| **Total** |  | 152129 (100.0) | 1465725 (100.0) |
| **Person-years from cancer diagnosis/baseline to end of follow-up** | |  |  |
|  | Mean (SD) | 6.8 (5.2) | 7.7 (5.4) |
|  | Median (IQR) | 6.8 (2.7-9.9) | 7.7 (3.4-11.2) |
|  | Range | 0.0-22.2 | 0.0-22.2 |
| **Total person-years included* (millions)** | | 1.03 | 11.28 |
|  |  |  |  |
| **Cancer stage at diagnosis** | Early | 90465 (59.5) |  |
|  | Late | 16522 (10.9) |  |
|  | Missing | 45142 (29.7) |  |
|  |  |  |  |
| **Age (years)** | 18-39 | 6600 ( 4.3) | 63824 ( 4.4) |
|  | 40-59 | 58584 (38.5) | 568554 (38.8) |
|  | 60-79 | 63966 (42.0) | 620795 (42.4) |
|  | >=80 | 22979 (15.1) | 212552 (14.5) |
|  |  |  |  |
| **Sex** | Male | 0 ( 0.0) | 0 ( 0.0) |
|  | Female | 152129 (100.0) | 1465725 (100.0) |
|  |  |  |  |
| **Index of multiple deprivation** | 1 (least deprived) | 37609 (24.7) | 356585 (24.3) |
|  | 2 | 34899 (22.9) | 333278 (22.7) |
|  | 3 | 30282 (19.9) | 292260 (19.9) |
|  | 4 | 26935 (17.7) | 260870 (17.8) |
|  | 5 (most deprived) | 22404 (14.7) | 222732 (15.2) |
|  |  |  |  |
| **Ethnicity** | White | 55612 (36.6) | 678107 (46.3) |
|  | South Asian | 2418 ( 1.6) | 32577 ( 2.2) |
|  | Black | 1764 ( 1.2) | 22526 ( 1.5) |
|  | Other | 608 ( 0.4) | 8487 ( 0.6) |
|  | Mixed | 395 ( 0.3) | 5058 ( 0.3) |
|  | Missing | 91332 (60.0) | 718970 (49.1) |
|  |  |  |  |
| **Calendar year** | 1998-2000 | 10855 ( 7.1) | 99833 ( 6.8) |
|  | 2001-2005 | 34546 (22.7) | 328128 (22.4) |
|  | 2006-2010 | 39869 (26.2) | 387324 (26.4) |
|  | 2011-2015 | 42089 (27.7) | 409740 (28.0) |
|  | 2016-2018 | 24770 (16.3) | 240700 (16.4) |
|  |  |  |  |
| **Consultations in year prior to index date** | 0 | 10261 ( 6.7) | 179058 (12.2) |
|  | 1 to 3 | 26896 (17.7) | 309898 (21.1) |
|  | 4 to 9 | 52557 (34.5) | 463845 (31.6) |
|  | 10+ | 62414 (41.0) | 512919 (35.0) |
|  |  |  |  |
| **Smoking status** | Non-smoker | 62283 (40.9) | 588719 (40.2) |
|  | Current smoker | 22239 (14.6) | 251764 (17.2) |
|  | Ex-smoker | 67607 (44.4) | 625242 (42.7) |
|  |  |  |  |
| **Problematic alcohol use** | No | 149064 (98.0) | 1436918 (98.0) |
|  | Yes | 3065 ( 2.0) | 28807 ( 2.0) |
|  |  |  |  |
| **BMI category** | Underweight | 2794 ( 1.8) | 32030 ( 2.2) |
|  | Normal weight | 55592 (36.5) | 539887 (36.8) |
|  | Overweight | 47931 (31.5) | 439221 (30.0) |
|  | Obese | 37538 (24.7) | 339749 (23.2) |
|  | Missing | 8274 ( 5.4) | 114838 ( 7.8) |
|  |  |  |  |
| **History of mental illness** | Anxiety | 43315 (28.5) | 410162 (28.0) |
|  | Depression | 50321 (33.1) | 480898 (32.8) |
|  | Non-fatal self-harm | 4433 ( 2.9) | 45812 ( 3.1) |

### Cervix (C53)

|  |  | **Cancer survivors** | **Non-cancer comparators** |
| --- | --- | --- | --- |
| **Total** |  | 8838 (100.0) | 85280 (100.0) |
| **Person-years from cancer diagnosis/baseline to end of follow-up** | |  |  |
|  | Mean (SD) | 6.1 (5.6) | 7.9 (5.6) |
|  | Median (IQR) | 6.1 (1.5-9.2) | 7.9 (3.3-11.7) |
|  | Range | 0.0-22.2 | 0.0-22.2 |
| **Total person-years included* (millions)** | | 0.05 | 0.67 |
|  |  |  |  |
| **Cancer stage at diagnosis** | Early | 5955 (67.4) |  |
|  | Late | 1200 (13.6) |  |
|  | Missing | 1683 (19.0) |  |
|  |  |  |  |
| **Age (years)** | 18-39 | 3468 (39.2) | 33452 (39.2) |
|  | 40-59 | 3042 (34.4) | 29535 (34.6) |
|  | 60-79 | 1660 (18.8) | 16038 (18.8) |
|  | >=80 | 668 ( 7.6) | 6255 ( 7.3) |
|  |  |  |  |
| **Sex** | Male | 0 ( 0.0) | 0 ( 0.0) |
|  | Female | 8838 (100.0) | 85280 (100.0) |
|  |  |  |  |
| **Index of multiple deprivation** | 1 (least deprived) | 1548 (17.5) | 16317 (19.1) |
|  | 2 | 1597 (18.1) | 16863 (19.8) |
|  | 3 | 1660 (18.8) | 16613 (19.5) |
|  | 4 | 1848 (20.9) | 17349 (20.3) |
|  | 5 (most deprived) | 2185 (24.7) | 18138 (21.3) |
|  |  |  |  |
| **Ethnicity** | White | 3326 (37.6) | 37825 (44.4) |
|  | South Asian | 95 ( 1.1) | 2422 ( 2.8) |
|  | Black | 93 ( 1.1) | 1683 ( 2.0) |
|  | Other | 66 ( 0.7) | 685 ( 0.8) |
|  | Mixed | 35 ( 0.4) | 485 ( 0.6) |
|  | Missing | 5223 (59.1) | 42180 (49.5) |
|  |  |  |  |
| **Calendar year** | 1998-2000 | 684 ( 7.7) | 6342 ( 7.4) |
|  | 2001-2005 | 1956 (22.1) | 18586 (21.8) |
|  | 2006-2010 | 2405 (27.2) | 23367 (27.4) |
|  | 2011-2015 | 2382 (27.0) | 23211 (27.2) |
|  | 2016-2018 | 1411 (16.0) | 13774 (16.2) |
|  |  |  |  |
| **Consultations in year prior to index date** | 0 | 550 ( 6.2) | 12809 (15.0) |
|  | 1 to 3 | 1242 (14.1) | 20316 (23.8) |
|  | 4 to 9 | 3228 (36.5) | 27391 (32.1) |
|  | 10+ | 3818 (43.2) | 24762 (29.0) |
|  |  |  |  |
| **Smoking status** | Non-smoker | 2857 (32.3) | 33572 (39.4) |
|  | Current smoker | 2154 (24.4) | 17056 (20.0) |
|  | Ex-smoker | 3827 (43.3) | 34652 (40.6) |
|  |  |  |  |
| **Problematic alcohol use** | No | 8575 (97.0) | 83243 (97.6) |
|  | Yes | 263 ( 3.0) | 2037 ( 2.4) |
|  |  |  |  |
| **BMI category** | Underweight | 285 ( 3.2) | 2204 ( 2.6) |
|  | Normal weight | 3502 (39.6) | 34886 (40.9) |
|  | Overweight | 2256 (25.5) | 22989 (27.0) |
|  | Obese | 2028 (22.9) | 18744 (22.0) |
|  | Missing | 767 ( 8.7) | 6457 ( 7.6) |
|  |  |  |  |
| **History of mental illness** | Anxiety | 2640 (29.9) | 23738 (27.8) |
|  | Depression | 2940 (33.3) | 26974 (31.6) |
|  | Non-fatal self-harm | 491 ( 5.6) | 3606 ( 4.2) |

### Uterus (C54-55)

|  |  | **Cancer survivors** | **Non-cancer comparators** |
| --- | --- | --- | --- |
| **Total** |  | 23696 (100.0) | 229369 (100.0) |
| **Person-years from cancer diagnosis/baseline to end of follow-up** | |  |  |
|  | Mean (SD) | 6.1 (5.2) | 7.4 (5.1) |
|  | Median (IQR) | 6.1 (2.0-9.2) | 7.4 (3.3-10.6) |
|  | Range | 0.0-22.2 | 0.0-22.2 |
| **Total person-years included* (millions)** | | 0.14 | 1.7 |
|  |  |  |  |
| **Cancer stage at diagnosis** | Early | 14287 (60.3) |  |
|  | Late | 3302 (13.9) |  |
|  | Missing | 6107 (25.8) |  |
|  |  |  |  |
| **Age (years)** | 18-39 | 297 ( 1.3) | 2875 ( 1.3) |
|  | 40-59 | 6158 (26.0) | 59787 (26.1) |
|  | 60-79 | 13816 (58.3) | 134312 (58.6) |
|  | >=80 | 3425 (14.5) | 32395 (14.1) |
|  |  |  |  |
| **Sex** | Male | 0 ( 0.0) | 0 ( 0.0) |
|  | Female | 23696 (100.0) | 229369 (100.0) |
|  |  |  |  |
| **Index of multiple deprivation** | 1 (least deprived) | 5192 (21.9) | 51770 (22.6) |
|  | 2 | 5268 (22.2) | 51134 (22.3) |
|  | 3 | 4930 (20.8) | 46844 (20.4) |
|  | 4 | 4493 (19.0) | 42862 (18.7) |
|  | 5 (most deprived) | 3813 (16.1) | 36759 (16.0) |
|  |  |  |  |
| **Ethnicity** | White | 9107 (38.4) | 116184 (50.7) |
|  | South Asian | 545 ( 2.3) | 5802 ( 2.5) |
|  | Black | 379 ( 1.6) | 4013 ( 1.7) |
|  | Other | 89 ( 0.4) | 1290 ( 0.6) |
|  | Mixed | 59 ( 0.2) | 699 ( 0.3) |
|  | Missing | 13517 (57.0) | 101381 (44.2) |
|  |  |  |  |
| **Calendar year** | 1998-2000 | 1523 ( 6.4) | 14073 ( 6.1) |
|  | 2001-2005 | 4742 (20.0) | 45264 (19.7) |
|  | 2006-2010 | 6282 (26.5) | 61115 (26.6) |
|  | 2011-2015 | 6866 (29.0) | 67083 (29.2) |
|  | 2016-2018 | 4283 (18.1) | 41834 (18.2) |
|  |  |  |  |
| **Consultations in year prior to index date** | 0 | 1132 ( 4.8) | 25116 (11.0) |
|  | 1 to 3 | 2113 ( 8.9) | 44416 (19.4) |
|  | 4 to 9 | 7055 (29.8) | 72621 (31.7) |
|  | 10+ | 13396 (56.5) | 87216 (38.0) |
|  |  |  |  |
| **Smoking status** | Non-smoker | 10651 (44.9) | 89470 (39.0) |
|  | Current smoker | 2517 (10.6) | 38865 (16.9) |
|  | Ex-smoker | 10528 (44.4) | 101034 (44.0) |
|  |  |  |  |
| **Problematic alcohol use** | No | 23373 (98.6) | 225226 (98.2) |
|  | Yes | 323 ( 1.4) | 4143 ( 1.8) |
|  |  |  |  |
| **BMI category** | Underweight | 217 ( 0.9) | 4616 ( 2.0) |
|  | Normal weight | 4823 (20.4) | 77748 (33.9) |
|  | Overweight | 6325 (26.7) | 70590 (30.8) |
|  | Obese | 11216 (47.3) | 55723 (24.3) |
|  | Missing | 1115 ( 4.7) | 20692 ( 9.0) |
|  |  |  |  |
| **History of mental illness** | Anxiety | 6291 (26.5) | 64128 (28.0) |
|  | Depression | 7327 (30.9) | 76206 (33.2) |
|  | Non-fatal self-harm | 521 ( 2.2) | 6489 ( 2.8) |

### Ovary (C56)

|  |  | **Cancer survivors** | **Non-cancer comparators** |
| --- | --- | --- | --- |
| **Total** |  | 20503 (100.0) | 197831 (100.0) |
| **Person-years from cancer diagnosis/baseline to end of follow-up** | |  |  |
|  | Mean (SD) | 4.0 (4.7) | 7.7 (5.3) |
|  | Median (IQR) | 4.0 (0.6-5.6) | 7.7 (3.4-11.1) |
|  | Range | 0.0-22.2 | 0.0-22.2 |
| **Total person-years included* (millions)** | | 0.08 | 1.52 |
|  |  |  |  |
| **Cancer stage at diagnosis** | Early | 4956 (24.2) |  |
|  | Late | 7855 (38.3) |  |
|  | Missing | 7692 (37.5) |  |
|  |  |  |  |
| **Age (years)** | 18-39 | 1404 ( 6.8) | 13540 ( 6.8) |
|  | 40-59 | 5818 (28.4) | 56467 (28.5) |
|  | 60-79 | 9884 (48.2) | 95805 (48.4) |
|  | >=80 | 3397 (16.6) | 32019 (16.2) |
|  |  |  |  |
| **Sex** | Male | 0 ( 0.0) | 0 ( 0.0) |
|  | Female | 20503 (100.0) | 197831 (100.0) |
|  |  |  |  |
| **Index of multiple deprivation** | 1 (least deprived) | 4661 (22.7) | 44962 (22.7) |
|  | 2 | 4551 (22.2) | 44536 (22.5) |
|  | 3 | 4188 (20.4) | 40192 (20.3) |
|  | 4 | 3761 (18.3) | 36598 (18.5) |
|  | 5 (most deprived) | 3342 (16.3) | 31543 (15.9) |
|  |  |  |  |
| **Ethnicity** | White | 7413 (36.2) | 94617 (47.8) |
|  | South Asian | 312 ( 1.5) | 4440 ( 2.2) |
|  | Black | 169 ( 0.8) | 2866 ( 1.4) |
|  | Other | 84 ( 0.4) | 985 ( 0.5) |
|  | Mixed | 51 ( 0.2) | 645 ( 0.3) |
|  | Missing | 12474 (60.8) | 94278 (47.7) |
|  |  |  |  |
| **Calendar year** | 1998-2000 | 1523 ( 7.4) | 13955 ( 7.1) |
|  | 2001-2005 | 4853 (23.7) | 46228 (23.4) |
|  | 2006-2010 | 5582 (27.2) | 54252 (27.4) |
|  | 2011-2015 | 5501 (26.8) | 53714 (27.2) |
|  | 2016-2018 | 3044 (14.8) | 29682 (15.0) |
|  |  |  |  |
| **Consultations in year prior to index date** | 0 | 1091 ( 5.3) | 23495 (11.9) |
|  | 1 to 3 | 1448 ( 7.1) | 39983 (20.2) |
|  | 4 to 9 | 5406 (26.4) | 62416 (31.6) |
|  | 10+ | 12558 (61.2) | 71937 (36.4) |
|  |  |  |  |
| **Smoking status** | Non-smoker | 8467 (41.3) | 78351 (39.6) |
|  | Current smoker | 3152 (15.4) | 34042 (17.2) |
|  | Ex-smoker | 8884 (43.3) | 85438 (43.2) |
|  |  |  |  |
| **Problematic alcohol use** | No | 20204 (98.5) | 194378 (98.3) |
|  | Yes | 299 ( 1.5) | 3453 ( 1.7) |
|  |  |  |  |
| **BMI category** | Underweight | 513 ( 2.5) | 4490 ( 2.3) |
|  | Normal weight | 7301 (35.6) | 70550 (35.7) |
|  | Overweight | 6160 (30.0) | 59398 (30.0) |
|  | Obese | 5003 (24.4) | 45093 (22.8) |
|  | Missing | 1526 ( 7.4) | 18300 ( 9.3) |
|  |  |  |  |
| **History of mental illness** | Anxiety | 5614 (27.4) | 53889 (27.2) |
|  | Depression | 6448 (31.4) | 63492 (32.1) |
|  | Non-fatal self-harm | 579 ( 2.8) | 5879 ( 3.0) |

### Prostate (C61)

|  |  | **Cancer survivors** | **Non-cancer comparators** |
| --- | --- | --- | --- |
| **Total** |  | 132283 (100.0) | 1211868 (100.0) |
| **Person-years from cancer diagnosis/baseline to end of follow-up** | |  |  |
|  | Mean (SD) | 5.9 (4.5) | 6.5 (4.6) |
|  | Median (IQR) | 5.9 (2.4-8.5) | 6.5 (2.9-9.3) |
|  | Range | 0.0-22.2 | 0.0-22.2 |
| **Total person-years included* (millions)** | | 0.77 | 7.91 |
|  |  |  |  |
| **Cancer stage at diagnosis** | Early | 35369 (26.7) |  |
|  | Late | 29056 (22.0) |  |
|  | Missing | 67858 (51.3) |  |
|  |  |  |  |
| **Age (years)** | 18-39 | 24 ( 0.0) | 224 ( 0.0) |
|  | 40-59 | 13530 (10.2) | 128195 (10.6) |
|  | 60-79 | 93452 (70.6) | 893171 (73.7) |
|  | >=80 | 25277 (19.1) | 190278 (15.7) |
|  |  |  |  |
| **Sex** | Male | 132283 (100.0) | 1211868 (100.0) |
|  | Female | 0 ( 0.0) | 0 ( 0.0) |
|  |  |  |  |
| **Index of multiple deprivation** | 1 (least deprived) | 34471 (26.1) | 300198 (24.8) |
|  | 2 | 31021 (23.5) | 279276 (23.0) |
|  | 3 | 26407 (20.0) | 245828 (20.3) |
|  | 4 | 22524 (17.0) | 212595 (17.5) |
|  | 5 (most deprived) | 17860 (13.5) | 173971 (14.4) |
|  |  |  |  |
| **Ethnicity** | White | 52477 (39.7) | 625672 (51.6) |
|  | South Asian | 1365 ( 1.0) | 26292 ( 2.2) |
|  | Black | 3233 ( 2.4) | 17025 ( 1.4) |
|  | Other | 321 ( 0.2) | 5528 ( 0.5) |
|  | Mixed | 404 ( 0.3) | 3077 ( 0.3) |
|  | Missing | 74483 (56.3) | 534274 (44.1) |
|  |  |  |  |
| **Calendar year** | 1998-2000 | 7268 ( 5.5) | 64126 ( 5.3) |
|  | 2001-2005 | 27882 (21.1) | 258040 (21.3) |
|  | 2006-2010 | 34680 (26.2) | 325449 (26.9) |
|  | 2011-2015 | 38068 (28.8) | 346463 (28.6) |
|  | 2016-2018 | 24385 (18.4) | 217790 (18.0) |
|  |  |  |  |
| **Consultations in year prior to index date** | 0 | 6321 ( 4.8) | 149012 (12.3) |
|  | 1 to 3 | 7916 ( 6.0) | 228234 (18.8) |
|  | 4 to 9 | 39206 (29.6) | 375461 (31.0) |
|  | 10+ | 78839 (59.6) | 459150 (37.9) |
|  |  |  |  |
| **Smoking status** | Non-smoker | 38217 (28.9) | 325739 (26.9) |
|  | Current smoker | 17334 (13.1) | 220782 (18.2) |
|  | Ex-smoker | 76732 (58.0) | 665347 (54.9) |
|  |  |  |  |
| **Problematic alcohol use** | No | 127966 (96.7) | 1167966 (96.4) |
|  | Yes | 4317 ( 3.3) | 43902 ( 3.6) |
|  |  |  |  |
| **BMI category** | Underweight | 1145 ( 0.9) | 11141 ( 0.9) |
|  | Normal weight | 40572 (30.7) | 340678 (28.1) |
|  | Overweight | 58820 (44.5) | 489337 (40.4) |
|  | Obese | 25953 (19.6) | 249602 (20.6) |
|  | Missing | 5793 ( 4.4) | 121110 (10.0) |
|  |  |  |  |
| **History of mental illness** | Anxiety | 22290 (16.9) | 197684 (16.3) |
|  | Depression | 26895 (20.3) | 243251 (20.1) |
|  | Non-fatal self-harm | 1658 ( 1.3) | 17692 ( 1.5) |

### Kidney (C64)

|  |  | **Cancer survivors** | **Non-cancer comparators** |
| --- | --- | --- | --- |
| **Total** |  | 24901 (100.0) | 239002 (100.0) |
| **Person-years from cancer diagnosis/baseline to end of follow-up** | |  |  |
|  | Mean (SD) | 4.0 (4.3) | 6.6 (4.7) |
|  | Median (IQR) | 4.0 (0.5-6.1) | 6.6 (3.0-9.4) |
|  | Range | 0.0-22.2 | 0.0-22.2 |
| **Total person-years included* (millions)** | | 0.1 | 1.59 |
|  |  |  |  |
| **Cancer stage at diagnosis** | Early | 5898 (23.7) |  |
|  | Late | 6425 (25.8) |  |
|  | Missing | 12578 (50.5) |  |
|  |  |  |  |
| **Age (years)** | 18-39 | 545 ( 2.2) | 5151 ( 2.2) |
|  | 40-59 | 6012 (24.1) | 57372 (24.0) |
|  | 60-79 | 13478 (54.1) | 130335 (54.5) |
|  | >=80 | 4866 (19.5) | 46144 (19.3) |
|  |  |  |  |
| **Sex** | Male | 15546 (62.4) | 148390 (62.1) |
|  | Female | 9355 (37.6) | 90612 (37.9) |
|  |  |  |  |
| **Index of multiple deprivation** | 1 (least deprived) | 5406 (21.7) | 54447 (22.8) |
|  | 2 | 5420 (21.8) | 53578 (22.4) |
|  | 3 | 4988 (20.0) | 47677 (19.9) |
|  | 4 | 4688 (18.8) | 43521 (18.2) |
|  | 5 (most deprived) | 4399 (17.7) | 39779 (16.6) |
|  |  |  |  |
| **Ethnicity** | White | 10987 (44.1) | 126503 (52.9) |
|  | South Asian | 389 ( 1.6) | 5341 ( 2.2) |
|  | Black | 331 ( 1.3) | 3487 ( 1.5) |
|  | Other | 100 ( 0.4) | 1300 ( 0.5) |
|  | Mixed | 67 ( 0.3) | 722 ( 0.3) |
|  | Missing | 13027 (52.3) | 101649 (42.5) |
|  |  |  |  |
| **Calendar year** | 1998-2000 | 1229 ( 4.9) | 11133 ( 4.7) |
|  | 2001-2005 | 4221 (17.0) | 39538 (16.5) |
|  | 2006-2010 | 6318 (25.4) | 60781 (25.4) |
|  | 2011-2015 | 8061 (32.4) | 78211 (32.7) |
|  | 2016-2018 | 5072 (20.4) | 49339 (20.6) |
|  |  |  |  |
| **Consultations in year prior to index date** | 0 | 1110 ( 4.5) | 31730 (13.3) |
|  | 1 to 3 | 1277 ( 5.1) | 46459 (19.4) |
|  | 4 to 9 | 5054 (20.3) | 72638 (30.4) |
|  | 10+ | 17460 (70.1) | 88172 (36.9) |
|  |  |  |  |
| **Smoking status** | Non-smoker | 6830 (27.4) | 75876 (31.7) |
|  | Current smoker | 4159 (16.7) | 43696 (18.3) |
|  | Ex-smoker | 13912 (55.9) | 119430 (50.0) |
|  |  |  |  |
| **Problematic alcohol use** | No | 24154 (97.0) | 231524 (96.9) |
|  | Yes | 747 ( 3.0) | 7478 ( 3.1) |
|  |  |  |  |
| **BMI category** | Underweight | 422 ( 1.7) | 3530 ( 1.5) |
|  | Normal weight | 7234 (29.1) | 72074 (30.2) |
|  | Overweight | 8984 (36.1) | 86867 (36.3) |
|  | Obese | 6884 (27.6) | 52689 (22.0) |
|  | Missing | 1377 ( 5.5) | 23842 (10.0) |
|  |  |  |  |
| **History of mental illness** | Anxiety | 6391 (25.7) | 52622 (22.0) |
|  | Depression | 7635 (30.7) | 62728 (26.2) |
|  | Non-fatal self-harm | 686 ( 2.8) | 5232 ( 2.2) |

### Bladder (C67)

|  |  | **Cancer survivors** | **Non-cancer comparators** |
| --- | --- | --- | --- |
| **Total** |  | 30911 (100.0) | 293078 (100.0) |
| **Person-years from cancer diagnosis/baseline to end of follow-up** | |  |  |
|  | Mean (SD) | 3.9 (4.6) | 6.5 (4.9) |
|  | Median (IQR) | 3.9 (0.5-5.9) | 6.5 (2.7-9.4) |
|  | Range | 0.0-22.2 | 0.0-22.2 |
| **Total person-years included* (millions)** | | 0.12 | 1.92 |
|  |  |  |  |
| **Cancer stage at diagnosis** | Early | 8629 (27.9) |  |
|  | Late | 3584 (11.6) |  |
|  | Missing | 18698 (60.5) |  |
|  |  |  |  |
| **Age (years)** | 18-39 | 144 ( 0.5) | 1307 ( 0.4) |
|  | 40-59 | 2907 ( 9.4) | 27474 ( 9.4) |
|  | 60-79 | 17373 (56.2) | 167156 (57.0) |
|  | >=80 | 10487 (33.9) | 97141 (33.1) |
|  |  |  |  |
| **Sex** | Male | 22524 (72.9) | 213243 (72.8) |
|  | Female | 8387 (27.1) | 79835 (27.2) |
|  |  |  |  |
| **Index of multiple deprivation** | 1 (least deprived) | 6539 (21.2) | 66275 (22.6) |
|  | 2 | 6813 (22.0) | 65462 (22.3) |
|  | 3 | 5968 (19.3) | 58428 (19.9) |
|  | 4 | 5921 (19.2) | 53537 (18.3) |
|  | 5 (most deprived) | 5670 (18.3) | 49376 (16.8) |
|  |  |  |  |
| **Ethnicity** | White | 11614 (37.6) | 146133 (49.9) |
|  | South Asian | 219 ( 0.7) | 4544 ( 1.6) |
|  | Black | 122 ( 0.4) | 2939 ( 1.0) |
|  | Other | 64 ( 0.2) | 965 ( 0.3) |
|  | Mixed | 33 ( 0.1) | 583 ( 0.2) |
|  | Missing | 18859 (61.0) | 137914 (47.1) |
|  |  |  |  |
| **Calendar year** | 1998-2000 | 2697 ( 8.7) | 23930 ( 8.2) |
|  | 2001-2005 | 7314 (23.7) | 67808 (23.1) |
|  | 2006-2010 | 8656 (28.0) | 82846 (28.3) |
|  | 2011-2015 | 7819 (25.3) | 75553 (25.8) |
|  | 2016-2018 | 4425 (14.3) | 42941 (14.7) |
|  |  |  |  |
| **Consultations in year prior to index date** | 0 | 1626 ( 5.3) | 33475 (11.4) |
|  | 1 to 3 | 2082 ( 6.7) | 50337 (17.2) |
|  | 4 to 9 | 7332 (23.7) | 88531 (30.2) |
|  | 10+ | 19871 (64.3) | 120733 (41.2) |
|  |  |  |  |
| **Smoking status** | Non-smoker | 6905 (22.3) | 88584 (30.2) |
|  | Current smoker | 6120 (19.8) | 50816 (17.3) |
|  | Ex-smoker | 17886 (57.9) | 153678 (52.4) |
|  |  |  |  |
| **Problematic alcohol use** | No | 29990 (97.0) | 285706 (97.5) |
|  | Yes | 921 ( 3.0) | 7372 ( 2.5) |
|  |  |  |  |
| **BMI category** | Underweight | 717 ( 2.3) | 4622 ( 1.6) |
|  | Normal weight | 10122 (32.7) | 89425 (30.5) |
|  | Overweight | 11461 (37.1) | 106912 (36.5) |
|  | Obese | 6270 (20.3) | 55220 (18.8) |
|  | Missing | 2341 ( 7.6) | 36899 (12.6) |
|  |  |  |  |
| **History of mental illness** | Anxiety | 5890 (19.1) | 53532 (18.3) |
|  | Depression | 7356 (23.8) | 66823 (22.8) |
|  | Non-fatal self-harm | 570 ( 1.8) | 4254 ( 1.5) |

### Central nervous system (CNS, C71-72)

|  |  | **Cancer survivors** | **Non-cancer comparators** |
| --- | --- | --- | --- |
| **Total** |  | 13524 (100.0) | 129287 (100.0) |
| **Person-years from cancer diagnosis/baseline to end of follow-up** | |  |  |
|  | Mean (SD) | 1.7 (2.9) | 7.3 (5.1) |
|  | Median (IQR) | 1.7 (0.2-1.5) | 7.3 (3.2-10.6) |
|  | Range | 0.0-22.1 | 0.0-22.2 |
| **Total person-years included* (millions)** | | 0.02 | 0.95 |
|  |  |  |  |
| **Cancer stage at diagnosis** | Early | 1214 ( 9.0) |  |
|  | Late | 6390 (47.2) |  |
|  | Missing | 5920 (43.8) |  |
|  |  |  |  |
| **Age (years)** | 18-39 | 1496 (11.1) | 13884 (10.7) |
|  | 40-59 | 3961 (29.3) | 37700 (29.2) |
|  | 60-79 | 6396 (47.3) | 61893 (47.9) |
|  | >=80 | 1671 (12.4) | 15809 (12.2) |
|  |  |  |  |
| **Sex** | Male | 7784 (57.6) | 73839 (57.1) |
|  | Female | 5740 (42.4) | 55448 (42.9) |
|  |  |  |  |
| **Index of multiple deprivation** | 1 (least deprived) | 3203 (23.7) | 31036 (24.0) |
|  | 2 | 3142 (23.2) | 29404 (22.7) |
|  | 3 | 2711 (20.0) | 25715 (19.9) |
|  | 4 | 2445 (18.1) | 23140 (17.9) |
|  | 5 (most deprived) | 2023 (15.0) | 19992 (15.5) |
|  |  |  |  |
| **Ethnicity** | White | 5219 (38.6) | 63133 (48.8) |
|  | South Asian | 209 ( 1.5) | 2930 ( 2.3) |
|  | Black | 112 ( 0.8) | 1728 ( 1.3) |
|  | Other | 44 ( 0.3) | 739 ( 0.6) |
|  | Mixed | 29 ( 0.2) | 408 ( 0.3) |
|  | Missing | 7911 (58.5) | 60349 (46.7) |
|  |  |  |  |
| **Calendar year** | 1998-2000 | 852 ( 6.3) | 7648 ( 5.9) |
|  | 2001-2005 | 2766 (20.5) | 25881 (20.0) |
|  | 2006-2010 | 3737 (27.6) | 35875 (27.7) |
|  | 2011-2015 | 3827 (28.3) | 37186 (28.8) |
|  | 2016-2018 | 2342 (17.3) | 22697 (17.6) |
|  |  |  |  |
| **Consultations in year prior to index date** | 0 | 797 ( 5.9) | 20835 (16.1) |
|  | 1 to 3 | 1289 ( 9.5) | 28969 (22.4) |
|  | 4 to 9 | 3754 (27.8) | 38369 (29.7) |
|  | 10+ | 7684 (56.8) | 41107 (31.8) |
|  |  |  |  |
| **Smoking status** | Non-smoker | 4934 (36.5) | 44200 (34.2) |
|  | Current smoker | 2131 (15.8) | 24729 (19.1) |
|  | Ex-smoker | 6459 (47.8) | 60358 (46.7) |
|  |  |  |  |
| **Problematic alcohol use** | No | 13171 (97.4) | 125195 (96.8) |
|  | Yes | 353 ( 2.6) | 4092 ( 3.2) |
|  |  |  |  |
| **BMI category** | Underweight | 193 ( 1.4) | 2025 ( 1.6) |
|  | Normal weight | 4362 (32.3) | 41586 (32.2) |
|  | Overweight | 4745 (35.1) | 45218 (35.0) |
|  | Obese | 2899 (21.4) | 27213 (21.0) |
|  | Missing | 1325 ( 9.8) | 13245 (10.2) |
|  |  |  |  |
| **History of mental illness** | Anxiety | 3459 (25.6) | 28201 (21.8) |
|  | Depression | 3941 (29.1) | 32915 (25.5) |
|  | Non-fatal self-harm | 335 ( 2.5) | 3201 ( 2.5) |

### Thyroid (C73)

|  |  | **Cancer survivors** | **Non-cancer comparators** |
| --- | --- | --- | --- |
| **Total** |  | 7681 (100.0) | 73911 (100.0) |
| **Person-years from cancer diagnosis/baseline to end of follow-up** | |  |  |
|  | Mean (SD) | 6.3 (5.1) | 6.9 (4.9) |
|  | Median (IQR) | 6.3 (2.4-9.2) | 6.9 (3.1-9.9) |
|  | Range | 0.0-22.2 | 0.0-22.2 |
| **Total person-years included* (millions)** | | 0.05 | 0.51 |
|  |  |  |  |
| **Cancer stage at diagnosis** | Early | 2545 (33.1) |  |
|  | Late | 1288 (16.8) |  |
|  | Missing | 3848 (50.1) |  |
|  |  |  |  |
| **Age (years)** | 18-39 | 1896 (24.7) | 18097 (24.5) |
|  | 40-59 | 3100 (40.4) | 29915 (40.5) |
|  | 60-79 | 2131 (27.7) | 20666 (28.0) |
|  | >=80 | 554 ( 7.2) | 5233 ( 7.1) |
|  |  |  |  |
| **Sex** | Male | 2007 (26.1) | 18991 (25.7) |
|  | Female | 5674 (73.9) | 54920 (74.3) |
|  |  |  |  |
| **Index of multiple deprivation** | 1 (least deprived) | 1731 (22.5) | 16504 (22.3) |
|  | 2 | 1642 (21.4) | 15545 (21.0) |
|  | 3 | 1545 (20.1) | 14555 (19.7) |
|  | 4 | 1497 (19.5) | 14513 (19.6) |
|  | 5 (most deprived) | 1266 (16.5) | 12794 (17.3) |
|  |  |  |  |
| **Ethnicity** | White | 3073 (40.0) | 35102 (47.5) |
|  | South Asian | 420 ( 5.5) | 3330 ( 4.5) |
|  | Black | 166 ( 2.2) | 1876 ( 2.5) |
|  | Other | 125 ( 1.6) | 861 ( 1.2) |
|  | Mixed | 52 ( 0.7) | 433 ( 0.6) |
|  | Missing | 3845 (50.1) | 32309 (43.7) |
|  |  |  |  |
| **Calendar year** | 1998-2000 | 335 ( 4.4) | 3052 ( 4.1) |
|  | 2001-2005 | 1172 (15.3) | 11003 (14.9) |
|  | 2006-2010 | 1976 (25.7) | 19062 (25.8) |
|  | 2011-2015 | 2549 (33.2) | 24775 (33.5) |
|  | 2016-2018 | 1649 (21.5) | 16019 (21.7) |
|  |  |  |  |
| **Consultations in year prior to index date** | 0 | 341 ( 4.4) | 11455 (15.5) |
|  | 1 to 3 | 630 ( 8.2) | 17560 (23.8) |
|  | 4 to 9 | 2323 (30.2) | 22914 (31.0) |
|  | 10+ | 4387 (57.1) | 21977 (29.7) |
|  |  |  |  |
| **Smoking status** | Non-smoker | 3231 (42.1) | 28749 (38.9) |
|  | Current smoker | 1016 (13.2) | 14100 (19.1) |
|  | Ex-smoker | 3434 (44.7) | 31062 (42.0) |
|  |  |  |  |
| **Problematic alcohol use** | No | 7513 (97.8) | 71702 (97.0) |
|  | Yes | 168 ( 2.2) | 2209 ( 3.0) |
|  |  |  |  |
| **BMI category** | Underweight | 149 ( 1.9) | 1511 ( 2.0) |
|  | Normal weight | 2796 (36.4) | 27297 (36.9) |
|  | Overweight | 2445 (31.8) | 22434 (30.4) |
|  | Obese | 1900 (24.7) | 16139 (21.8) |
|  | Missing | 391 ( 5.1) | 6530 ( 8.8) |
|  |  |  |  |
| **History of mental illness** | Anxiety | 2129 (27.7) | 19355 (26.2) |
|  | Depression | 2397 (31.2) | 22036 (29.8) |
|  | Non-fatal self-harm | 244 ( 3.2) | 2582 ( 3.5) |

### Non-Hodgkin Lymphoma (NHL, C82-85)

|  |  | **Cancer survivors** | **Non-cancer comparators** |
| --- | --- | --- | --- |
| **Total** |  | 35606 (100.0) | 340697 (100.0) |
| **Person-years from cancer diagnosis/baseline to end of follow-up** | |  |  |
|  | Mean (SD) | 4.7 (4.7) | 6.9 (5.0) |
|  | Median (IQR) | 4.7 (0.7-7.1) | 6.9 (3.0-9.9) |
|  | Range | 0.0-22.2 | 0.0-22.2 |
| **Total person-years included* (millions)** | | 0.17 | 2.36 |
|  |  |  |  |
| **Cancer stage at diagnosis** | Early | 5022 (14.1) |  |
|  | Late | 9710 (27.3) |  |
|  | Missing | 20874 (58.6) |  |
|  |  |  |  |
| **Age (years)** | 18-39 | 1684 ( 4.7) | 15660 ( 4.6) |
|  | 40-59 | 8013 (22.5) | 76291 (22.4) |
|  | 60-79 | 18472 (51.9) | 178446 (52.4) |
|  | >=80 | 7437 (20.9) | 70300 (20.6) |
|  |  |  |  |
| **Sex** | Male | 19031 (53.4) | 180952 (53.1) |
|  | Female | 16575 (46.6) | 159745 (46.9) |
|  |  |  |  |
| **Index of multiple deprivation** | 1 (least deprived) | 8518 (23.9) | 81619 (24.0) |
|  | 2 | 8007 (22.5) | 76907 (22.6) |
|  | 3 | 7159 (20.1) | 69109 (20.3) |
|  | 4 | 6475 (18.2) | 61284 (18.0) |
|  | 5 (most deprived) | 5447 (15.3) | 51778 (15.2) |
|  |  |  |  |
| **Ethnicity** | White | 13765 (38.7) | 169399 (49.7) |
|  | South Asian | 689 ( 1.9) | 7767 ( 2.3) |
|  | Black | 446 ( 1.3) | 4986 ( 1.5) |
|  | Other | 175 ( 0.5) | 1853 ( 0.5) |
|  | Mixed | 87 ( 0.2) | 1079 ( 0.3) |
|  | Missing | 20444 (57.4) | 155613 (45.7) |
|  |  |  |  |
| **Calendar year** | 1998-2000 | 2207 ( 6.2) | 19729 ( 5.8) |
|  | 2001-2005 | 7250 (20.4) | 67934 (19.9) |
|  | 2006-2010 | 9583 (26.9) | 92180 (27.1) |
|  | 2011-2015 | 10344 (29.1) | 100408 (29.5) |
|  | 2016-2018 | 6222 (17.5) | 60446 (17.7) |
|  |  |  |  |
| **Consultations in year prior to index date** | 0 | 1755 ( 4.9) | 46139 (13.5) |
|  | 1 to 3 | 2497 ( 7.0) | 67157 (19.7) |
|  | 4 to 9 | 8679 (24.4) | 102052 (30.0) |
|  | 10+ | 22675 (63.7) | 125343 (36.8) |
|  |  |  |  |
| **Smoking status** | Non-smoker | 12058 (33.9) | 114973 (33.7) |
|  | Current smoker | 5447 (15.3) | 60984 (17.9) |
|  | Ex-smoker | 18101 (50.8) | 164740 (48.4) |
|  |  |  |  |
| **Problematic alcohol use** | No | 34735 (97.6) | 331208 (97.2) |
|  | Yes | 871 ( 2.4) | 9489 ( 2.8) |
|  |  |  |  |
| **BMI category** | Underweight | 736 ( 2.1) | 5603 ( 1.6) |
|  | Normal weight | 12676 (35.6) | 108087 (31.7) |
|  | Overweight | 12566 (35.3) | 118976 (34.9) |
|  | Obese | 7470 (21.0) | 71457 (21.0) |
|  | Missing | 2158 ( 6.1) | 36574 (10.7) |
|  |  |  |  |
| **History of mental illness** | Anxiety | 8138 (22.9) | 73814 (21.7) |
|  | Depression | 9941 (27.9) | 88908 (26.1) |
|  | Non-fatal self-harm | 846 ( 2.4) | 7304 ( 2.1) |

### Multiple myeloma (C90)

|  |  | **Cancer survivors** | **Non-cancer comparators** |
| --- | --- | --- | --- |
| **Total** |  | 14517 (100.0) | 138963 (100.0) |
| **Person-years from cancer diagnosis/baseline to end of follow-up** | |  |  |
|  | Mean (SD) | 3.4 (3.5) | 6.5 (4.7) |
|  | Median (IQR) | 3.4 (0.6-5.0) | 6.5 (2.9-9.3) |
|  | Range | 0.0-22.2 | 0.0-22.2 |
| **Total person-years included* (millions)** | | 0.05 | 0.91 |
|  |  |  |  |
| **Cancer stage at diagnosis** | Early | 936 ( 6.4) |  |
|  | Late | 460 ( 3.2) |  |
|  | Missing | 13121 (90.4) |  |
|  |  |  |  |
| **Age (years)** | 18-39 | 125 ( 0.9) | 1161 ( 0.8) |
|  | 40-59 | 2369 (16.3) | 22624 (16.3) |
|  | 60-79 | 8250 (56.8) | 79575 (57.3) |
|  | >=80 | 3773 (26.0) | 35603 (25.6) |
|  |  |  |  |
| **Sex** | Male | 7966 (54.9) | 76066 (54.7) |
|  | Female | 6551 (45.1) | 62897 (45.3) |
|  |  |  |  |
| **Index of multiple deprivation** | 1 (least deprived) | 3377 (23.3) | 32635 (23.5) |
|  | 2 | 3295 (22.7) | 31249 (22.5) |
|  | 3 | 2916 (20.1) | 28224 (20.3) |
|  | 4 | 2726 (18.8) | 25433 (18.3) |
|  | 5 (most deprived) | 2203 (15.2) | 21422 (15.4) |
|  |  |  |  |
| **Ethnicity** | White | 5632 (38.8) | 71037 (51.1) |
|  | South Asian | 280 ( 1.9) | 3391 ( 2.4) |
|  | Black | 495 ( 3.4) | 2618 ( 1.9) |
|  | Other | 47 ( 0.3) | 754 ( 0.5) |
|  | Mixed | 66 ( 0.5) | 455 ( 0.3) |
|  | Missing | 7997 (55.1) | 60708 (43.7) |
|  |  |  |  |
| **Calendar year** | 1998-2000 | 883 ( 6.1) | 7902 ( 5.7) |
|  | 2001-2005 | 2843 (19.6) | 26530 (19.1) |
|  | 2006-2010 | 3851 (26.5) | 37069 (26.7) |
|  | 2011-2015 | 4317 (29.7) | 41898 (30.2) |
|  | 2016-2018 | 2623 (18.1) | 25564 (18.4) |
|  |  |  |  |
| **Consultations in year prior to index date** | 0 | 612 ( 4.2) | 15941 (11.5) |
|  | 1 to 3 | 624 ( 4.3) | 25278 (18.2) |
|  | 4 to 9 | 2606 (18.0) | 41809 (30.1) |
|  | 10+ | 10675 (73.5) | 55935 (40.3) |
|  |  |  |  |
| **Smoking status** | Non-smoker | 5096 (35.1) | 45687 (32.9) |
|  | Current smoker | 1831 (12.6) | 24229 (17.4) |
|  | Ex-smoker | 7590 (52.3) | 69047 (49.7) |
|  |  |  |  |
| **Problematic alcohol use** | No | 14223 (98.0) | 135269 (97.3) |
|  | Yes | 294 ( 2.0) | 3694 ( 2.7) |
|  |  |  |  |
| **BMI category** | Underweight | 290 ( 2.0) | 2342 ( 1.7) |
|  | Normal weight | 4793 (33.0) | 42876 (30.9) |
|  | Overweight | 5277 (36.4) | 49240 (35.4) |
|  | Obese | 3114 (21.5) | 29327 (21.1) |
|  | Missing | 1043 ( 7.2) | 15178 (10.9) |
|  |  |  |  |
| **History of mental illness** | Anxiety | 3417 (23.5) | 29648 (21.3) |
|  | Depression | 4098 (28.2) | 36231 (26.1) |
|  | Non-fatal self-harm | 254 ( 1.7) | 2573 ( 1.9) |

### Leukaemia (C91-C95)

|  |  | **Cancer survivors** | **Non-cancer comparators** |
| --- | --- | --- | --- |
| **Total** |  | 23624 (100.0) | 225339 (100.0) |
| **Person-years from cancer diagnosis/baseline to end of follow-up** | |  |  |
|  | Mean (SD) | 3.7 (4.3) | 6.6 (4.8) |
|  | Median (IQR) | 3.7 (0.4-5.6) | 6.6 (2.9-9.4) |
|  | Range | 0.0-22.2 | 0.0-22.2 |
| **Total person-years included* (millions)** | | 0.09 | 1.5 |
|  |  |  |  |
| **Cancer stage at diagnosis** | Early | 1202 ( 5.1) |  |
|  | Late | 111 ( 0.5) |  |
|  | Missing | 22311 (94.4) |  |
|  |  |  |  |
| **Age (years)** | 18-39 | 1176 ( 5.0) | 10899 ( 4.8) |
|  | 40-59 | 4375 (18.5) | 41662 (18.5) |
|  | 60-79 | 12055 (51.0) | 116417 (51.7) |
|  | >=80 | 6018 (25.5) | 56361 (25.0) |
|  |  |  |  |
| **Sex** | Male | 13847 (58.6) | 131563 (58.4) |
|  | Female | 9777 (41.4) | 93776 (41.6) |
|  |  |  |  |
| **Index of multiple deprivation** | 1 (least deprived) | 5561 (23.5) | 53112 (23.6) |
|  | 2 | 5259 (22.3) | 50541 (22.4) |
|  | 3 | 4730 (20.0) | 45644 (20.3) |
|  | 4 | 4402 (18.6) | 40903 (18.2) |
|  | 5 (most deprived) | 3672 (15.5) | 35139 (15.6) |
|  |  |  |  |
| **Ethnicity** | White | 9466 (40.1) | 113789 (50.5) |
|  | South Asian | 392 ( 1.7) | 4985 ( 2.2) |
|  | Black | 285 ( 1.2) | 2998 ( 1.3) |
|  | Other | 86 ( 0.4) | 1212 ( 0.5) |
|  | Mixed | 49 ( 0.2) | 722 ( 0.3) |
|  | Missing | 13346 (56.5) | 101633 (45.1) |
|  |  |  |  |
| **Calendar year** | 1998-2000 | 1459 ( 6.2) | 12898 ( 5.7) |
|  | 2001-2005 | 4676 (19.8) | 43483 (19.3) |
|  | 2006-2010 | 6383 (27.0) | 61380 (27.2) |
|  | 2011-2015 | 7068 (29.9) | 68452 (30.4) |
|  | 2016-2018 | 4038 (17.1) | 39126 (17.4) |
|  |  |  |  |
| **Consultations in year prior to index date** | 0 | 1137 ( 4.8) | 28386 (12.6) |
|  | 1 to 3 | 1951 ( 8.3) | 42875 (19.0) |
|  | 4 to 9 | 6180 (26.2) | 67804 (30.1) |
|  | 10+ | 14356 (60.8) | 86272 (38.3) |
|  |  |  |  |
| **Smoking status** | Non-smoker | 7845 (33.2) | 74951 (33.3) |
|  | Current smoker | 3427 (14.5) | 39696 (17.6) |
|  | Ex-smoker | 12352 (52.3) | 110692 (49.1) |
|  |  |  |  |
| **Problematic alcohol use** | No | 23038 (97.5) | 219157 (97.3) |
|  | Yes | 586 ( 2.5) | 6182 ( 2.7) |
|  |  |  |  |
| **BMI category** | Underweight | 449 ( 1.9) | 3812 ( 1.7) |
|  | Normal weight | 7758 (32.8) | 70979 (31.5) |
|  | Overweight | 8529 (36.1) | 79113 (35.1) |
|  | Obese | 5086 (21.5) | 45570 (20.2) |
|  | Missing | 1802 ( 7.6) | 25865 (11.5) |
|  |  |  |  |
| **History of mental illness** | Anxiety | 5141 (21.8) | 47068 (20.9) |
|  | Depression | 6402 (27.1) | 57110 (25.3) |
|  | Non-fatal self-harm | 491 ( 2.1) | 4464 ( 2.0) |

# **S4 Results** Numbers of cancer survivors and cancer-free controls included in each analysis

|  | Anxiety | | **Depression** | | **Non-fatal self-harm** | | **Completed Suicide** | |
| --- | --- | --- | --- | --- | --- | --- | --- | --- |
| **Cancer type or site (ICD-10)** | **Cancer survivors** | **Cancer-free controls** | **Cancer survivors** | **Cancer-free controls** | **Cancer survivors** | **Cancer-free controls** | **Cancer survivors** | **Cancer-free controls** |
| Oral cavity (C00-06) | 10478 | 104195 | 10108 | 101063 | 11945 | 114184 | 12001 | 114423 |
| Oesophagus (C15) | 21930 | 214369 | 21142 | 207145 | 24372 | 233069 | 24430 | 233412 |
| Stomach (C16) | 19351 | 186685 | 18569 | 180538 | 21317 | 202378 | 21364 | 202674 |
| Colorectal (C18-20) | 106820 | 1024766 | 103292 | 989498 | 117789 | 1121652 | 117988 | 1123544 |
| Liver (C22) | 10154 | 101859 | 9610 | 97991 | 11630 | 111577 | 11671 | 111740 |
| Pancreas (C25) | 21263 | 210555 | 20094 | 202556 | 24162 | 231534 | 24224 | 231873 |
| Lung (C34) | 96793 | 976554 | 91483 | 940457 | 112752 | 1071588 | 113178 | 1073350 |
| Malignant melanoma (C43) | 34190 | 326396 | 33255 | 316627 | 37506 | 360317 | 37587 | 361204 |
| Breast (C50) | 132759 | 1285924 | 128083 | 1236927 | 151695 | 1461604 | 152129 | 1465725 |
| Cervix (C53) | 7596 | 74654 | 7458 | 72604 | 8793 | 84939 | 8838 | 85280 |
| Uterus (C54-55) | 20906 | 201940 | 20265 | 193427 | 23651 | 228851 | 23696 | 229369 |
| Ovary (C56) | 17815 | 174507 | 17280 | 167699 | 20438 | 197321 | 20503 | 197831 |
| Prostate (C61) | 123666 | 1137067 | 120916 | 1108440 | 132129 | 1210522 | 132283 | 1211868 |
| Kidney (C64) | 21714 | 217832 | 20909 | 210909 | 24817 | 238599 | 24901 | 239002 |
| Bladder (C67) | 28237 | 271171 | 27338 | 262550 | 30858 | 292689 | 30911 | 293078 |
| CNS (C71-72) | 11440 | 118050 | 11114 | 114701 | 13482 | 128994 | 13524 | 129287 |
| Thyroid (C73) | 6692 | 65788 | 6571 | 63965 | 7659 | 73672 | 7681 | 73911 |
| NHL (C82-85) | 31800 | 310656 | 30474 | 300321 | 35532 | 340055 | 35606 | 340697 |
| Multiple myeloma (C90) | 12709 | 126894 | 12254 | 122537 | 14491 | 138752 | 14517 | 138963 |
| Leukaemia (C91-95) | 21274 | 206017 | 20398 | 199332 | 23580 | 224935 | 23624 | 2253 |

# **S5 Results** Absolute incidence of mental health outcomes in cancer survivors and comparators, by baseline characteristics

|  |  | **Anxiety** | | **Depression** | | **Non-fatal self-harm** | | **Completed Suicide** | |
| --- | --- | --- | --- | --- | --- | --- | --- | --- | --- |
| Variable |  | Rate cancer survivors | Rate cancer free comparators | Rate cancer survivors | Rate cancer free comparators | Rate cancer survivors | Rate cancer free comparators | Rate cancer survivors | Rate cancer free comparators |
| Age | Age 18-59y | 20.81 | 14.58 | 30.09 | 20.42 | 1.37 | 1.10 | 0.08 | 0.07 |
|  | Age 60y+ | 15.45 | 11.14 | 27.61 | 18.41 | 0.80 | 0.53 | 0.12 | 0.07 |
| History mental illness | No | 11.86 | 7.80 | 21.45 | 13.61 | 0.57 | 0.32 | 0.07 | 0.05 |
|  | Yes | 36.71 | 27.77 | 58.02 | 40.96 | 2.00 | 1.71 | 0.18 | 0.12 |
| Deprivation | Least Deprived | 15.93 | 11.46 | 25.65 | 17.18 | 0.83 | 0.57 | 0.11 | 0.06 |
|  | Most Deprived | 20.02 | 13.97 | 34.78 | 23.16 | 1.36 | 1.07 | 0.10 | 0.08 |
| Calendar Year | Years 1998-2010 | 12.62 | 10.56 | 23.87 | 17.52 | 0.78 | 0.65 | 0.09 | 0.07 |
|  | Years 2011-2018 | 18.38 | 12.64 | 29.59 | 19.38 | 1.05 | 0.75 | 0.11 | 0.07 |
| Regio in England | North | 20.60 | 14.25 | 32.36 | 21.30 | 1.23 | 0.89 | 0.10 | 0.07 |
|  | East | 16.92 | 11.80 | 29.28 | 19.13 | 0.87 | 0.66 | 0.10 | 0.06 |
|  | West | 16.57 | 12.22 | 27.14 | 18.50 | 1.01 | 0.71 | 0.11 | 0.06 |
|  | South | 15.58 | 11.27 | 26.88 | 18.19 | 0.90 | 0.68 | 0.11 | 0.07 |
|  | London | 15.98 | 11.15 | 26.01 | 17.26 | 0.86 | 0.62 | 0.10 | 0.07 |
| Ethnicity | White | 17.29 | 12.85 | 26.81 | 18.91 | 0.93 | 0.73 | 0.07 | 0.05 |
|  | Non-white | 16.99 | 10.81 | 32.13 | 19.39 | 1.16 | 0.74 | 0.18 | 0.12 |
| Sex | Male | 12.39 | 8.10 | 25.59 | 15.29 | 0.94 | 0.61 | 0.17 | 0.11 |
|  | Female | 21.57 | 15.81 | 31.02 | 22.32 | 1.05 | 0.83 | 0.06 | 0.04 |

**Note: Rate per 1000 person years**

# **S6** **Results** Relative risk of mental health outcomes in cancer survivors compared with general population comparators, with effect modification.

1. Anxiety


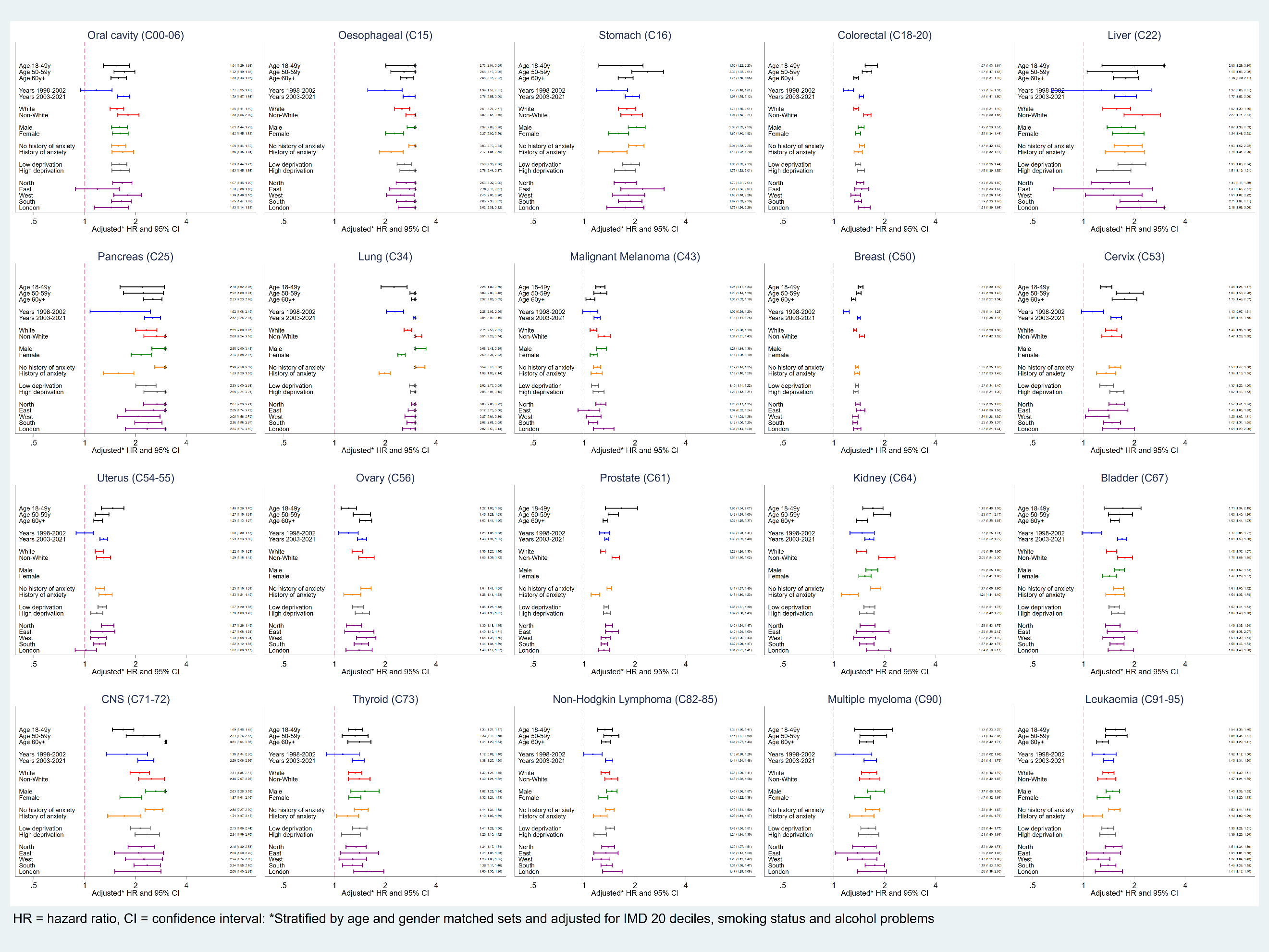


1. Depression


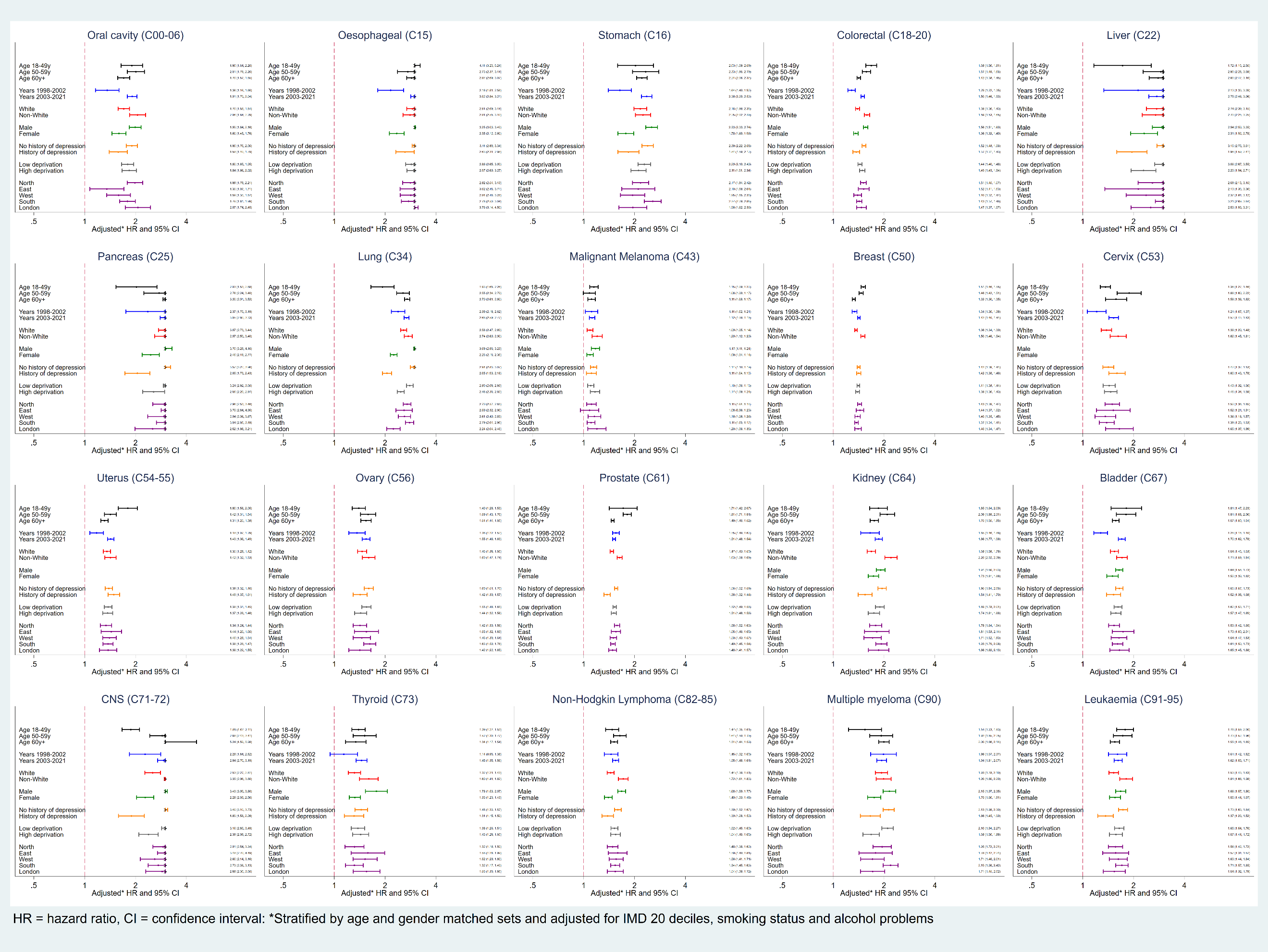


1. Non-fatal self-harm


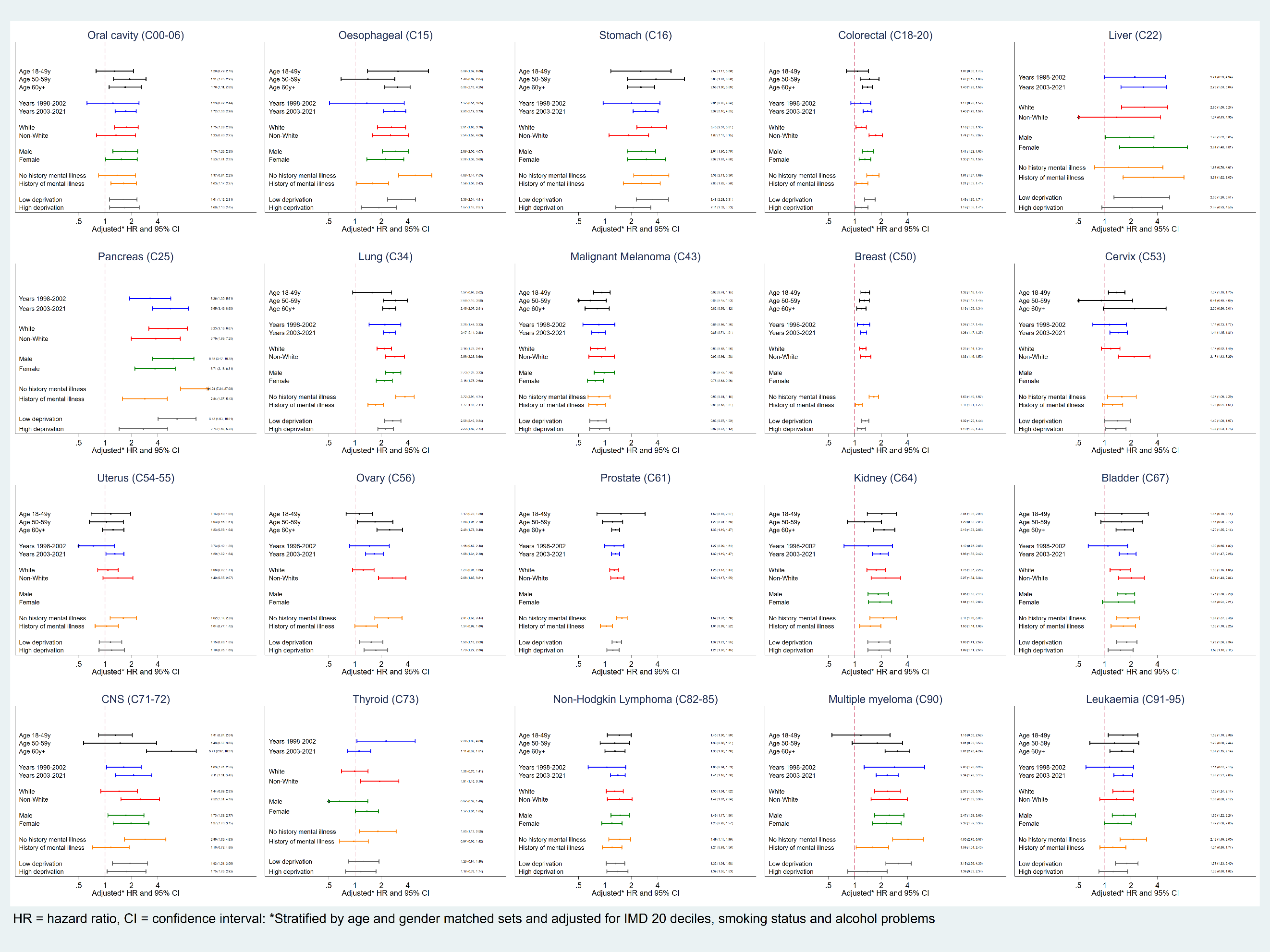


1. Completed Suicide


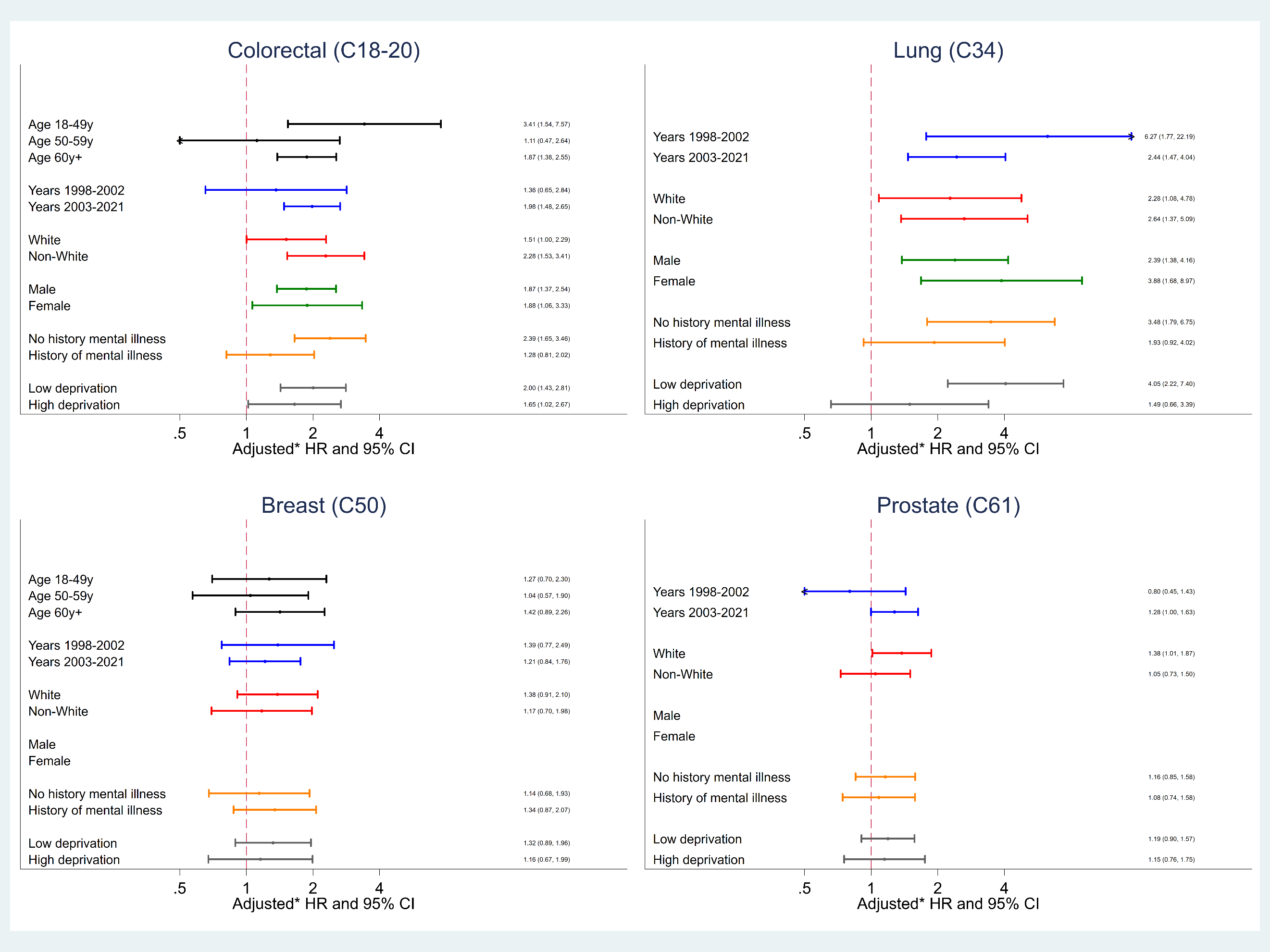


# **S7 Results** Role of time since cancer diagnosis in the associations between cancer survivorship and mental health outcomes in individuals with history of cancer compared to cancer-free individuals.


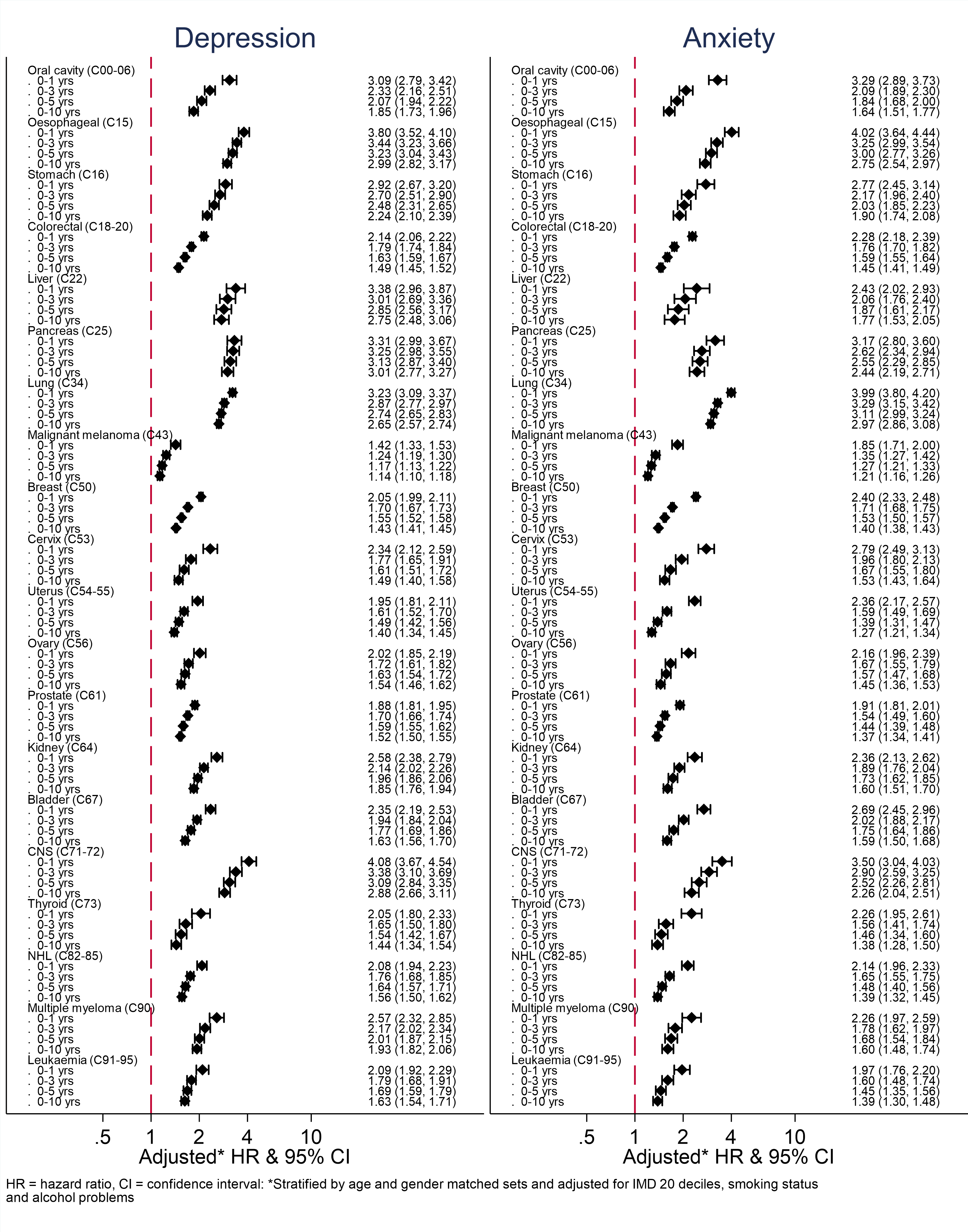


**
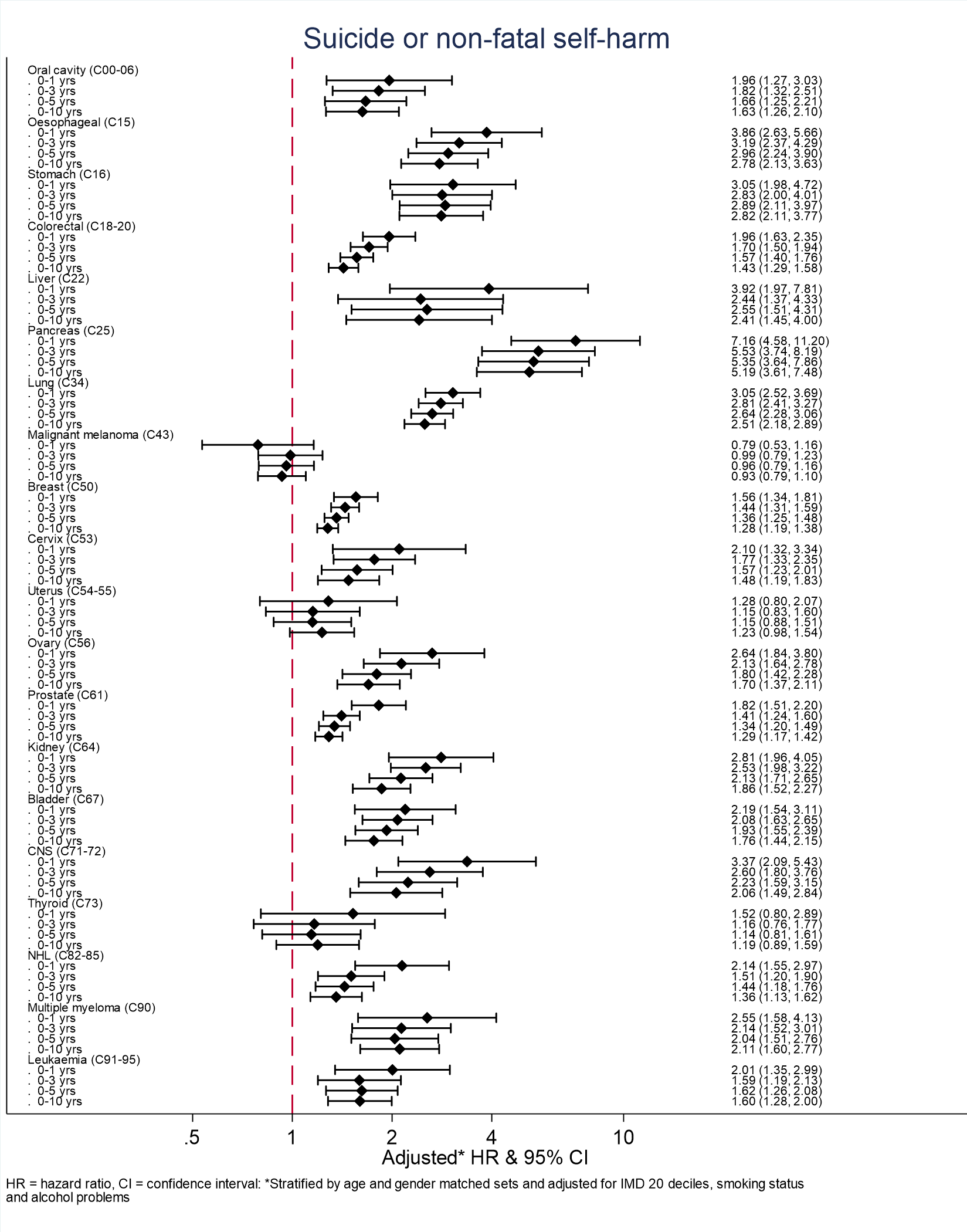
**

# **S8 Results** Sensitivity analyses

We tested how robust our findings were in multiple sensitivity analyses:

1. additionally adjusting for ethnicity derived from CPRD data among those with complete data;
2. additionally adjusting for ethnicity derived from the primary care record, with missingness included as a category
3. additionally adjusting for ethnicity derived from CPRD or HES data among those with complete data; additionally adjusting for BMI category among those with complete data;
4. additionally adjusting for BMI category, with missingness included as a category
5. excluding those with the outcome ever before index date;
6. using a more specific outcome definition (depression and anxiety only) which excluded symptom, monitoring or checklist codes;
7. limiting the comparator group to recent consulters (those with a consultation in the year before index date) to reduce the potential for ascertainment bias;
8. additionally adjusting for history of mental illness (depression, anxiety or non-fatal self-harm) before index date and
9. censoring follow-up on 31st January 2020 (start of the COVID-19 pandemic, when primary care contacts for mental health conditions reduced^19^).

### Depression


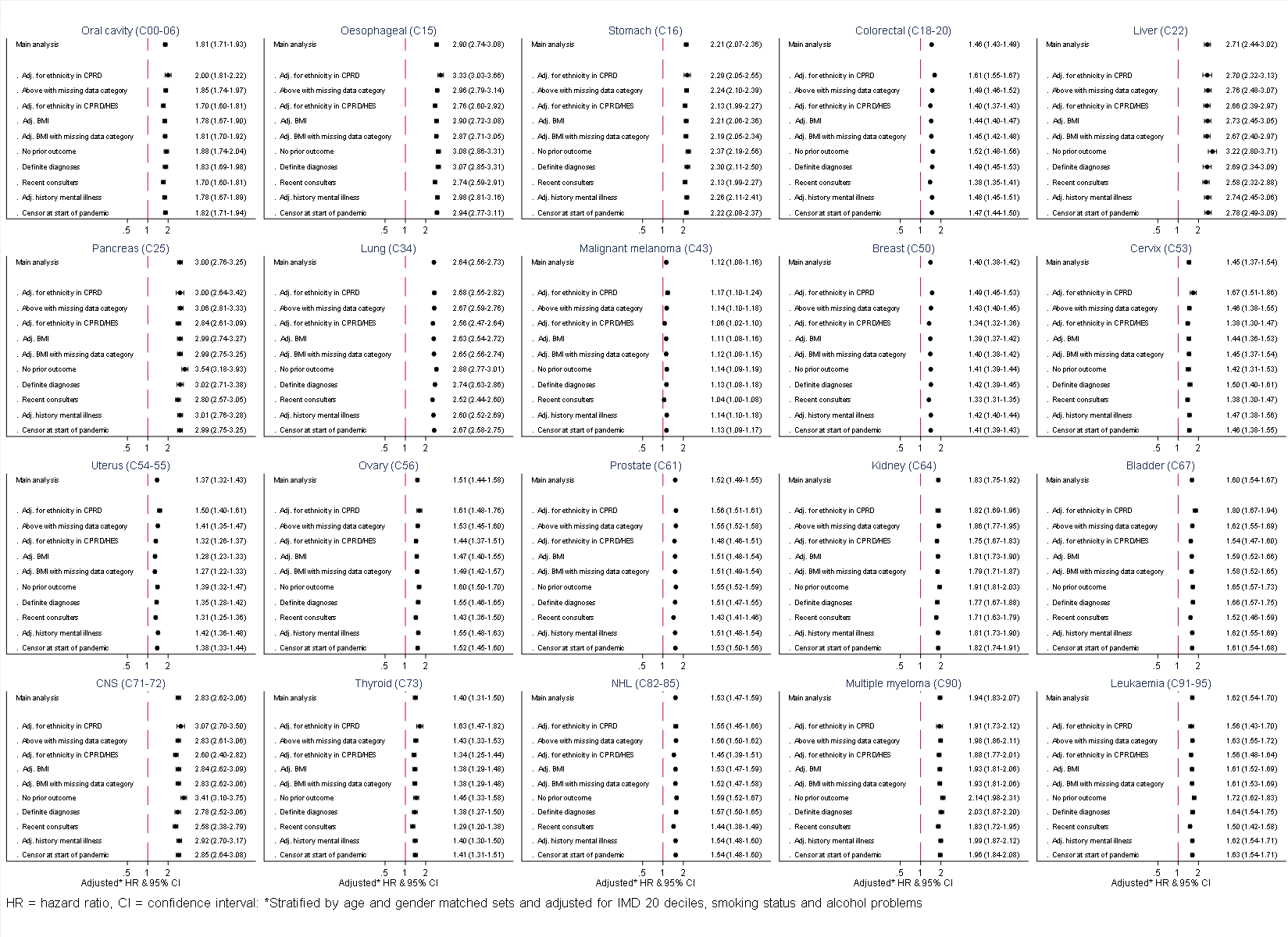


### Anxiety


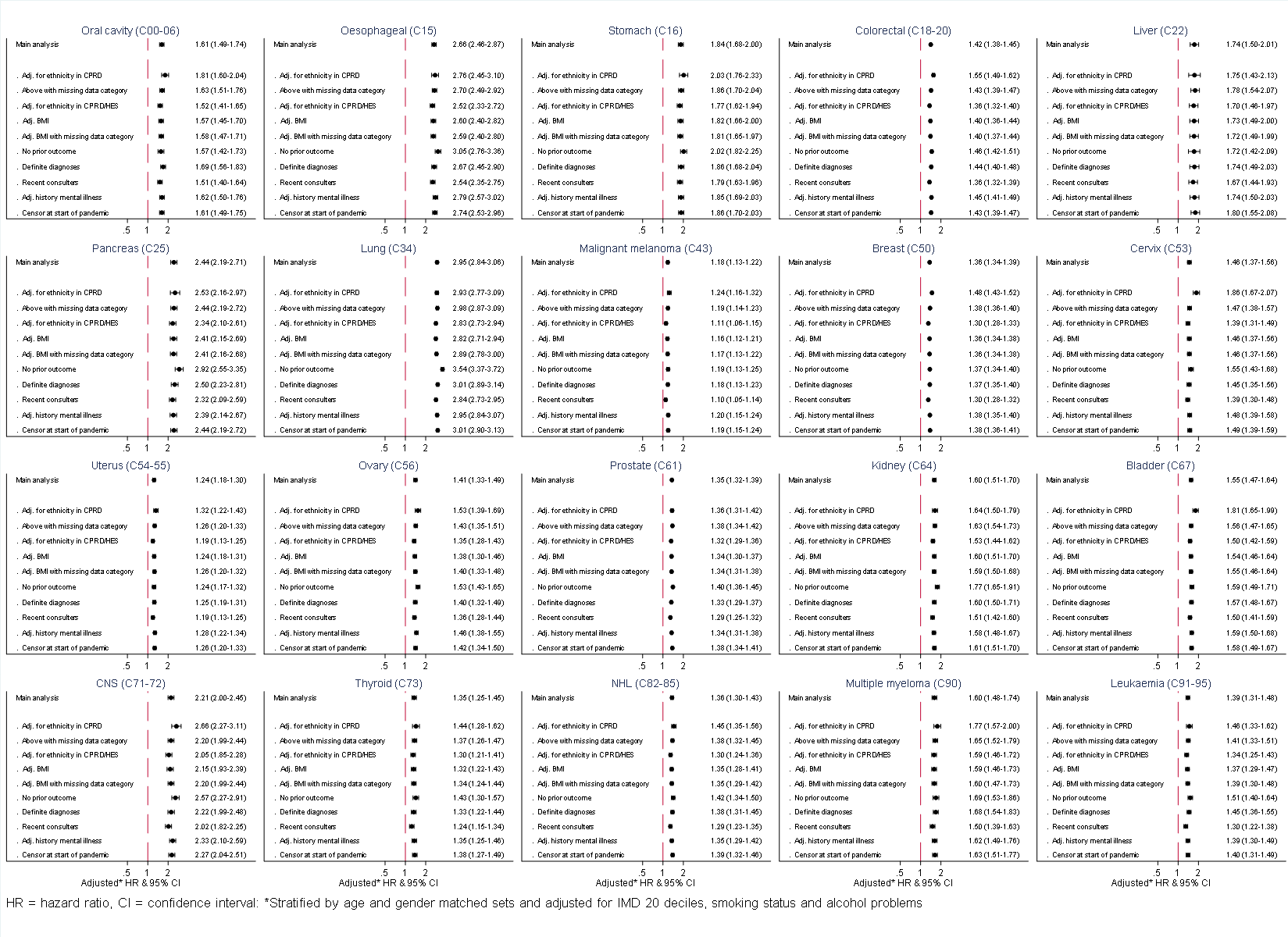


### Non-fatal self-harm


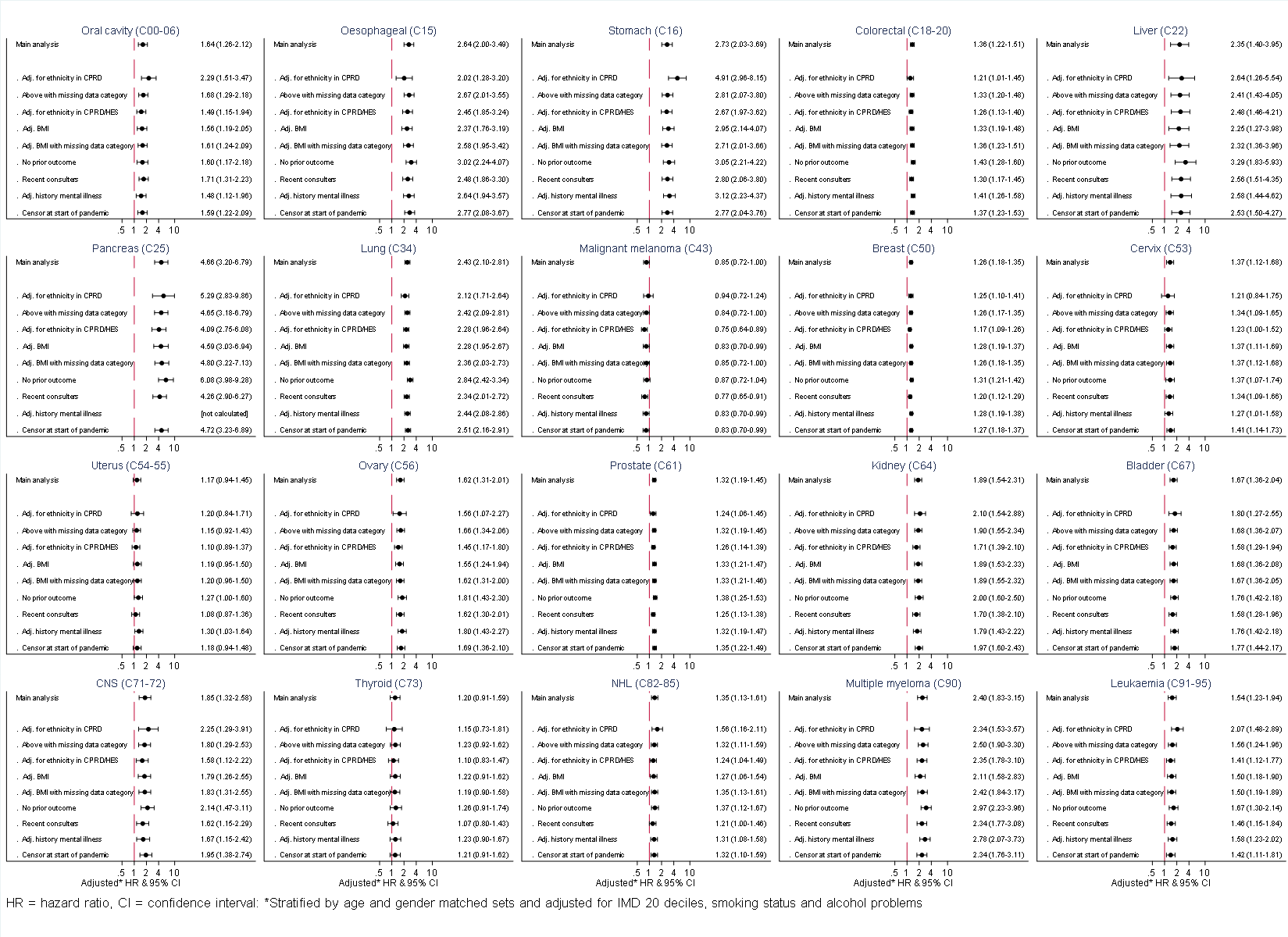


### Completed suicide


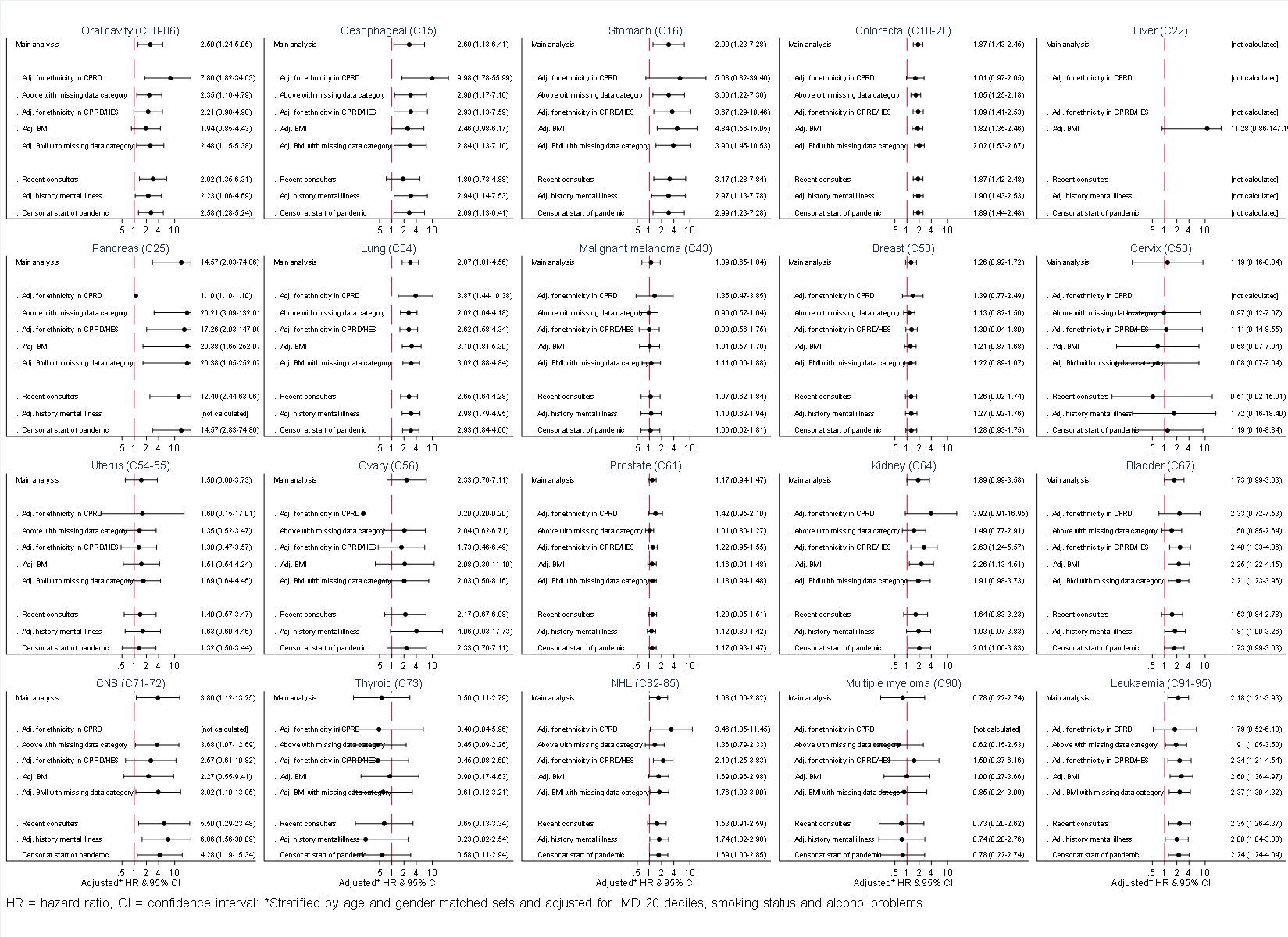


# References

1. Carreira, H., et al., *Associations between breast cancer survivorship and adverse mental health outcomes: A matched population-based cohort study in the United Kingdom.* PLOS Medicine, 2021. **18**(1): p. e1003504.

2. Thomas, K.H., et al., *Validation of Completed Suicide and self-harm records in the Clinical Practice Research Datalink.* Br J Clin Pharmacol, 2013. **76**(1): p. 145-57.

3. Marchant, A., et al., *Self-harm presentation across healthcare settings by sex in young people: an e-cohort study using routinely collected linked healthcare data in Wales, UK.* Arch Dis Child, 2020. **105**(4): p. 347-354.
